# Cytotoxicity and Antimycobacterial Properties of Pyrrolo[1,2-*a*]quinoline Derivatives: Molecular Target Identification and Molecular Docking Studies

**DOI:** 10.3390/antibiotics9050233

**Published:** 2020-05-07

**Authors:** Katharigatta N. Venugopala, Vijayakumar Uppar, Sandeep Chandrashekharappa, Hassan H. Abdallah, Melendhran Pillay, Pran Kishore Deb, Mohamed A. Morsy, Bandar E. Aldhubiab, Mahesh Attimarad, Anroop B. Nair, Nagaraja Sreeharsha, Christophe Tratrat, Abdulmuttaleb Yousef Jaber, Rashmi Venugopala, Raghu Prasad Mailavaram, Bilal A. Al-Jaidi, Mahmoud Kandeel, Michelyne Haroun, Basavaraj Padmashali

**Affiliations:** 1 Department of Pharmaceutical Sciences, College of Clinical Pharmacy, King Faisal University, Al-Ahsa 31982, Saudi Arabia; momorsy@kfu.edu.sa (M.A.M.); baldhubiab@kfu.edu.sa (B.E.A.); mattimarad@kfu.edu.sa (M.A.); anair@kfu.edu.sa (A.B.N.); sharsha@kfu.edu.sa (N.S.); ctratrat@kfu.edu.sa (C.T.); mharoun@kfu.edu.sa (M.H.); 2 Department of Biotechnology and Food Technology, Durban University of Technology, Durban 4001, South Africa; 3 Department of Chemistry, School of Basic Science, Rani Channamma University, Belagavi 591156, India; vijay.uppar@gmail.com (V.U.); basavarajpadmashali@yahoo.com (B.P.); 4 Institute for Stem Cell Biology and Regenerative Medicine, National Center for Biological Sciences (NCBS), Tata Institute of Fundamental Research (TIFR), Gandhi Krishi Vignana Kendra (GKVK) Campus, Bangalore 560065, India; sandeepc@instem.res.in; 5 Chemistry Department, College of Education, Salahaddin University, Erbil 44001, Iraq; Hassan.Abdullah@su.edu.krd; 6 Department of Microbiology, National Health Laboratory Services, KZN Academic Complex, Inkosi Albert Luthuli Central Hospital, Durban 4001, South Africa; melendhra.pillay@nhls.ac.za; 7 Faculty of Pharmacy, Philadelphia University, Amman 19392, Jordan; prankishore1@gmail.com (P.K.D.); amyjaber@gmail.com (A.Y.J.); 8 Department of Pharmacology, Faculty of Medicine, Minia University, El-Minia 61511, Egypt; 9 Department of Public Health Medicine, University of KwaZulu-Natal, Howard College Campus, Durban 4001, South Africa; rashmivenugopala@gmail.com; 10 Department of Pharmaceutical Chemistry, Shri Vishnu College of Pharmacy, Vishnupur, Bhimavaram 534 202, India; raghumrp@svcp.edu.in; 11 Faculty of Pharmacy, Yarmouk University, Irbid 21163, Jordan; bilaljeaidi77@gmail.com; 12 Department of Biomedical Sciences, College of Veterinary Medicine, King Faisal University, Al-Ahsa 31982, Saudi Arabia; mkandeel@kfu.edu.sa; 13 Department of Pharmacology, Faculty of Veterinary Medicine, Kafrelsheikh University, Kafrelsheikh 33516, Egypt

**Keywords:** pyrrolo[1,2-*a*]quinoline, *Mycobacterium tuberculosis*, H37Rv, MDR-MTB, minimum inhibitory concentration, cytotoxicity, computational studies, molecular target identification

## Abstract

A series of ethyl 1-(substituted benzoyl)-5-methylpyrrolo[1,2-*a*]quinoline-3-carboxylates **4a**–**f** and dimethyl 1-(substituted benzoyl)-5-methylpyrrolo[1,2-*a*]quinoline-2,3-dicarboxylates **4g**–**k** have been synthesized and evaluated for their anti-tubercular (TB) activities against H37Rv (American Type Culture Collection (ATCC) strain 25177) and multidrug-resistant (MDR) strains of *Mycobacterium tuberculosis* by resazurin microplate assay (REMA). Molecular target identification for these compounds was also carried out by a computational approach. All test compounds exhibited anti-tuberculosis (TB) activity in the range of 8–128 µg/mL against H37Rv. The test compound dimethyl-1-(4-fluorobenzoyl)-5-methylpyrrolo[1,2-*a*]quinoline-2,3-dicarboxylate **4j** emerged as the most promising anti-TB agent against H37Rv and multidrug-resistant strains of *Mycobacterium tuberculosis* at 8 and 16 µg/mL, respectively. In silico evaluation of pharmacokinetic properties indicated overall drug-likeness for most of the compounds. Docking studies were also carried out to investigate the binding affinities as well as interactions of these compounds with the target proteins.

## 1. Introduction

**Figure 1:**
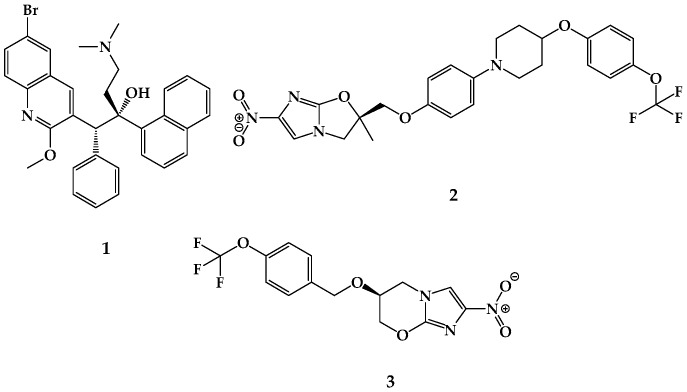
Anti-tubercular drugs 1, 2 and 3, which are approved in last 10 years [22].

Tuberculosis (TB) continues to be one of the top causes of death worldwide. This infectious disease is caused by the organism *Mycobacterium tuberculosis* (MTB) [1]. Approximately 1.3 million deaths were caused by TB in 2018. In addition, people who are human immunodeficiency virus (HIV)-positive found to be highly susceptible to TB infection. Approximately 300,000 deaths of HIV-positive patients were due to TB infection [2]. In addition, the emergence of multidrug-resistant TB (MDR-TB) and extensively drug-resistant TB (XDR-TB) strains, which are highly resistant to most of the currently available anti-TB drugs, trigger the urgent need for the development of novel therapeutic agents to combat these resistant strains [3,4] as only a few drugs are available with United States Food and Drug Administration (US FDA) approval (Figure 1). Reviews focused on *Mycobacterium tuberculosis* and in the pursuit of developing novel anti-TB agents, we recently launched a medicinal chemistry program aimed at developing novel, natural [5], cyclic depsipeptides [6] and various heterocyclic scaffolds as potential anti-TB agents [7,8,9,10,11,12,13], including analogues of indolizine such as pyrrolo[1,2-*a*]quinoline and pyrrolo[1,2-*a*]isoquinoline, also known as 5,6-benzo-fused indolizine and 7,8-benzo-fused indolizine, respectively [14]. These scaffolds have attracted the attention of medical chemists, as they exhibit a wide variety of pharmacological properties [15]. The pyrrolo[1,2-*a*]quinoline derivatives possess antioxidant [16], anti-inflammatory [17,18], larvicidal [19], and antiviral [20] activities. 4-Amino-pyrrolo[2,3-*b*]quinoline has also been tested as a potential treatment for Alzheimer’s disease [21]. These pharmacological potentials of the pyrrolo[1,2-*a*]quinoline scaffold gave us the impetus to further explore this scaffold in the quest of anti-TB drug discovery [5,6,7,8,9,10,11,12]. Herein, we report the evaluation of some novel pyrrolo[1,2-*a*]quinoline derivatives as potential anti-TB agents against H37Rv and MDR strains of *Mycobacterium tuberculosis*. Encouraging bioactivity of these tested compounds against TB strains prompted us to elucidate their mechanism of action by employing molecular docking calculations. The in silico pharmacokinetic profile (absorption, distribution, metabolism, and excretion (ADME) properties) of these compounds was investigated to evaluate their drug-likeness.

## 2. Results and Discussion

### 2.1. Chemistry

**Scheme 1:**
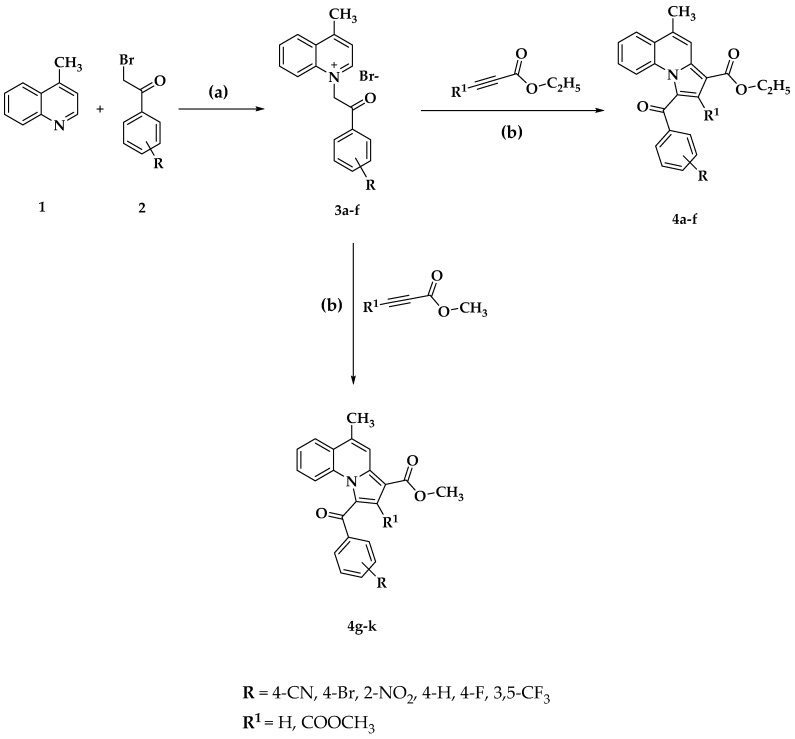
Synthetic scheme for the construction of ethyl 1-(substituted benzoyl)-5-methylpyrrolo[1,2-*a*]quinoline-3-carboxylate **4a**–**f** and dimethyl 1-(substituted benzoyl)-5-methylpyrrolo[1,2-*a*]quinoline-2,3-dicarboxylate **4g**–**k** analogues: Reagents and conditions: (**a**) acetone, r.t., 30 min; (**b**) DMF, K_2_CO_3_, 30 min.

**Figure 2:**
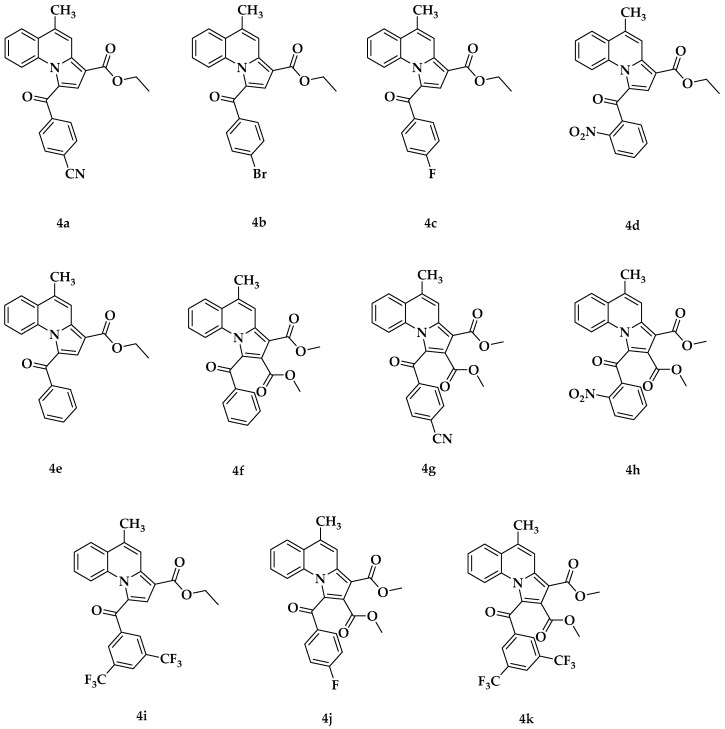
Chemical structure of ethyl 1-(substituted benzoyl)-5-methylpyrrolo[1,2-*a*]quinoline-3-carboxylate **4a**–**f** and dimethyl 1-(substituted benzoyl)-5-methylpyrrolo[1,2-*a*]quinoline-2,3-dicarboxylate **4g**–**k** analogues tested for anti-tuberculosis (TB) activity against H37Rv strain and multidrug-resistant *Mycobacterium tuberculosis*.

Construction of the title compounds **4a**–**k** was achieved by a two-step chemical synthesis (Scheme 1). Purification was completed by column chromatography and the yield was 54–67% after purification [19,23]. The purity of the compounds was ascertained by high-performance liquid chromatography (HPLC) and was found to be >99%. The molecular structures of the title compounds used for antitubercular properties are listed in Figure 2.

### 2.2. Antitubercular Activity

We conducted resazurin microplate assay to screen the title compounds **4a**–**k** for antitubercular activity against H37Rv and multidrug-resistant (MDR) strains of *Mycobacterium tuberculosis.* Minimum inhibitory activity of the title compounds **4a**–**k** is tabulated in Table 1. All 11 compounds tested exhibited antitubercular activity against H37Rv strain, ATCC number 25,177, and only three compounds, **4f**, **4j**, and **4k**, exhibited anti-TB activity at 64, 16, and 32 µg/mL, respectively, against multidrug-resistant *Mycobacterium tuberculosis*, which was resistant to rifampicin (Rif) and isoniazid (Inh). The structure activity relationship and molecular mechanics studies of the title compounds confirmed that 3,5-substitution on the benzoyl group at the first position of the indolizine nucleus is critical for the activity.

### 2.3. Toxicity Studies

The anti-TB compounds **4f**, **4j**, and **4k** (Table 1) were evaluated in safety studies via MTT assay. Overall, the three compounds exhibited no toxicity up to 250 µg/mL across peripheral blood mononuclear cell lines. This result provides assurance about the safety of the compounds and encourages us to consider **4f**, **4j**, and **4k** as lead compounds for further refinement to achieve the most promising anti-TB agents against MDR strains of *Mycobacterium tuberculosis*.

### 2.4. Computational Studies

#### 2.4.1. Target Identification

**Figure 3:**
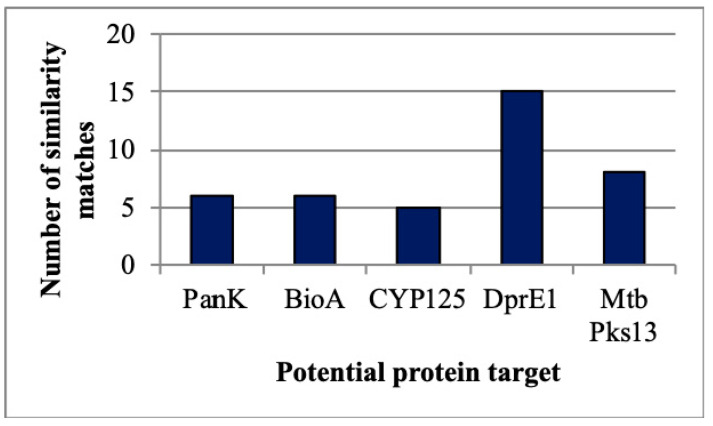
Number of similarity matches for best identified potential protein targets for synthesized compounds.

Potential protein targets that had one or more ligands with flexophore similarity value of 0.6 or higher to one or more of the synthesized compounds were identified. Among those, five proteins had five or more similarity matches between one of their co-crystal ligands and one of the synthesized compounds. Figure 3 shows a plot of these proteins and the number of ligand similarity matches. As shown, enzyme DprE1 had the most similarity matches. Five of the synthesized compounds, **4a**–**e**, displayed similarity with one or more of seven co-crystalized ligands of the DprE1 enzyme, with a total of 15 similarity matches. This enzyme has an important role in the biosynthesis of the *Mycobacterium tuberculosis* cell wall and is a potential target for developing anti-TB drugs.

#### 2.4.2. ADME Analysis

The result of ADME prediction from the SwissADME web server is presented in Table 2. For most of the compounds, the cLogP value is in the optimum range, with the exception of compounds **4b**, **4i**, and **4k**, which have cLogP values exceeding the threshold of 5. This can cause low water solubility and oral bioavailability. The number of rotatable bonds for all compounds, except compound **4k**, is in the desired range of seven, or fewer, for oral bioavailability. Prediction of gastrointestinal (GI) absorption shows high probability for most compounds; only compounds **4i** and **4k** were predicted to have low GI absorption, most likely due to the high cLogP value and number of rotatable bonds. Several compounds were predicted to have BBB permeability. All compounds except **4i** were predicted to be not potential P-glycoprotein (P-gp) substrates. Finally, the prediction of CYP isoform inhibition indicates that CYP1A2 and CYP34A might be inhibited by some compounds, such as **4a** and **4d**, while CYP2D6 was predicted to be not inhibited by any of the compounds. Overall, the compounds appear to have druglike properties, as 8 out of the 11 compounds have zero violations of the Lipinski rule of five conditions.

#### 2.4.3. Docking Studies

Docking is currently considered an essential routine calculation in any drug discovery study [25]. It gives better qualitative and quantitative insight into the binding affinity of the inhibitor at the active site of the target enzyme [26,27]. Indeed, the calculated binding free energy, inhibition constant and intramolecular interactions are the key parameters to compare the lists of inhibitors, which may be hard to achieve experimentally. In this study, 11 compounds (shown in Table 1) were docked into two proteins, PDB: Pks13 and DprE1, respectively. The docking free energy and calculated inhibition constant of the docked compounds are listed in Table 3.

The parameters listed in Table 3 reveal the high affinity of these compounds against Pks13 protein in comparison with DprE1 protein, as they showed higher binding affinity and less inhibition constant in nanomolar units. Specifically, compound **4d** showed the highest binding affinity against both the proteins, which is in agreement with the experimental results, followed by compounds **4f** and **4j** and the remaining compounds.

**Figure 4:**
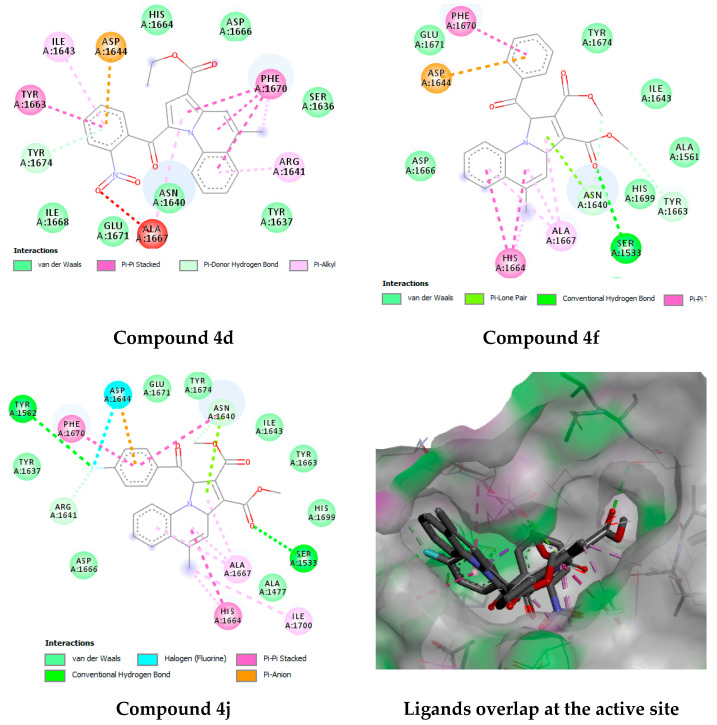
Intramolecular interactions of compounds **4d**, **4f**, and **4j** with active site of Pks13 protein and their overlap at the active site.

**Figure 5:**
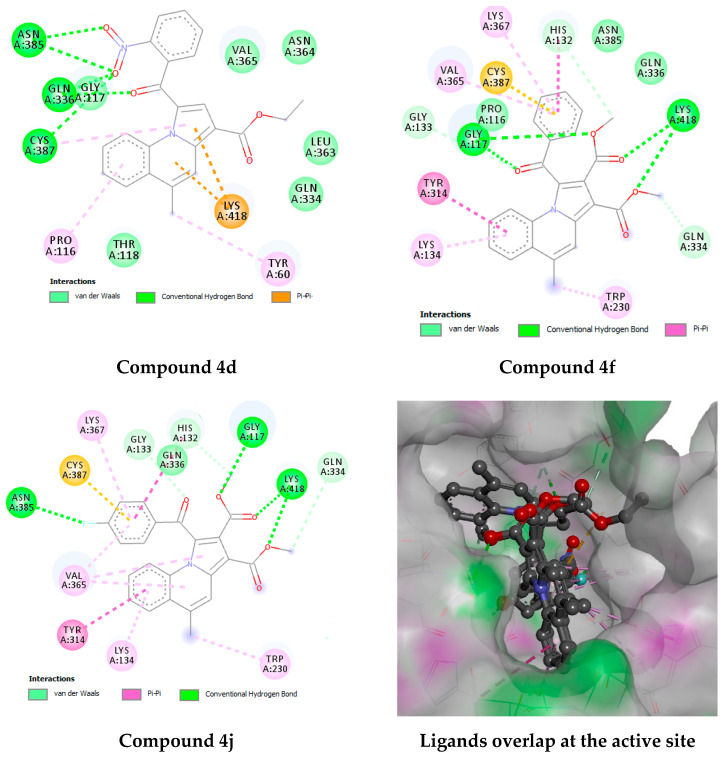
Intramolecular interactions of compounds **4d**, **4f**, and **4j** with active site of DprE1 protein and their overlap at the active site.

The high affinity of compounds **4d**, **4f**, and **4j** is obviously attributed to the strong interactions with the protein active site, as shown in Figure 4 and Figure 5. Hydrogen bonds, pi–pi interactions, and van der Waals forces are among these interactions.

## 3. Materials and Methods

### 3.1. Chemistry of Compounds

Synthesis of a series of ethyl 1-(substituted benzoyl)-5-methylpyrrolo[1,2-*a*]quinoline-3-carboxylate **4a**–**f** and dimethyl 1-(substituted benzoyl)-5-methylpyrrolo[1,2-*a*]quinoline-2,3-dicarboxylate **4g**–**k** analogues were achieved at 54–67% yield (Scheme 1 and Figure 2). The characterization of the title compounds **4a-k** is completed by instrumental techniques such as FT-IR, ^1^H-NMR, and ^13^C-NMR (Figures S1–S33 are available as supplementary with experimental details) [19,23]. The purity of the compounds was confirmed by HPLC and it was found to be more than 99%. Physicochemical characteristics of substituted pyrrolo[1,2-*a*]quinoline derivatives **4a**–**k** are available in supplementary material as Table S1.

### 3.2. Antitubercular Activity

#### 3.2.1. Resazurin Microplate Assay

Anti-TB screening of test compounds **4a**–**f** and **4g**–**k** was carried out against H37Rv and MDR strains of *Mycobacterium tuberculosis* via the resazurin microplate assay (REMA) method, as described in our previous communication [8,28]. Testing was conducted on stored MDR-MTB cultures isolated from patient sputum specimens; pure colonies from a single strain were retrieved from storage and subcultured for further testing. Clinical isolates of a well-characterized MDR strain of *Mycobacterium tuberculosis* with resistance to rifampicin (Rif) and isoniazid (Inh) were selected for testing. Mutations within the *rpoB* gene and *katG* or *inhA* gene conferred resistance to Rif and Inh, respectively.

#### 3.2.2. Determining Minimum Inhibitory Concentration

All the 11 test compounds **4a**–**f** and **4g**–**k** were assessed using the agar incorporation method, which was performed 3 times against the H37Rv and MDR-TB strains, demonstrating resistance to rifampicin (1 µg/mL) and isoniazid (0.2 µg/mL). The minimum inhibitory concentration (MIC) was determined [29]. The MTB reference strain H37Rv (American Type Culture Collection (ATCC) 25177) and MDR-TB were cultured for a total of 3 weeks in Middlebrook 7H11 medium [30] and were then supplemented with Oleic Albumin Dextrose Catalase (OADC) (0.005% *v*/*v* oleic acid, 0.2% *w*/*v* glucose, 0.085% *w*/*v* NaCl, 0.02% *v*/*v* catalase, and 0.5% 171 *w*/*v* bovine serum albumin (BSA)). Incubation was set at 37 °C. The obtained cultures were utilized to prepare an inoculum in a sterile tube with 0.05% Tween 80 and 4.5 mL of phosphate buffer with glass beads (5 mm in diameter) by vortexing. After this, the cultures settled for a total of 45 min; the clear bacterial supernatant was standardized to McFarland Number 1 using sterile water. The resulting bacterial concentration was approximately 1 × 10^7^ colony forming units (CFU)/mL, which was then diluted with sterile water. Overall, 100 µL of the dilution was added to Middlebrook 7H10 agar plates containing 8–0.125 µg/mL of the agent. The test compounds (8 µg/mL) were dissolved in distilled water and diluted to the required concentration before being added to the agar medium. The test compound MICs were read 3 weeks following 37 °C incubation and were regarded as the minimum drug concentration that could inhibit >99% growth of the bacterial culture when compared to controls. The results of this evaluation are presented in Table 1.

### 3.3. Safety Studies

The most promising anti-TB compounds **4f**, **4j**, and **4k** from the series that exhibited anti-TB activity against MDR strains of MTB were subjected to safety studies by 3-(4,5-dimethylthiazol-2-yl)-2,5-diphenyltetrazolium bromide (MTT) assay. MTT cytotoxicity assay evaluated the cytotoxic effects of test compounds **4f**, **4j**, and **4k** according to the described protocol [31].

### 3.4. Computational Studies

#### 3.4.1. Target Identification

In order to identify the possible molecular target of the synthesized compounds, we adapted ligand-based and structure-based in silico approaches. In the ligand-based approach, we searched for ligands with known targets that had high similarity to the synthesized compounds, as similar molecules are expected to bind to the same target [32,33,34]. In the first step, all ligands that are known to interact with proteins from the *Mycobacterium tuberculosis* organism were retrieved from the Protein Data Bank (PDB). The targets of these retrieved ligands are known and crystallized. In the second step, each synthesized compound was used as a reference compound in a similarity query to find compounds from the PDB-retrieved ligand data set that are similar to the reference compound. In order to measure similarity, the flexophore pharmacophore descriptor was used. The advantages of using this method are that it measures similarity in terms of the 3D-pharmacophore interaction points and takes into account the flexibility of the molecules by generating a set of favorable conformations rather than a single conformation. As the protein–ligand interactions are governed by the molecular interactions represented by the molecule’s pharmacophore, the flexophore method is suitable in this case [35]. Ligands from the PDB-retrieved ligand data set that display 0.6 or higher similarity with one or more synthesized compounds were selected for further investigation. In the next step, the protein targets of the selected ligands were examined. For each protein, the number of similarity matches was calculated as the total number of similarity queries with a value of 0.6 or higher between any of its ligands and any of the synthesized compounds. The protein with the most similar ligands was selected for further study.

#### 3.4.2. ADME Analysis

The pharmacokinetic profiles of lead compounds are of prime importance. Early ADME studies using in silico prediction can assist in deciding which lead compounds to select for further development [26,36]. In this study, we used the SwissADME web server to predict important ADME-related properties of the synthesized compounds [37]. The factors affecting oral bioavailability, such as the number of rotatable bonds and Lipinski rule of five conditions, were calculated to gain insight into the probability of oral bioavailability [38,39]. Also, the blood–brain barrier (BBB) permeability was calculated, as this property might require an adjustment in order to avoid central nervous system (CNS)-related adverse effects. The potential binding of the compounds to the efflux pump P-glycoprotein (P-gp) was predicted, as this protein is known for efflux of a wide range of molecules, including drug substances. This can lead to reduced bioavailability or resistance [40,41]. Finally, the potential inhibition of some important cytochrome P450 (CYP 450) isoforms was predicted, as inhibition of one or more of these enzymes can cause drug–drug interactions [42].

#### 3.4.3. Docking Studies

The antitubercular target compounds shown in Table 1 were selected for further theoretical investigation using docking calculations. GaussView and Gaussian09 software were used to build the starting structures of potential compounds [43]. Each structure was optimized using the molecular mechanics method, then the AM1 semi-empirical method was implemented in gaussian09 code to get the structures at their energy ground state. Two target proteins were tested theoretically; Pks13 thioesterase and decaprenylphosphoryl-Beta-d-ribose oxidase. The crystal structure of Pks13 and DprE1 were downloaded from the RCSB Protein Databank with PDB codes 5V3Y [44] and 5P8K [45], respectively. The protein structure was cleaned from water and other co-crystalized molecules. The Autodock4 program (Molinspiration database) was used to prepare the structures of the ligand compounds and the protein macromolecule and to perform the docking calculations. Polar hydrogens and Kollman united atom- type charges were added to neutralize the protein structure. Autogrid4 was used to prepare the force field parameters with grid box dimensions of 60 × 60 × 60 with point separation equal to 0.375 Å. The Lamarckian genetic algorithm, which is considered one of the best docking algorithms, was used to run 250 docking runs for the 11 inhibitors and control ligands. The docked conformations were clustered and ranked according to the binding or docking free energy (ΔG). The protein inhibitor intermolecular interactions at the active site were visualized using Discovery studio 5.0 visualizer.

## 4. Conclusions

In summary, a series of compounds **4a**–**k** with [1,2-*a*]quinoline scaffolds were tested for anti-TB activity. The test compound **4j** emerged as the most promising anti-TB compound against MDR strains of *Mycobacterium tuberculosis.* In vitro cytotoxicity evaluation of **4f**, **4j**, and **4k** confirmed the safety of the compounds up to 250 µg/mL. Prediction of ADME properties showed that most compounds, except **4f** and **4j**, have good druglike properties and potential oral bioavailability. Docking calculations were used to study the binding affinity of the target compounds and to elucidate the intermolecular interactions at the protein active site, which supported the experimental findings.

## Figures and Tables

**Table 1:** Antitubercular activity of ethyl 1-(substituted benzoyl)-5-methylpyrrolo[1,2-*a*]quinoline-3-carboxylate **4a**–**f** and dimethyl 1-(substituted benzoyl)-5-methylpyrrolo[1,2-*a*]quinoline-2,3-dicarboxylate **4g**–**k** analogues against H37Rv and multidrug-resistant (MDR)–*Mycobacterium tuberculosis* (MTB) strains of *Mycobacterium tuberculosis*.

Compound Code	MIC (µg/mL)	
	H37Rv *	MDR-MTB **
**4a**	64	NA
**4b**	32	NA
**4c**	32	NA
**4d**	8	NA
**4e**	20	NA
**4f**	16	64
**4g**	32	NA
**4h**	128	NA
**4i**	128	NA
**4j**	8	16
**4k**	32	32

* American Type Culture Collection (ATCC): 25177. ** These isolates are resistant to first-line antibiotics isoniazid (0.2 µg/mL) and rifampicin (1 µg/mL). MIC, minimum inhibitory concentration; NA, not active (concentration considered for screening was 0.2–128 µg/mL).

**Table 2:** Result of absorption, distribution, metabolism, and excretion (ADME) prediction from SwissADME web server. GI, gastrointestinal; BBB, blood–brain barrier; CYP, cytochrome P.

Compound Code	*c*Log*P*^1^	Rotatable Bonds	GI Absorption	BBB Permeable	P-gp Binding	CYP Inhibition			Lipinski Violations
						1A2	2D6	34A	
4a	4.320	5	High	Yes	No	Yes	No	Yes	0
4b	5.209	5	High	Yes	No	Yes	No	No	1
4c	4.585	5	High	Yes	No	Yes	No	No	0
4d	3.563	6	High	No	No	Yes	No	Yes	0
4e	4.484	5	High	Yes	No	Yes	No	No	0
4f	3.991	6	High	Yes	No	No	No	No	0
4g	3.827	6	High	No	No	No	No	Yes	0
4h	3.069	7	High	No	No	No	No	Yes	0
4i	6.181	7	Low	No	Yes	No	No	No	1
4j	4.092	6	High	No	No	No	No	No	0
4k	5.688	8	Low	No	No	Yes	No	No	2

^1^*c*Log*P* was calculated using DataWarrior software [24].

**Table 3:** Docking free energy (kcal/mol) and inhibition constant K_i_ of target compounds **4a**–**k**.

Comp. Code	Experimental MIC (µg/mL)	Docking Free Energy	Experimental MIC (µg/mL)	Docking Free Energy
	H37Rv *	Pks13	MDR-MTB	DprE1
**4a**	64	−10.91 (10.05 nM)	NA	−7.89 (1.63 µM)
**4b**	32	−9.73 (73.15 nM)	NA	−8.10 (1.15 µM)
**4c**	32	−11.18 (6.35 nM)	NA	−7.88 (1.67 µM)
**4d**	8	−11.40 (4.38 nM)	NA	−9.38 (133.64 nM)
**4e**	20	−9.44 (119.83 nM)	NA	−7.76 (2.05 µM)
**4f**	16	−9.16 (191.47 nM)	64	−8.33 (782.97 nM)
**4g**	32	−10.40(23.84 nM)	NA	−7.33 (4.22 µM)
**4h**	128	−10.43 (22.68 nM)	NA	−7.30 (4.48 µM)
**4i**	128	−9.39 (131.39 nM)	NA	−7.13 (5.96 µM)
**4j**	8	−9.54 (101.68 nM)	16	−8.31 (810.31 nM)
**4k**	32	−9.10 (212.01 nM)	32	−7.19 (5.40 µM)

* American Type Culture Collection (ATCC): 25177.

## Supplementary Materials

The following are available online at https://www.mdpi.com/2079-6382/9/5/233/s1, Figure S1: FT-IR of ethyl-1-(4-cyanobenzoyl)-5-methylpyrrolo[1,2-a]quinoline-3-carboxylate (**4a**); Figure S2: ^1^H-NMR of ethyl-1-(4-cyanobenzoyl)-5-methylpyrrolo[1,2-*a*]quinoline-3-carboxylate (**4a**); Figure S3: ^13^C-NMR of ethyl-1-(4-cyanobenzoyl)-5-methylpyrrolo[1,2-*a*]quinoline-3-carboxylate (**4a**); Figure S4: FT-IR of ethyl-1-(4-bromobenzoyl)-5-methylpyrrolo[1,2-*a*]quinoline-3-carboxylate (**4b**); Figure S5: ^1^H-NMR of ethyl-1-(4-bromobenzoyl)-5-methylpyrrolo[1,2-*a*]quinoline-3-carboxylate (**4b**); Figure S6: ^13^C-NMR of ethyl-1-(4-bromobenzoyl)-5-methylpyrrolo[1,2-*a*]quinoline-3-carboxylate (**4b**); Figure S7: FT-IR of ethyl-1-(4-fluorobenzoyl)-5-methylpyrrolo[1,2-*a*]quinoline-3-carboxylate (**4c**); Figure S8: ^1^H-NMR of ethyl-1-(4-fluorobenzoyl)-5-methylpyrrolo[1,2-*a*]quinoline-3-carboxylate (**4c**); Figure S9: ^13^C-NMR of ethyl-1-(4-fluorobenzoyl)-5-methylpyrrolo[1,2-*a*]quinoline-3-carboxylate (**4c**); Figure S10: FT-IR of ethyl-5-methyl-1-(2-nitrobenzoyl)pyrrolo[1,2-*a*]quinoline-3-carboxylate (**4d**); Figure S11: ^1^H-NMR of ethyl-5-methyl-1-(2-nitrobenzoyl)pyrrolo[1,2-*a*]quinoline-3-carboxylate (**4d**); Figure S12: ^13^C-NMR of ethyl-5-methyl-1-(2-nitrobenzoyl)pyrrolo[1,2-*a*]quinoline-3-carboxylate (**4d**); Figure S13: FT-IR of ethyl-1-benzoyl-5-methylpyrrolo[1,2-*a*]quinoline-3-carboxylate (**4e**); Figure S14: ^1^H-NMR of ethyl-1-benzoyl-5-methylpyrrolo[1,2-*a*]quinoline-3-carboxylate (**4e**); Figure S15: ^13^C-NMR of ethyl-1-benzoyl-5-methylpyrrolo[1,2-*a*]quinoline-3-carboxylate (**4e**); Figure S16: FT-IR of dimethyl-1-benzoyl-5-methylpyrrolo[1,2-*a*]quinoline-2,3-dicarboxylate (**4f**); Figure S17: ^1^H-NMR of dimethyl-1-benzoyl-5-methylpyrrolo[1,2-*a*]quinoline-2,3-dicarboxylate (**4f**); Figure S18: ^13^C-NMR of dimethyl-1-benzoyl-5-methylpyrrolo[1,2-*a*]quinoline-2,3-dicarboxylate (**4f**); Figure S19: FT-IR of dimethyl-1-(4-cyanobenzoyl)-5-methylpyrrolo[1,2-*a*]quinoline-2,3-dicarboxylate (**4g**); Figure S20: ^1^H-NMR of dimethyl-1-(4-cyanobenzoyl)-5-methylpyrrolo[1,2-*a*]quinoline-2,3-dicarboxylate (**4g**); Figure S21: ^13^C-NMR of dimethyl-1-(4-cyanobenzoyl)-5-methylpyrrolo[1,2-*a*]quinoline-2,3-dicarboxylate (**4g**); Figure S22: FT-IR of dimethyl-5-methyl-1-(2-nitrobenzoyl)pyrrolo[1,2-*a*]quinoline-2,3-dicarboxylate (**4h**); Figure S23: ^1^H-NMR of dimethyl-5-methyl-1-(2-nitrobenzoyl)pyrrolo[1,2-*a*]quinoline-2,3-dicarboxylate (**4h**); Figure S24: ^13^C-NMR of dimethyl-5-methyl-1-(2-nitrobenzoyl)pyrrolo[1,2-*a*]quinoline-2,3-dicarboxylate (**4h**); Figure S25: FT-IR of ethyl-1-(3,5-bis(trifluoromethyl)benzoyl)-5-methylpyrrolo[1,2-*a*]quinoline-3-carboxylate (**4i**); Figure S26: ^1^H-NMR of ethyl-1-(3,5-bis(trifluoromethyl)benzoyl)-5-methylpyrrolo[1,2-*a*]quinoline-3-carboxylate (**4i**); Figure S27: ^13^C-NMR of ethyl-1-(3,5-bis(trifluoromethyl)benzoyl)-5-methylpyrrolo[1,2-*a*]quinoline-3-carboxylate (**4i**); Figure S28: FT-IR of dimethyl-1-(4-fluorobenzoyl)-5-methylpyrrolo[1,2-*a*]quinoline-2,3-dicarboxylate (**4j**); Figure S29: ^1^H-NMR of dimethyl-1-(4-fluorobenzoyl)-5-methylpyrrolo[1,2-*a*]quinoline-2,3-dicarboxylate (**4j**); Figure S30: ^13^C-NMR of dimethyl-1-(4-fluorobenzoyl)-5-methylpyrrolo[1,2-*a*]quinoline-2,3-dicarboxylate (**4j**); Figure S31: FT-IR of dimethyl-1-(3,5-bis(trifluoromethyl)benzoyl)-5-methylpyrrolo[1,2-*a*]quinoline-2,3-dicarboxylate (**4k**); Figure S32: ^1^H-NMR of dimethyl-1-(3,5-bis(trifluoromethyl)benzoyl)-5-methylpyrrolo[1,2-*a*]quinoline-2,3-dicarboxylate (**4k**); Figure S33: ^13^C-NMR of dimethyl-1-(3,5-bis(trifluoromethyl)benzoyl)-5-methylpyrrolo[1,2-*a*]quinoline-2,3-dicarboxylate (**4k**); Table S1: Physicochemical characteristics of substituted pyrrolo[1,2-*a*]quinoline derivatives **4a**–**k**.

## References

[1] Shruthi T., Eswaran S., Shivarudraiah P., Narayanan S., Subramanian S. (2019). Synthesis, antituberculosis studies and biological evaluation of new quinoline derivatives carrying 1, 2, 4-oxadiazole moiety. Bioorg. Med. Chem. Lett..

[2] Ambre P.K., Pissurlenkar R.R.S., Wavhale R.D., Shaikh M.S., Khedkar V.M., Wan B., Franzblau S.G., Coutinho E.C. (2013). Design, synthesis, and evaluation of 4-(substituted)phenyl-2-thioxo-3,4-dihydro-1*H*-chromino[4,3-d]pyrimidin-5-one and 4-(substituted)phenyl-3,4-dihydro-1*H*-chromino[4,3-d]pyrimidine-2,5-dione analogs as antitubercular agents. Med. Chem. Res..

[3] Caminero J.A., Sotgiu G., Zumla A., Migliori G.B. (2010). Best drug treatment for multidrug-resistant and extensively drug-resistant tuberculosis. Lancet Infect. Dis..

[4] Espinal M.A. (2003). The global situation of MDR-TB. Tuberculosis.

[5] Alveera S., Venugopala K.N., Khedr M.A., Pillay M., Nwaeze K.U., Coovadia Y., Shode F., Odhav B. (2017). Antimycobacterial, docking and molecular dynamic studies of pentacyclic triterpenes from *Buddleja saligna* leaves. J. Biomol. Struct. Dyn..

[6] Venugopala K.N., Albericio F., Coovadia Y.M., Kruger H.G., Maguire G.E.M., Pillay M., Govender T. (2011). Total synthesis of a depsidomycin analogue by convergent solid-phase peptide synthesis and macrolactonization strategy for antitubercular activity. J. Pept. Sci..

[7] Venugopala K.N., Nayak S.K., Pillay M., Prasanna R., Coovadia Y.M., Odhav B. (2013). Synthesis and antitubercular activity of 2-(substituted phenyl/benzyl-amino)-6-(4-chlorophenyl)-5-(methoxycarbonyl)-4-methyl-3,6-dihydropyrimidin-1-ium chlorides. Chem. Biol. Drug Des..

[8] Venugopala K.N., Dharma Rao G.B., Bhandary S., Pillay M., Chopra D., Aldhubiab B.E., Attimarad M., Alwassil O.I., Harsha S., Mlisana K. (2016). Design, synthesis, and characterization of (1-(4-aryl)-1*H*-1,2,3-triazol-4-yl)methyl, substituted phenyl-6-methyl-2-oxo-1,2,3,4-tetrahydropyrimidine-5-carboxylates against *Mycobacterium tuberculosis*. Drug Des. Dev. Ther..

[9] Venugopala K.N., Chandrashekharappa S., Pillay M., Bhandary S., Kandeel M., Mahomoodally F.M., Morsy M.A., Chopra D., Aldhubiab B.E., Attimarad M. (2019). Synthesis and structural elucidation of novel benzothiazole derivatives as anti-tubercular agents: In-silico screening for possible target identification. Med. Chem..

[10] Venugopala K.N., Chandrashekharappa S., Pillay M., Abdallah H.H., Mahomoodally F.M., Bhandary S., Chopra D., Attimarad M., Aldhubiab B.E., Nair A.B. (2019). Computational, crystallographic studies, cytotoxicity and anti-tubercular activity of substituted 7-methoxy-indolizine analogues. PLoS ONE.

[11] Venugopala K.N., Tratrat C., Pillay M., Mahomoodally F.M., Bhandary S., Chopra D., Morsy M.A., Haroun M., Aldhubiab B.E., Attimarad M. (2019). Anti-tubercular activity of substituted 7-methyl and 7-formylindolizines and in silico study for prospective molecular target identification. Antibiotics.

[12] Khedr M.A., Pillay M., Chandrashekharappa S., Chopra D., Aldhubiab B.E., Attimarad M., Alwassil O.I., Mlisana K., Odhav B., Venugopala K.N. (2018). Molecular modeling studies and anti-TB activity of trisubstituted indolizine analogues; molecular docking and dynamic inputs. J. Biomol. Struct. Dyn..

[13] Venugopala K.N., Tratrat C., Pillay M., Chandrashekharappa S., Al-Attraqchi O.H.A., Aldhubiab B.E., Attimarad M., Alwassil O.I., Nair A.B., Sreeharsha N. (2020). In silico design and synthesis of tetrahydropyrimidinones and tetrahydropyrimidinethiones as potential thymidylate kinase inhibitors exerting anti-TB activity against *Mycobacterium tuberculosis*. Drug Des. Dev. Ther..

[14] Liu Y., Zhang Y., Shen Y.-M., Hu H.-W., Xu J.-H. (2010). Regioselective synthesis of 3-acylindolizines and benzo- analogues via 1,3-dipolar cycloadditions of N-ylides with maleic anhydride. Org. Biomol. Chem..

[15] Sandeep C., Venugopala K.N., Mohammed A.K., Mahesh A., Basavaraj P., Rashmi S.K., Rashmi V., Odhav B. (2016). Review on chemistry of natural and synthetic indolizines with their chemical and pharmacological properties. J. Basic Clin. Pharm..

[16] Uppar V., Mudnakudu-Nagaraju K.K., Basarikattia A.I., Chougala M., Chandrashekharappa S., Mohan M.K., Banuprakashe G., Venugopala K.N., Ningegowda R., Padmashali B. (2020). Microwave induced synthesis, and pharmacological properties of novel 1- benzoyl-4-bromopyrrolo[1,2-*a*]quinoline-3-carboxylate analogues. Chem. Data Collect..

[17] Dillard R.D., Pavey D.E., Benslay D.N. (1973). Synthesis and anti-inflammatory activity of some 2,2-dimethyl-1,2-dihydroquinolines. J. Med. Chem..

[18] Dubé D., Blouin M., Brideau C., Chan C.-C., Desmarais S., Ethier D., Falgueyret J.-P., Friesen R.W., Girard M., Girard Y. (1998). Quinolines as potent 5-lipoxygenase inhibitors: Synthesis and biological profile of L-746,530. Bioorg. Med. Chem. Lett..

[19] Uppar V., Chandrashekharappa S., Venugopala K.N., Deb P.K., Kar S., Alwassil O.I., Gleiser R.M., Garcia D., Odhav B., Mohan M.K. (2020). Synthesis and characterization of pyrrolo[1,2-*a*]quinoline derivatives for their larvicidal activity against *Anopheles arabiensis*. Struct. Chem..

[20] Sechi M., Rizzi G., Bacchi A., Carcelli M., Rogolino D., Pala N., Sanchez T.W., Taheri L., Dayam R., Neamati N. (2009). Design and synthesis of novel dihydroquinoline-3-carboxylic acids as HIV-1 integrase inhibitors. Bioorg. Med. Chem..

[21] Martins C., Carreiras M.C., Leon R., de los Rios C., Bartolini M., Andrisano V., Iriepa I., Moraleda I., Galvez E., Garcia M. (2011). Synthesis and biological assessment of diversely substituted furo[2,3-b]quinolin-4-amine and pyrrolo[2,3-*b*]quinolin-4-amine derivatives, as novel tacrine analogues. Eur. J. Med. Chem..

[22] Cox E., Laessig K. (2014). FDA Approval of Bedaquiline—The benefit–risk balance for drug-resistant Tuberculosis. N. Engl. J. Med..

[23] Hu H., Feng J., Zhu Y., Gu N., Kan Y. (2012). Copper acetate monohydrate: A cheap but efficient oxidant for synthesizing multi-substituted indolizines from pyridinium ylides and electron deficient alkenes. RSC Adv..

[24] Sander T., Freyss J., von Korff M., Rufener C. (2015). DataWarrior: An open-source program for chemistry aware data visualization and analysis. J. Chem. Inf. Model..

[25] Deb P.K., Mailavaram R., Chandrasekaran B., Kaki V.R., Kaur R., Kachler S., Klotz K.-N., Akkinepally R.R. (2018). Synthesis, adenosine receptor binding and molecular modelling studies of novel thieno[2,3-d]pyrimidine derivatives. Chem. Biol. Drug Des..

[26] Deb P.K., Ahmad J., Dina E.-R., Tan Y.H., Nasr E.M., Pichika M.R. (2014). Molecular docking studies and comparative binding mode analysis of FDA approved HIV protease inhibitors. Asian J. Chem..

[27] Deb P.K., El-Rabie D., Ahmad J., Nalaiya J.A.P., Siong L.C., Kulasekar A.L.K., Pichika M.R. (2014). In silico binding mode analysis (molecular docking studies), and ADME prediction of some novel inhibitors of aurora kinase a in clinical trials. Asian J. Chem..

[28] Martin A., Morcillo N., Lemus D., Montoro E., Telles M.A., Simboli N., Pontino M., Porras T., Leon C., Velasco M. (2005). Multicenter study of MTT and resazurin assays for testing susceptibility to first-line anti-tuberculosis drugs. Int. J. Tuberc. Lung Dis..

[29] Yoshikuni O., Mayumi T., Kenichi S. (2001). Inhibitory activity of quinolones against DNA gyrase of *Mycobacterium tuberculosis*. J. Antimicrob. Chemother..

[30] Middlebrook G., Reggiards Z., Tigertt W.D. (1977). Automable radiometric detection of growth of *Mycobacterium tuberculosis* in selective media. Am. Rev. Respir. Dis..

[31] Mossman T. (1983). Rapid colorimetric assay for cellular growth and survival: Application to proliferation and cytotoxicity assays. J. Immunol. Methods.

[32] Rollinger J.M., Schuster D., Danzl B., Schwaiger S., Markt P., Schmidtke M., Gertsch J., Raduner S., Wolber G., Langer T. (2009). In silico target fishing for rationalized ligand discovery exemplified on constituents of Ruta graveolens. Planta Med..

[33] Jenkins J.L., Bender A., Davies J.W. (2006). In silico target fishing: Predicting biological targets from chemical structure. Drug Discov. Today Technol..

[34] Liu X., Xu Y., Li S., Wang Y., Peng J., Luo C., Luo X., Zheng M., Chen K., Jiang H. (2014). In Silico target fishing: Addressing a “Big Data” problem by ligand-based similarity rankings with data fusion. J. Cheminformatics.

[35] Von Korff M., Freyss J., Sander T. (2008). Flexophore, a new versatile 3D pharmacophore descriptor that considers molecular flexibility. J. Chem. Inf. Model..

[36] Deb P.K., Al-Attraqchi O., Al-Qattan M.N., Raghu Prasad M., Tekade R.K., Tekade R.K. (2018). Chapter 19—Applications of Computers in Pharmaceutical Product Formulation. Dosage Form Design Parameters.

[37] Daina A., Michielin O., Zoete V. (2017). SwissADME: A free web tool to evaluate pharmacokinetics, drug-likeness and medicinal chemistry friendliness of small molecules. Sci. Rep..

[38] Veber D.F., Johnson S.R., Cheng H.-Y., Smith B.R., Ward K.W., Kopple K.D. (2002). Molecular properties that influence the oral bioavailability of drug candidates. J. Med. Chem..

[39] Pollastri M.P. (2010). Overview on the Rule of Five. Curr. Protoc. Pharmacol..

[40] Yu D.K. (1999). The contribution of P-glycoprotein to pharmacokinetic drug-drug interactions. J. Clin. Pharmacol..

[41] Fromm M. (2003). Importance of P-glycoprotein for drug disposition in humans. Eur. J. Clin. Investig..

[42] Lin J.H. (2006). CYP induction-mediated drug interactions: In vitro assessment and clinical implications. Pharm. Res..

[43] Frisch M.J., Trucks G.W.T., Schlegel H.B., Scuseria G.E., Robb M.A., Cheeseman J.R., Scalmani G., Barone V., Petersson G.A., Nakatsuji H. (2016). Gaussian 09.

[44] Aggarwal A., Parai M.K., Shetty N., Wallis D., Woolhiser L., Hastings C., Dutta N.K., Galaviz S., Dhakal R.C., Shrestha R. (2017). Development of a Novel Lead that Targets M. tuberculosis Polyketide Synthase 13. Cell.

[45] Schiebel J., Krimmer S.G., Rower K., Knorlein A., Wang X., Park A.Y., Stieler M., Ehrmann F.R., Fu K., Radeva N. (2016). High-Throughput Crystallography: Reliable and Efficient Identification of Fragment Hits. Structure.

