# Supplementary material for: Cytotoxicity and Antimycobacterial Properties of Pyrrolo[1,2-a]quinoline Derivatives: Molecular Target Identification and Molecular Docking Studies
Source: Antibiotics (Basel). 2020 May 7;9(5):233. doi: 10.3390/antibiotics9050233 (PMC7277568; doi:10.3390/antibiotics9050233)
Supplement: Supplementary file 1 [file antibiotics-09-00233-s001.pdf]

Cytotoxicity and Antimycobacterial Properties of Pyrrolo[1,2-*a*]quinoline Derivatives: Molecular Target Identification and Molecular Docking Studies

Katharigatta N. Venugopala [1,2,*], Vijayakumar Uppar [3,†], Sandeep Chandrashekharappa [4,†], Hassan H. Abdallah [5], Melendhran Pillay [6], Pran Kishore Deb [7], Mohamed A. Morsy [1,8], Bandar E. Aldhubiab [1], Mahesh Attimarad [1], Anroop B. Nair [1], Nagaraja Sreeharsha [1], Christophe Tratrat [1], Abdulmuttaleb Yousef Jaber [7], Rashmi Venugopala [9], Raghu Prasad Mailavaram [10], Bilal A. Al-Jaidi [11], Mahmoud Kandeel [12,13], Michelyne Haroun [1] and Basavaraj Padmashali [3,*]

[1] Department of Pharmaceutical Sciences, College of Clinical Pharmacy, King Faisal University, Al-Ahsa 31982, Saudi Arabia; momorsy@kfu.edu.sa (M.A.M.) baldhubiab@kfu.edu.sa (B.E.A.); mattimarad@kfu.edu.sa (M.A.); anair@kfu.edu.sa (A.B.N.); sharsha@kfu.edu.sa (N.S.); ctratrat@kfu.edu.sa (C.T.); mharoun@kfu.edu.sa (M.H.)
[2] Department of Biotechnology and Food Technology, Durban University of Technology, Durban 4001, South Africa
[3] Department of Chemistry, School of Basic Science, Rani Channamma University, Belagavi 591156, India; vijay.uppar@gmail.com (V.U.); basavarajpadmashali@yahoo.com (B.P.)
[4] Institute for Stem Cell Biology and Regenerative Medicine, NCBS, TIFR, GKVK, Bellary Road, Bangalore 560065, India; sandeepc@instem.res.in
[5] Chemistry Department, College of Education, Salahaddin University, Erbil 44001, Iraq; Hassan.Abdullah@su.edu.krd
[6] Department of Microbiology, National Health Laboratory Services, KZN Academic Complex, Inkosi Albert Luthuli Central Hospital, Durban 4001, South Africa; melendhra.pillay@nhls.ac.za
[7] Faculty of Pharmacy, Philadelphia University, Amman 19392, Jordan; prankishore1@gmail.com (P.K.D.); AJaber@philadelphia.edu.jo (AYJ)
[8] Department of Pharmacology, Faculty of Medicine, Minia University, El-Minia 61511, Egypt
[9] Department of Public Health Medicine, University of KwaZulu-Natal, Howard College Campus, Durban 4001, South Africa; rashmivenugopala@gmail.com
[10] Department of Pharmaceutical Chemistry, Shri Vishnu College of Pharmacy, Vishnupur, Bhimavaram 534 202, West Godavari Dist., Andhra Pradesh, India; raghumrp@svcp.edu.in
[11] Faculty of Pharmacy, Yarmouk University, Irbid 21163, Jordan; bilaljeaidi77@gmail.com
[12] Department of Biomedical Sciences, College of Veterinary Medicine, King Faisal University, Al-Ahsa 31982, Kingdom of Saudi Arabia; mkandeel@kfu.edu.sa
[13] Department of Pharmacology, Faculty of Veterinary Medicine, Kafrelsheikh University, Kafrelsheikh 33516, Egypt
* Correspondence: kvenugopala@kfu.edu.sa (K.N.V.); basavarajpadmashali@yahoo.com (B.P.); Tel.: +966-1358-98842 (K.N.V.); +91-98-4421-8894 (B.P.)
† Contributed equally.

TABLE OF CONTENTS

Sl No	Description	Page numbers
1	General synthetic procedure for the preparation of 1-(2-(substitutedphenyl)-2-oxoethyl)-4-methylquinolin-1-ium bromide **(3a-f)**	4
2	General procedure for the preparation of ethyl/methyl-1-(substitutedbenzoyl)-5-methylpyrrolo[1,2-a]quinoline-3-carboxylates **(4a-k)**	5
3	Table S1: Physicochemical characteristics of substituted pyrrolo[1,2-*a*]quinoline derivatives **4a-k**.	9
4	Figure S1: FT-IR of ethyl-1-(4-cyanobenzoyl)-5-methylpyrrolo[1,2-*a*]quinoline-3-carboxylate (**4a**)	10
5	Figure S2: ^{1}H-NMR of ethyl-1-(4-cyanobenzoyl)-5-methylpyrrolo[1,2-*a*]quinoline-3-carboxylate (**4a**)	11
6	Figure S3: ^{13}C-NMR of ethyl-1-(4-cyanobenzoyl)-5-methylpyrrolo[1,2-*a*]quinoline-3-carboxylate (**4a**)	12
7	Figure S4: FT-IR of ethyl-1-(4-bromobenzoyl)-5-methylpyrrolo[1,2-*a*]quinoline-3-carboxylate (**4b**)	13
8	Figure S5: ^{1}H-NMR of ethyl-1-(4-bromobenzoyl)-5-methylpyrrolo[1,2-*a*]quinoline-3-carboxylate (**4b**)	14
9	Figure S6: ^{13}C-NMR of ethyl-1-(4-bromobenzoyl)-5-methylpyrrolo[1,2-*a*]quinoline-3-carboxylate (**4b**)	15
10	Figure S7: FT-IR of ethyl-1-(4-fluorobenzoyl)-5-methylpyrrolo[1,2-*a*]quinoline-3-carboxylate (**4c**)	16
11	Figure S8: ^{1}H-NMR of ethyl-1-(4-fluorobenzoyl)-5-methylpyrrolo[1,2-*a*]quinoline-3-carboxylate (**4c**)	17
12	Figure S9: ^{13}C-NMR of ethyl-1-(4-fluorobenzoyl)-5-methylpyrrolo[1,2-*a*]quinoline-3-carboxylate (**4c**)	18
13	Figure S10: FT-IR of ethyl-5-methyl-1-(2-nitrobenzoyl)pyrrolo[1,2-*a*]quinoline-3-carboxylate (**4d**)	19
14	Figure S11: ^{1}H-NMR of ethyl-5-methyl-1-(2-nitrobenzoyl)pyrrolo[1,2-*a*]quinoline-3-carboxylate (**4d**)	20
15	Figure S12: ^{13}C-NMR of ethyl-5-methyl-1-(2-nitrobenzoyl)pyrrolo[1,2-*a*]quinoline-3-carboxylate (**4d)**	21
16	Figure S13: FT-IR of ethyl-1-benzoyl-5-methylpyrrolo[1,2-a]quinoline-3-carboxylate (**4e**)	22
17	Figure S14: ^{1}H-NMR of ethyl-1-benzoyl-5-methylpyrrolo[1,2-*a*]quinoline-3-carboxylate (**4e**)	23
18	Figure S15: ^{13}C-NMR of ethyl-1-benzoyl-5-methylpyrrolo[1,2-*a*]quinoline-3-carboxylate (**4e**)	24
19	Figure S16: FT-IR of dimethyl-1-benzoyl-5-methylpyrrolo[1,2-*a*]quinoline-2,3-dicarboxylate (**4f**)	25
20	Figure S17: ^{1}H-NMR of dimethyl-1-benzoyl-5-methylpyrrolo[1,2-*a*]quinoline-2,3-dicarboxylate (**4f**)	26
21	Figure S18: ^{13}C-NMR of dimethyl-1-benzoyl-5-methylpyrrolo[1,2-*a*]quinoline-2,3-dicarboxylate (**4f**)	27

22	Figure S19: FT-IR of dimethyl-1-(4-cyanobenzoyl)-5-methylpyrrolo[1,2-*a*]quinoline-2,3-dicarboxylate (**4g**)	28
23	Figure S20: ^{1}H-NMR of dimethyl-1-(4-cyanobenzoyl)-5-methylpyrrolo[1,2-*a*]quinoline-2,3-dicarboxylate (**4g**)	29
24	Figure S21: ^{13}C-NMR of dimethyl-1-(4-cyanobenzoyl)-5-methylpyrrolo[1,2-*a*]quinoline-2,3-dicarboxylate (**4g**)	30
25	Figure S22: FT-IR of dimethyl-5-methyl-1-(2-nitrobenzoyl)pyrrolo[1,2-*a*]quinoline-2,3-dicarboxylate (**4h**)	31
26	Figure S23: ^{1}H-NMR of dimethyl-5-methyl-1-(2-nitrobenzoyl)pyrrolo[1,2-*a*]quinoline-2,3-dicarboxylate (**4h**)	32
27	Figure S24: ^{13}C-NMR of dimethyl-5-methyl-1-(2-nitrobenzoyl)pyrrolo[1,2-*a*]quinoline-2,3-dicarboxylate (**4h**)	33
28	Figure S25: FT-IR of ethyl-1-(3,5-bis(trifluoromethyl)benzoyl)-5-methylpyrrolo[1,2-*a*]quinoline-3-carboxylate (**4i**)	34
29	Figure S26: ^{1}H-NMR of ethyl-1-(3,5-bis(trifluoromethyl)benzoyl)-5-methylpyrrolo[1,2-*a*]quinoline-3-carboxylate (**4i**)	35
30	Figure S27: ^{13}C-NMR of ethyl-1-(3,5-bis(trifluoromethyl)benzoyl)-5-methylpyrrolo[1,2-*a*]quinoline-3-carboxylate (**4i**)	36
31	Figure S28: FT-IR of dimethyl-1-(4-fluorobenzoyl)-5-methylpyrrolo[1,2-*a*]quinoline-2,3-dicarboxylate (**4j**)	37
32	Figure S29: ^{1}H-NMR of dimethyl-1-(4-fluorobenzoyl)-5-methylpyrrolo[1,2-*a*]quinoline-2,3-dicarboxylate (**4j**)	38
33	Figure S30: ^{13}C-NMR of dimethyl-1-(4-fluorobenzoyl)-5-methylpyrrolo[1,2-*a*]quinoline-2,3-dicarboxylate (**4j**)	39
34	Figure S31: FT-IR of dimethyl-1-(3,5-bis(trifluoromethyl)benzoyl)-5-methylpyrrolo[1,2-*a*]quinoline-2,3-dicarboxylate (**4k**)	40
35	Figure S32: ^{1}H-NMR of dimethyl-1-(3,5-bis(trifluoromethyl)benzoyl)-5-methylpyrrolo[1,2-*a*]quinoline-2,3-dicarboxylate (**4k**)	41
36	Figure S33: ^{13}C-NMR of dimethyl-1-(3,5-bis(trifluoromethyl)benzoyl)-5-methylpyrrolo[1,2-*a*]quinoline-2,3-dicarboxylate (**4k**)	42
37	References	43

1 General synthetic procedure for the preparation of 1-(2-(substitutedphenyl)-2-oxoethyl)-4-methylquinolin-1-ium bromides (3a-f)

To a stirred solution of 4-methyl quinoline (0.00698 mol) in dry acetone (5 mL), substitutedphenacyl bromide (0.00698 mol) was added and stirred at room temperature for 30 minutes. The formed solid was filtered out and dried under a vacuum to afford the salts 1-(2-(substituted phenyl)-2-oxoethyl)-4-methylquinolin-1-ium bromides at 88.2-99.6% yield. The characterization details of **3a-f** are described as below.

1.1 1-(2-(4-Cyanophenyl)-2-oxoethyl)-4-methylquinolin-1-ium bromide (3a)

Appearance; white colour; m.p = 123-124°C; yield = 97.6 %; ^{1}H-NMR (600MHz, DMSO-*d*$_6$); δ = 9.31-9.30 (1H, d; *J* = 6.6Hz, H$_2$), 8.61-8.59 (1H, m, H$_8$), 8.47-8.46 (1H, m, H$_5$), 8.29-8.28 (2H, m, ArH$_2$, ArH$_6$), 8.21-8.18 (4H, m, ArH$_3$, ArH$_5$ H$_7$, H$_6$), 8.08-8.05 (1H, m, H$_3$), 6.95 (2H, s, CH$_2$), 3.09 (3H, s, CH$_3$); ^{13}C-NMR (150MHz, DMSO-*d*$_6$); 190.9 (CO), 168.6, 150.0, 138.4, 137.4, 135.8, 133.4, 129.6, 129.1, 128.2, 127.5, 125.0, 123.2, 122.5, 120.0, 118.4, 116.8, 79.6, 63.4(CH$_2$), 20.4(CH$_3$); MS (ESI, Positive): *m/z* = 287.2 (M+H)$^+$; analytical calculated for C$_{19}$H$_{14b}$rN$_2$O; C, 62.14, H, 4.12, N, 7.63; found; C, 62.12, H, 4.10, N, 7.61.

1.2 1-[2-(4-Bromophenyl)-2-oxoethyl]-4-methylquinolin-1-ium bromide (3b)

Appearance; white colour; m.p = 109-110 °C; yield = 96.6 %; ^{1}H-NMR (600MHz, DMSO-*d*$_6$); δ = 9.31-9.30 (1H, d; *J* = 6.6Hz, H$_2$), 8.60-8.59 (1H, m, H$_8$), 8.42-8.41 (1H, d, H$_5$), 8.20-8.18 (2H, m, H$_6$, H$_7$), 8.07-8.05 (3H, m, ArH$_2$, ArH$_6$, ArH$_3$), 7.94-7.93 (2H, m, ArH$_3$, ArH$_5$), 6.90 (2H, s, CH$_2$), 3.09 (3H, s, CH$_3$); ^{13}C-NMR (150MHz, DMSO-*d*$_6$); 190.7 (CO), 160.6, 150.0, 138.4, 135.8, 133.2, 132.6, 130.9, 130.1, 129.4, 129.1, 127.5, 123.2, 120.0, 79.6, 63.2(CH$_2$), 20.4(CH$_3$); MS (ESI, Positive): m/z = 340.2 (M+H)$^+$and *m/z* = 342.2 (M+2H)$^+$; analytical calculated for C$_{18}$H$_{14b}$r$_2$NO; C, 51.34, H, 3.59, N, 3.33; found; C, 51.33, H, 3.56, N, 3.31

1.3 1-[2-(2-Nitrophenyl)-2-oxoethyl]-4-methylquinolin-1-ium bromide (3c)

Appearance; yellow colour; m.p = 134-135 °C; yield = 96.1 %;^{1}H-NMR (600MHz, DMSO-*d*$_6$); δ = 9.44-9.43 (1H, d; *J* = 6.6Hz, H$_2$), 8.63-8.62 (1H, m, H$_8$), 8.56-8.54 (1H, d; *J* = 9.2Hz, H$_5$), 8.33-8.27 (4H, m, H$_6$, H$_7$, H$_3$, ArH$_3$), 8.11-8.07 (2H, m, ArH$_4$, ArH$_6$), 7.99-7.95 (1H, m, ArH$_5$), 6.88 (2H, s, CH$_2$), 3.10 (3H, s, CH$_3$); ^{13}C-NMR (150MHz, DMSO-*d*$_6$); 193.3 (CO), 161.1, 150.0, 146.6, 138.2, 135.8, 134.8, 133.7, 131.8, 130.2, 129.7, 129.2, 127.6, 125.1, 123.3, 119.7, 79.6, 64.2(CH$_2$), 20.5(CH$_3$); MS (ESI, Positive): *m/z* = 307.2 (M+H)$^+$; analytical calculated for C$_{18}$H$_{14b}$rN$_2$O$_3$; C, 55.83, H, 3.90, N, 7.23; found; C, 55.79, H, 3.88, N, 7.19

1.4 1-(2-(3,5-Bis(trifluoromethyl)phenyl)-2-oxoethyl)-4-methylquinolin-1-ium bromide (3d)

Appearance; yellow colour; m.p = 120-123 °C; yield = 90.6 %;^{1}H-NMR (600MHz, DMSO-*d*$_6$); δ = 9.25-9.24 (1H, d; *J* = 6.6Hz, H$_2$), 8.70 (2H, m, H$_8$, H$_5$), 8.63-8.61(2H, m, H$_6$, H$_7$), 8.51-8.49 (1H, m, ArH$_4$), 8.23-8.20 (2H, m, ArH$_2$, ArH$_5$), 8.09-8.07 (1H, m, ArH$_3$), 7.03 (2H, m, CH$_2$), 3.10 (3H, s, CH$_3$), ^{13}C-NMR (150MHz, DMSO-*d*$_6$); 189.9 (CO), 161.0, 149.9, 138.5, 136.4, 135.9, 131.5, 131.3, 130.2, 129.5, 129.1, 128.0, 127.5, 124.3, 123.3, 122.5, 120.1, 79.6, 63.5(CH$_2$), 20.5(CH$_3$); MS (ESI, Positive): *m/z* = 398.2 (M+H)$^+$; analytical calculated for C$_{20}$H$_{14}$BrF$_6$NO; C, 50.23, H, 2.95, N, 2.93; found; C, 50.19, H, 2.93, N, 2.90.

1.5 1-[2-Phenyl-2-oxoethyl]-4-methylquinolin-1-ium bromide (3e) [1,2]

Appearance; white colour; m.p = 127-128 °C; yield = 88.2 %; ^{1}H-NMR (600MHz, DMSO-d_6); δ = 9.34-9.33 (1H, d; J = 6.6Hz, H_2), 8.61-8.59 (1H, m, H_8), 8.41-8.40 (1H, m, H_5), 8.20-8.18 (2H, m, H_7, H_6), 8.16-8.15 (2H, m, ArH_2, ArH_6), 8.07-8.05 (1H, m, H_3), 7.84-7.69 (3H, m, ArH_3, ArH_4, ArH_5), 6.93 (2H, s, CH_2), 3.09 (3H, s, CH_3); ^{13}C-NMR (150MHz, DMSO-d_6); 191.3 (CO), 160.3, 150.0, 138.4, 135.8, 135.3, 134.1, 130.1, 129.5, 129.1, 129.0, 127.5, 123.2, 120.0, 79.6, 63.2(CH_2), 20.4(CH_3); MS (ESI, Positive): m/z = 262.2 $(M+H)^+$; analytical calculated for $C_{18}H_{16}BrNO$; C, 63.17, H, 4.71, N, 4.09, found; C, 63.11, H, 4.69, N, 4.00

1.6 1-(2-(4-Fluorophenyl)-2-oxoethyl)-4-methylquinolin-1-ium bromide (3f) [3]

Appearance; white colour; m.p. = 138-139 °C; yield = 99.6 %; ^{1}H-NMR (600MHz, DMSO-d_6); δ = 9.32-9.31 (1H, d; J = 6.6Hz, H_2), 8.60-8.58 (1H, m, H_8), 8.46-8.45 (1H, m, H_5), 8.29-8.27 (2H, m, ArH_2, ArH_6), 8.23-8.05 (4H, m, ArH_3, ArH_5, H_7, H_6), 8.01-7.99 (1H, m, H_3), 6.97 (2H, s, CH_2), 3.10 (3H, s, CH_3); ^{13}C-NMR (150MHz, DMSO-d_6); 190.9 (CO), 167.3, 166.2, 150.0, 138.4, 137.4, 135.8, 133.4, 129.6, 129.1, 128.2, 127.5, 125.0, 123.2, 122.5, 120.0, 116.8, 79.6, 63.4CH_2, 20.4 CH_3; MS (ESI, Positive): m/z = 280.2 $(M+H)^+$; analytical calculated for $C_{18}H_{14b}rFNO$; C, 60.02, H, 4.20, N, 3.89; found; C, 59.12, H, 4.10, N, 3.61.

2 General procedure for the preparation of ethyl/methyl-1-(substitutedbenzoyl)-5-methylpyrrolo[1,2-*a*]quinoline-3-carboxylates (4a-k) [4]

To a stirred solution of 1-(2-(substitutedphenyl)-2-oxoethyl)-4-methylquinolin-1-ium bromides (**3a-f**) (0.00237 mol), ethyl/methyl propiolate (0.00237 mol) and K_2CO_3 (0.00593 mol) were added to dry DMF, and stirred at room temperature for 30 minutes. Reaction completion was monitored by TLC. After completion, the reaction mixture was evaporated under reduced pressure and diluted with ethyl acetate. The ethyl acetate layer was washed with brine, water, and dried with anhydrous sodium sulfate. The ethyl acetate was removed under reduced pressure, and the crude compound was purified with a column chromatography using 60–120 mesh silica gel with ethyl acetate and hexane (7:3) as eluent to afford 50.5-66.7% yield of ethyl/methyl-1-(substitutedbenzoyl)-5-methylpyrrolo[1,2-a]quinoline-3-carboxylates. The characterization details of title compounds **4a-k** are reported below.

2.1 Ethyl-1-(4-cyanobenzoyl)-5-methylpyrrolo[1,2-*a*]quinoline-3-carboxylate (4a)

Appearance; black colour; FT-IR (neat cm^{-1}); 2228, 1703, 1629, 1542; ^{1}H-NMR (800MHz, $CDCl_3$); δ = 8.30 (1H, s, H_9), 8.14-8.12 (2H, m, ArH_3, ArH_5), 8.01 (1H, s, H_2), 7.88-7.87 (2H, m, H_6, H_4), 7.65-7.59 (2H, m, ArH_2, ArH_6), 7.58-7.57 (2H, m, H_7, H_8), 4.41-4.37 (2H, q, J = 7.2Hz, CH_2), 2.75 (3H, s, CH_3), 1.42-1.40 (3H, t, J = 7.2Hz, CH_2CH_3); ^{13}C-NMR (200MHz, $CDCl_3$); 182.1 (BnCo), 167.2 (COOEt), 142.4, 141.2, 137.6, 132.9, 132.3, 131.0, 130.3, 128.6, 127.1, 125.7, 125.4, 125.3, 120.6, 118.1, 117.2, 115.8, 107.3, 60.2 (CH_2), 19.6 (CH_3); 14.5 (CH_3); LC-MS (ESI, Positive): m/z = 383.2 $(M+H)^+$; analytical calculated for $C_{24}H_{18}N_2O_3$; C, 75.38, H, 4.74, N, 7.33; found; C, 74.33, H, 4.54, N, 7.31.

2.2 Ethyl-1-(4-bromobenzoyl)-5-methylpyrrolo[1,2-*a*]quinoline-3-carboxylate (4b)

Appearance; yellow colour; FT-IR (neat cm^{-1}); 2973, 1704, 1632, 1539; ^{1}H-NMR (800MHz, $CDCl_3$); δ = 8.25 (1H, s, H_2), 8.14 (1H, m, H_9), 7.98-7.97 (3H, m, H_6, ArH_3, ArH_5), 7.72-7.71 (2H, d, ArH_2, ArH_6), 7.62-7.54 (2H,

m, H_7, H_8), 7.28 (1H, s, H_4), 4.41-4.37 (2H, q, *J* = 7.2Hz, CH_2), 2.73 (3H, s, CH_3), 1.42-1.40 (3H, t, *J* = 7.2Hz, CH_3); ^{13}C-NMR (200MHz, $CDCl_3$); 183.3 (BnCO), 164.1 (COOEt), 140.7 (C_{3a}), 137.4 (C_5), 136.8, 132.9, 131.7, 131.5, 130.0, 128.5, 127.7, 127.3, 125.4, 125.4, 120.5, 117.2, 106.8 (C_3), 60.1 (CH_2), 19.5 (CH_3), 14.5 (CH_3) ; LC-MS (ESI, Positive): *m/z* = 436.2 $(M+H)^+$, 438.2 $(M+2H)^+$; analytical calculated for $C_{23}H_{18}BrNO_3$; C, 63.32, H, 4.16, N, 3.21; found; C, 63.30, H, 4.16, N, 3.28.

2.3 Ethyl-1-(4-fluorobenzoyl)-5-methylpyrrolo[1,2-*a*]quinoline-3-carboxylate (4c)

Appearance; yellow colour; FT-IR (neat cm^{-1}); 2980, 1701, 1636, 1542; ^{1}H-NMR (800MHz, $CDCl_3$); δ = 8.25 (1H, s, H_9), 8.20-8.12 (2H, d, ArH_3, ArH_5), 8.00 (1H, m, H_4), 7.99-7.97 (1H, m, H_6), 7.61-7,54 (3H, s, ArH_2, ArH_4, H_2), 7.27-7.24 (2H, d, H_7, H_8), 4.41-4.37 (2H, q, *J* = 7.2Hz, CH_2), 2.73 (3H, s, CH_3), 1.57-1.27 (3H, t, *J* = 7.2Hz, CH_2CH_3); ^{13}C-NMR (200MHz, $CDCl_3$); 184.6 (BnCO), 164.2 (COOEt), 140.5, 136.6, 134.8, 132.9, 132.5, 132.5, 129.7, 128.5, 127.4, 125.4, 125.2, 120.5, 117.3, 115.7, 115.5, 106.7, 60.1 (CH_2), 19.5 (CH_3), 14.5 (CH_3); LC-MS (ESI, Positive): *m/z* = 376.2 $(M+H)^+$; analytical calculated for $C_{23}H_{18}FNO_3$; C, 73.59, H, 4.83, N, 3.73; found; C, 73.56, H, 4.81, N, 3.77.

2.4 Ethyl-5-methyl-1-(2-nitrobenzoyl)pyrrolo[1,2-*a*]quinoline-3-carboxylate (4d)

Appearance; yellow colour; FT-IR (neat cm^{-1}); 2979, 1705, 1698, 1633; ^{1}H-NMR (800MHz, $CDCl_3$); δ = 8.31(1H, s, H_2), 8.25 (1H, m, H_9), 8.21-8.12 (2H, , ArH_3, ArH_4), 7.69-7.68 (1H, m, H_6), 7.67-7.62 (1H, m, H_7), 7.61-7.53 (3H, m, H_8, ArH_5, ArH_6), 7.28 (1H, s, H_4), 4.40-4.37 (2H, q, *J* = 7.2Hz, CH_2), 2.73 (3H, s, CH_3), 1.42-1.40 (3H, t, *J* = 7.2Hz, CH_2CH_3); ^{13}C-NMR (200MHz, $CDCl_3$); 184.6 (BnCo), 164.2 (COOEt), 140.5, 138.5, 136.5, 132.7, 130.1, 130.1, 129.9, 128.4, 128.4, 127.8, 125.4, 125.3, 125.2, 120.6, 117.3, 106.6, 60.1 (OCH_3), 19.58 (CH_3); LC-MS (ESI, Positive): *m/z* = 403.2 $(M+H)^+$; analytical calculated for $C_{23}H_{18}N_2O_5$; C, 68.65, H, 4.51, N, 6.96; found; C, 68.66, H, 4.58, N, 5.98.

2.5 Ethyl-1-benzoyl-5-methylpyrrolo[1,2-*a*]quinoline-3-carboxylate (4e) [5,6]

Appearance; yellow colour; FT-IR (neat cm^{-1}); 2980, 1700, 1634, 1465; ^{1}H-NMR (800MHz, $CDCl_3$); δ = 8.25 (1H, s, H_2), 8.18-8.10 (3H, m, H_9, ArH_3, ArH_5), 8.01-8.00(1H, m, H_4), 7.90-7.89 (1H, m, ArH_4,) 7.68-7.53 (4h, m, ArH_2, ArH_6, H_6, H_7, H_8), 4.40-4.37 (2H, q, *J* = 7.2Hz, CH_2), 2.73 (3H, s, CH_3), 1.58-1.27 (3H, t, *J* = 7.2Hz, CH_2CH_3); ^{13}C-NMR (200MHz, $CDCl_3$); 184.6 (BnCO), 164.2 (COOEt), 140.5, 138.5, 136.5, 133.0, 132.7, 130.1, 129.9, 128.4, 128.4, 127.8, 125.4, 125.3, 125.2, 120.6, 117.3, 106.6, 60.1 (CH_2), 19.5 (CH_3), 14.5 (CH_3); LC-MS (ESI, Positive): *m/z* = 358.2 $(M+H)^+$; analytical calculated for $C_{23}H_{19}NO_3$; C, 77.29, H, 5.36, N, 3.92; found; C, 77.24, H, 5.21, N, 3.99.

2.6 Dimethyl-1-benzoyl-5-methylpyrrolo[1,2-*a*]quinoline-2,3-dicarboxylate (4f)

Appearance; brick red colour; FT-IR (neat cm^{-1}); 2952, 1726, 1702, 1634, 1445; ^{1}H-NMR (800MHz, $CDCl_3$); δ = 8.21 (1H, s, H_9), 8.02-7.76 (4h, m, ArH_3, ArH_4, ArH_5, H_6, H_7), 7.51-7.43 (4H, m, ArH_2, ArH_6, H_8, H_4), 3.92 (3H, s, OCH_3), 3.45 (3H, s, OCH_3), 2.70 (3H, s, CH_3); ^{13}C-NMR (200MHz, $CDCl_3$); 187.4 (BnCO), 165.3 (COOMe), 163.8 (COOMe), 137.7, 137.4, 135.5, 133.7, 132.3, 129.8, 128.7, 128.6, 128.5, 126.0, 125.6, 125.5,

119.5, 117.3, 104.4, 52.2 (OCH_3), 51.6 (OCH_3), 19.5 (CH_3); LC-MS (ESI, Positive): *m/z* = 402.2 $(M+H)^+$; analytical calculated for $C_{24}H_{19}NO_5$; C, 71.81, H, 4.77, N, 3.49; found; C, 71.82, H, 4.71, N, 3.58.

2.7 Dimethyl-1-(4-cyanobenzoyl)-5-methylpyrrolo[1,2-*a*]quinoline-2,3-dicarboxylate (4g)

Appearance; yellow colour; FT-IR (neat cm^{-1}); 2957, 2232, 1737, 1703, 1646, 1535; ^{1}H-NMR (800MHz, $CDCl_3$); δ = 8.22(1H, d, H_9), 8.21-8.19 (2H, m, ArH_3, ArH_5), 7.78 (1H, s, H_4), 7.61-7.60 (2H, m, ArH_2, ArH_6), 7.51-7.47 (3H, m, H_6, H_7, H_8), 3.93 (3H, s, OCH_3), 3.49 (3H, s, OCH_3), 2.72 (3H, s, CH_3); ^{13}C-NMR (200MHz, $CDCl_3$); 185.0 (BnCO), 165.2 (COOMe), 163.4 (COOMe), 141.1, 138.1, 136.6, 132.3, 132.1, 130.2, 130.0, 128.9, 125.9, 125.7, 125.7, 124.8, 119.5, 117.8, 117.2, 116.6, 104.9, 52.4 (OCH_3), 51.8 (OCH_3), 19.5 (CH_3); LC-MS (ESI, Positive): *m/z* = 427.2 $(M+H)^+$; analytical calculated for $C_{24h18}N_2O_5$; C, 70.42, H, 4.25, N, 6.57; found; C, 70.54, H, 4.20, N, 6.60.

2.8 Dimethyl-5-methyl-1-(2-nitrobenzoyl)pyrrolo[1,2-*a*]quinoline-2,3-dicarboxylate (4h)

Appearance; yellow colour; FT-IR (neat cm^{-1}); 2951, 1726, 1702, 1633, 1605, 1556; ^{1}H-NMR (800MHz, $CDCl_3$); δ = 8.15 (1H, m, H_9), 8.01-7.95 (2H, m, ArH_3, ArH_4), 7.63-7.62 (1H, m, H_6), 7.51-7.50 (1H, s, H_4), 7.49 (1H, m, H_7), 7.48-7.37 (3H, m, H_8, ArH_5, ArH_6), 3.92 (3H, s, $COOCH_3$), 3.45 (3H, s, $COOCH_3$), 2.70 (3H, s, CH_3); ^{13}C-NMR (200MHz, $CDCl_3$); 187.4 (BnCO), 165.3(COOMe), 163.8 (COOMe), 137.7, 137.4, 135.5, 133.7, 132.3, 129.8, 128.7, 128.6, 128.5, 126.0, 125.6, 125.5, 119.5, 117.3, 104.4, 52.2 (OCH_3), 51.6 (OCH_3), 19.5 (CH_3); LC-MS (ESI, Positive): *m/z* = 447.2 $(M+H)^+$; analytical calculated for $C_{24}H_{18}N_2O_7$; C, 64.57, H, 4.06, N, 6.28; found; C, 64.55, H, 4.01, N, 6.31.

2.9 Ethyl-1-(3,5-bis(trifluoromethyl)benzoyl)-5-methylpyrrolo[1,2-*a*]quinoline-3-carboxylate (4i)

Appearance; yellow colour; FT-IR (neat cm^{-1}); 1785, 1780, 1709, 1639; ^{1}H-NMR (800MHz, $CDCl_3$); δ = 8.55 (2H, s, ArH_2, ArH_6), 8.03 (1H, s, H_2), 8.02- 8.01 (1H, m, H_9), 7.66 (1H, m, H_6), 7,66 (1H, s, ArH_4), 7,65 (1H, s, H_4), 7.63-7.58 (2H, m, H_7, H_8), 4.42-4. 38 (2H, q, *J* = 7.2Hz, CH_2), 2.76 (3H, s, CH_3), 1.42-1.40 (3H, t, *J* = 7.2Hz, CH_3); ^{13}C-NMR (200MHz, $CDCl_3$); 180.7 (BnCO), 163.7 (COOEt), 141.5 (CF_3), 140.8 (CF_3), 137.9, 132.8, 132.4, 132.2, 132.04, 131.8, 130.9, 130.0, 129.8, 128.7, 126.5, 120.8, 125.5, 125.4, 123.8, 122.0, 120.4, 117.2, 107.6 (C_3), 60.3 (CH_2), 19.6 (CH_3), 14.4 (CH_3); LC-MS (ESI, Positive): *m/z* = 494.2 $(M+H)^+$; analytical calculated for $C_{24h17}F_6NO_3$; C, 60.86, H, 3.47, N, 2.84; found; C, 60.88, H, 3.43, N, 2.81.

2.10 Dimethyl-1-(4-fluorobenzoyl)-5-methylpyrrolo[1,2-*a*]quinoline-2,3-dicarboxylate (4j)

Appearance; golden yellow colour; FT-IR (neat cm^{-1}); 2956, 1737, 1704, 1643, 1598; ^{1}H-NMR (800MHz, $CDCl_3$); δ = 8.15 (1H, d, H_9), 8.10-7.92 (2H, m, ArH_3, ArH_5), 7.65-7.45 (4H, m, H_6, H_7, H_8, H_4), 7.19-7.16 (2H, s, ArH_2, ArH_6), 3.92 (3H, m, OCH_3), 3.52 (3H, s, OCH_3), 2.70 (3H, s, CH_3); ^{13}C-NMR (200MHz, $CDCl_3$); 185.8 (BnCO), 166.7, 165.4 (COOMe), 163.7 (COOMe), 137.4, 135.6, 134.1, 132.5, 132.5, 132.2, 128.7, 128.5, 125.6, 125.5, 119.4, 117.3, 115.9, 115.8, 104.4, 52.3 (OCH_3), 51.7 (OCH_3), 19.5 (CH_3); LC-MS (ESI, Positive): *m/z* = 420.2 $(M+H)^+$; analytical calculated for $C_{24}H_{18}FNO_5$; C, 68.73, H, 4.33, N, 3.34; found; C, 68.76, H, 4.27, N, 3.38.

2.11 Dimethyl-1-(3,5-bis(trifluoromethyl)benzoyl)-5-methylpyrrolo[1,2-a]quinoline-2,3-dicarboxylate (4k)

Appearance; yellow colour; FT-IR (neat cm^{-1}); 1785, 1780, 1743, 1706, 1650, 1620; ^{1}H-NMR (800MHz, $CDCl_3$); δ = 8.55 (2H, s, ArH_2, ArH_6), 8.21 (1H, s,ArH_4), 8.19 (1H, s, H_4), 8.02-8.01 (1H, m, H_9), 7.67-7.66 (1H, m, H_6), 7.58-7.54 (2H, m, H_7, H_8), 3.92 (3H, s, OCH_3), 3.45 (3H, s, OCH_3), 2.75 (3H, s, CH_3); ^{13}C-NMR (200MHz, $CDCl_3$); 183.2 (BnCO), 165.3 (COOMe), 163.3 (COOMe), 139.6, 138.8, 137.4, 132.3, 132.2, 132.1, 132.0, 131.4, 129.8, 129.0, 126.4, 126.0, 125.8, 125.8, 123.9, 123.4, 122.1, 119.7, 117.0, 105.0, 52.4 (OCH_3), 51.8 (OCH_3), 19.6 (CH_3); LC-MS (ESI, Positive): *m/z* = 538.2 $(M+H)^+$; analytical calculated for $C_{26}H_{17}F_6NO_5$; C, 58.11, H, 3.19, N, 2.61; found; C, 58.06, H, 3.21, N, 2.58..

Table S1: Physicochemical characteristics of substituted pyrrolo[1,2-*a*]quinoline derivatives **4a-k.**

Compound code	Molecular formulae (molecular mass)	R	R[1]	Yield(%)[a,b]	m.p.(°C)
**4a**	$C_{24}H_{18}N_2O_3$ (382)	4-CN	H	50.5	179-180
**4b**	$C_{23}H_{18}BrNO_3$ (435)	4-Br	H	55.0	186-187
**4c**	$C_{23}H_{18}FNO_3$ (375)	4-F	H	56.1	193-194
**4d**	$C_{23}H_{18}N_2O_5$ (402)	2-NO_2	H	54.8	182-186
**4e**	$C_{23}H_{19}NO_3$ (357)	4-H	H	58.9	154-156
**4f**	$C_{24}H_{19}NO_5$ (401)	4-H	$COOCH_3$	65.5	158-160
**4g**	$C_{24}h_{18}N_2O_5$ (426)	4-CN	$COOCH_3$	66.7	184-185
**4h**	$C_{24}H_{18}N_2O_7$ (446)	2-NO_2	$COOCH_3$	61.8	186-188
**4i**	$C_{24}h_{17}F_6NO_3$ (493)	3,5-CF_3	H	53.7	174-175
**4j**	$C_{24}H_{18}FNO_5$ (419)	4-F	$COOCH_3$	63.9	196-197
**4k**	$C_{26}H_{17}F_6NO_5$ (537)	3,5-CF_3	$COOCH_3$	66.5	188-189

[a] All the synthetic compounds were characterized by physical and spectral data.

[b] Yield was calculated after column chromatography purification and confirmation.

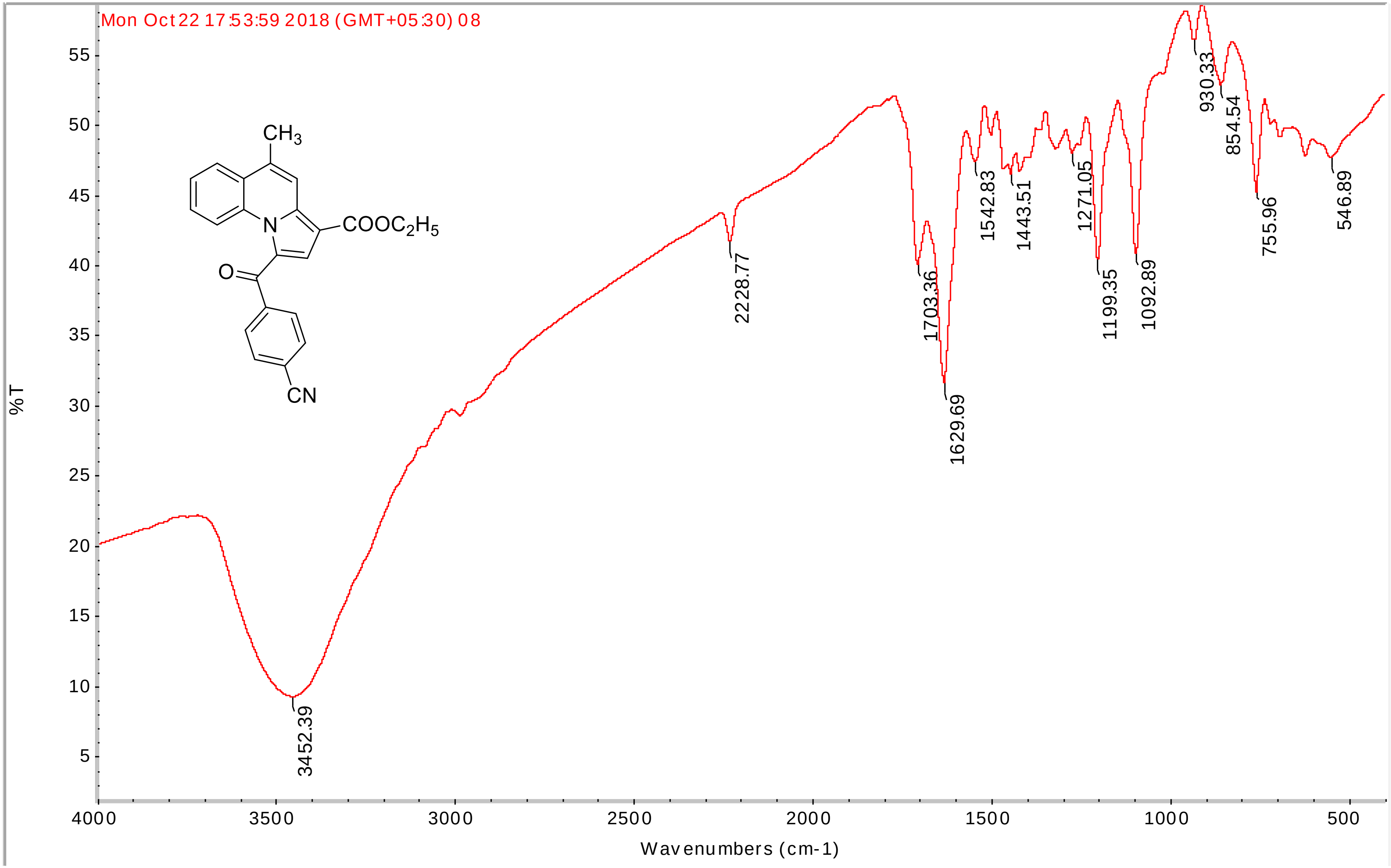

Figure S1: FT-IR of ethyl-1-(4-cyanobenzoyl)-5-methylpyrrolo[1,2-*a*]quinoline-3-carboxylate (**4a**)

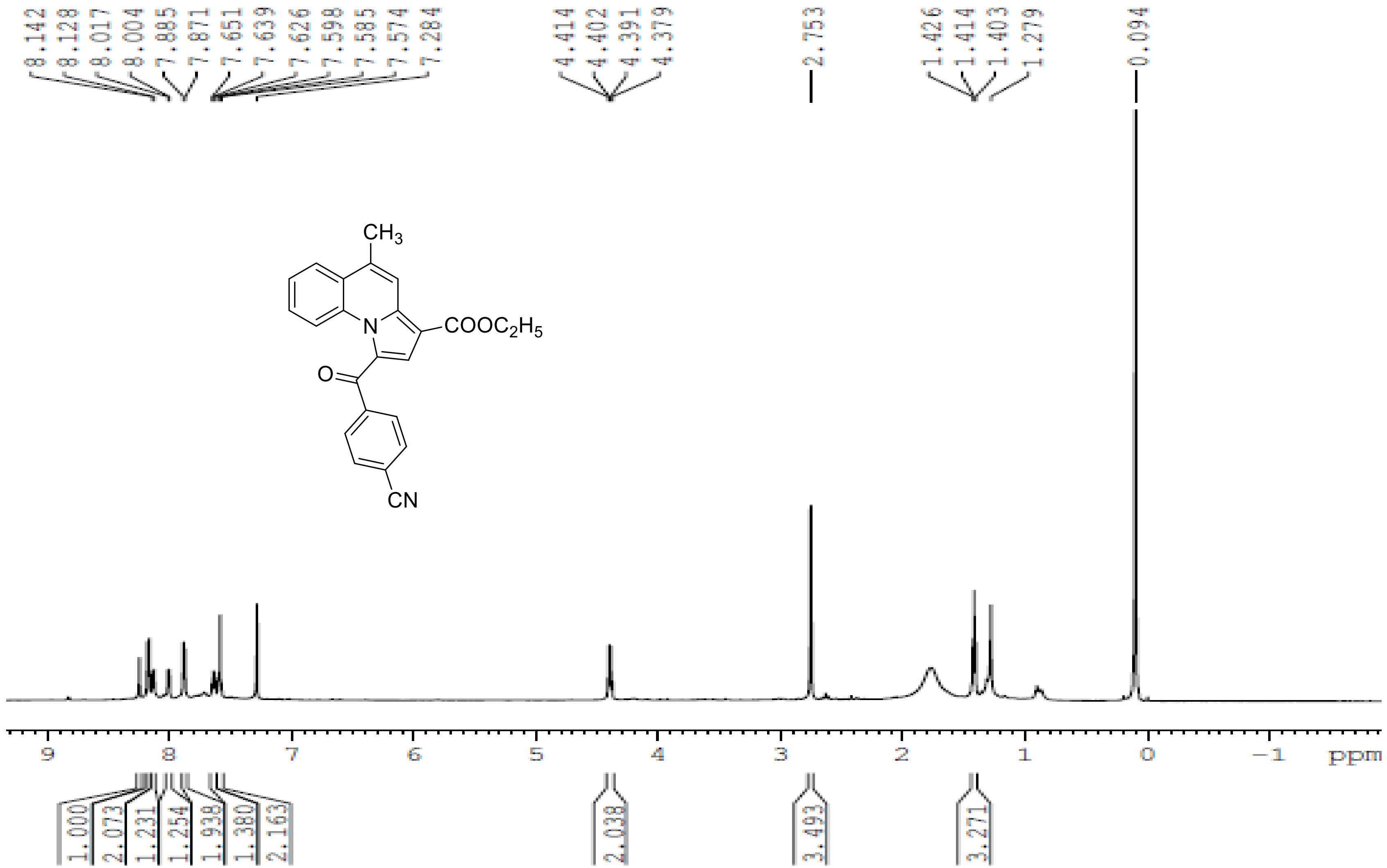

Figure S2: ^{1}H-NMR of ethyl-1-(4-cyanobenzoyl)-5-methylpyrrolo[1,2-*a*]quinoline-3-carboxylate (**4a**)

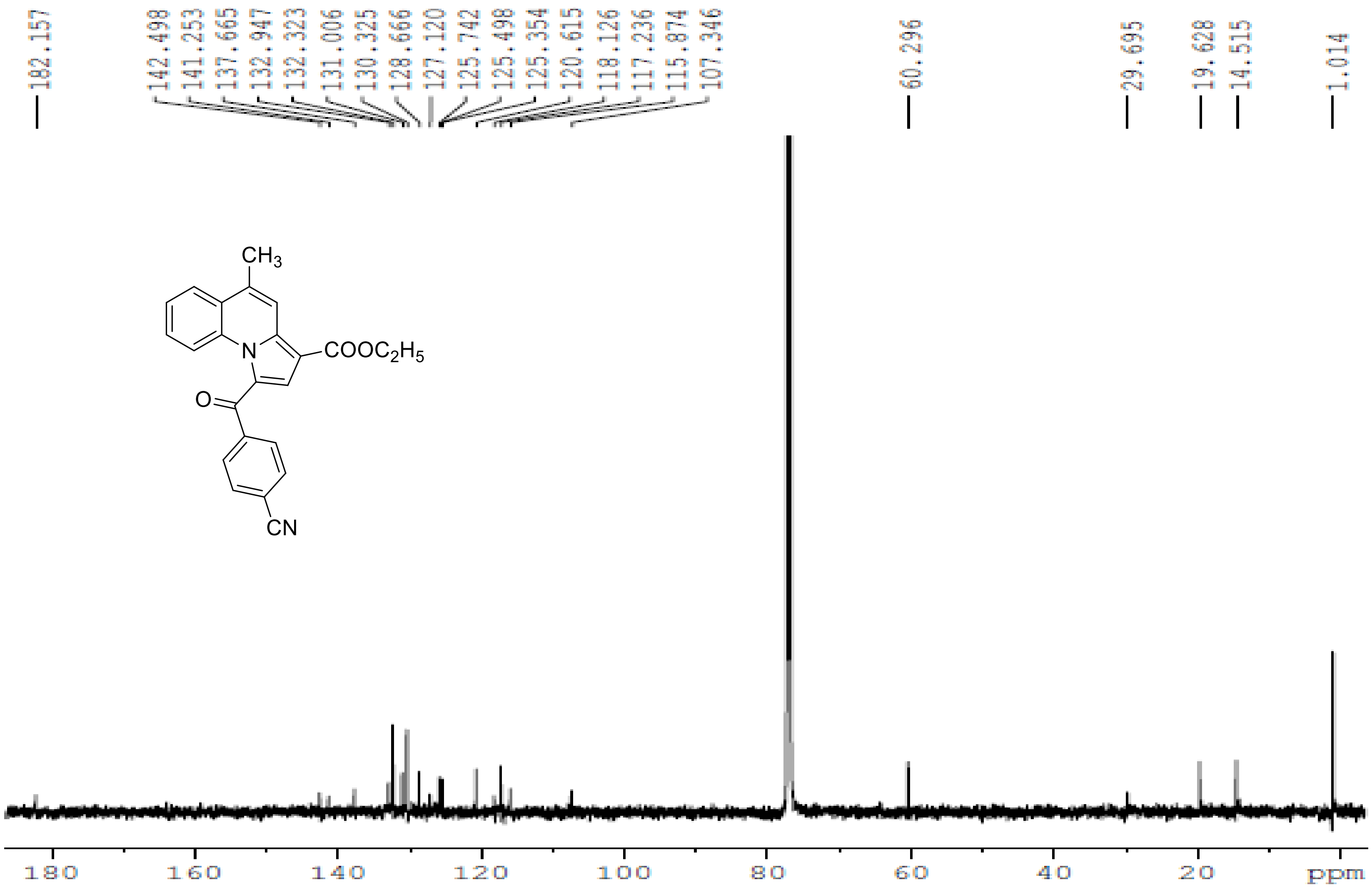

Figure S3: ^{13}C-NMR of ethyl-1-(4-cyanobenzoyl)-5-methylpyrrolo[1,2-*a*]quinoline-3-carboxylate (**4a**)

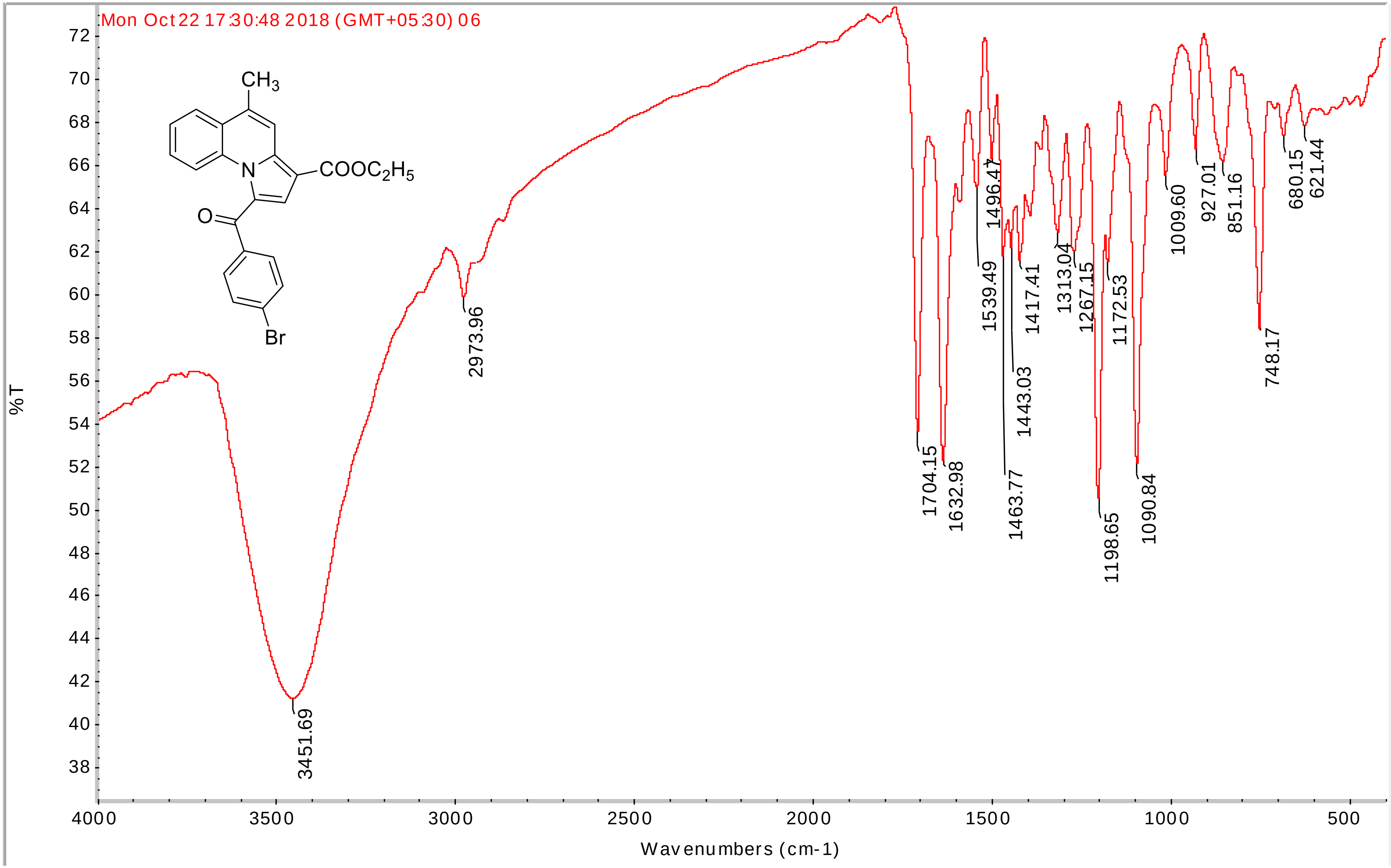

Figure S4: FT-IR of ethyl-1-(4-bromobenzoyl)-5-methylpyrrolo[1,2-*a*]quinoline-3-carboxylate (**4b**)

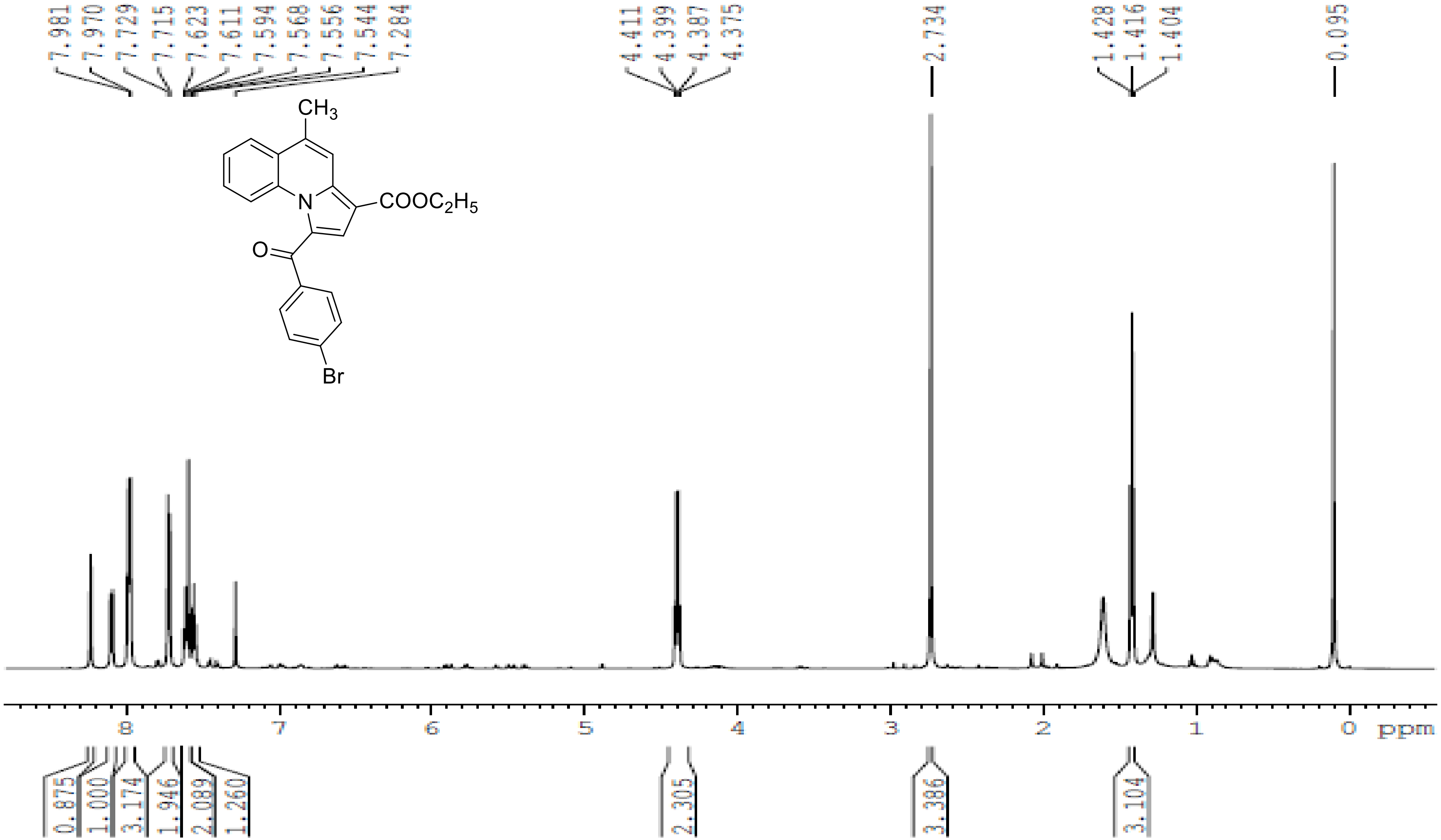

Figure S5: ^{1}H-NMR of ethyl-1-(4-bromobenzoyl)-5-methylpyrrolo[1,2-*a*]quinoline-3-carboxylate (**4b**)

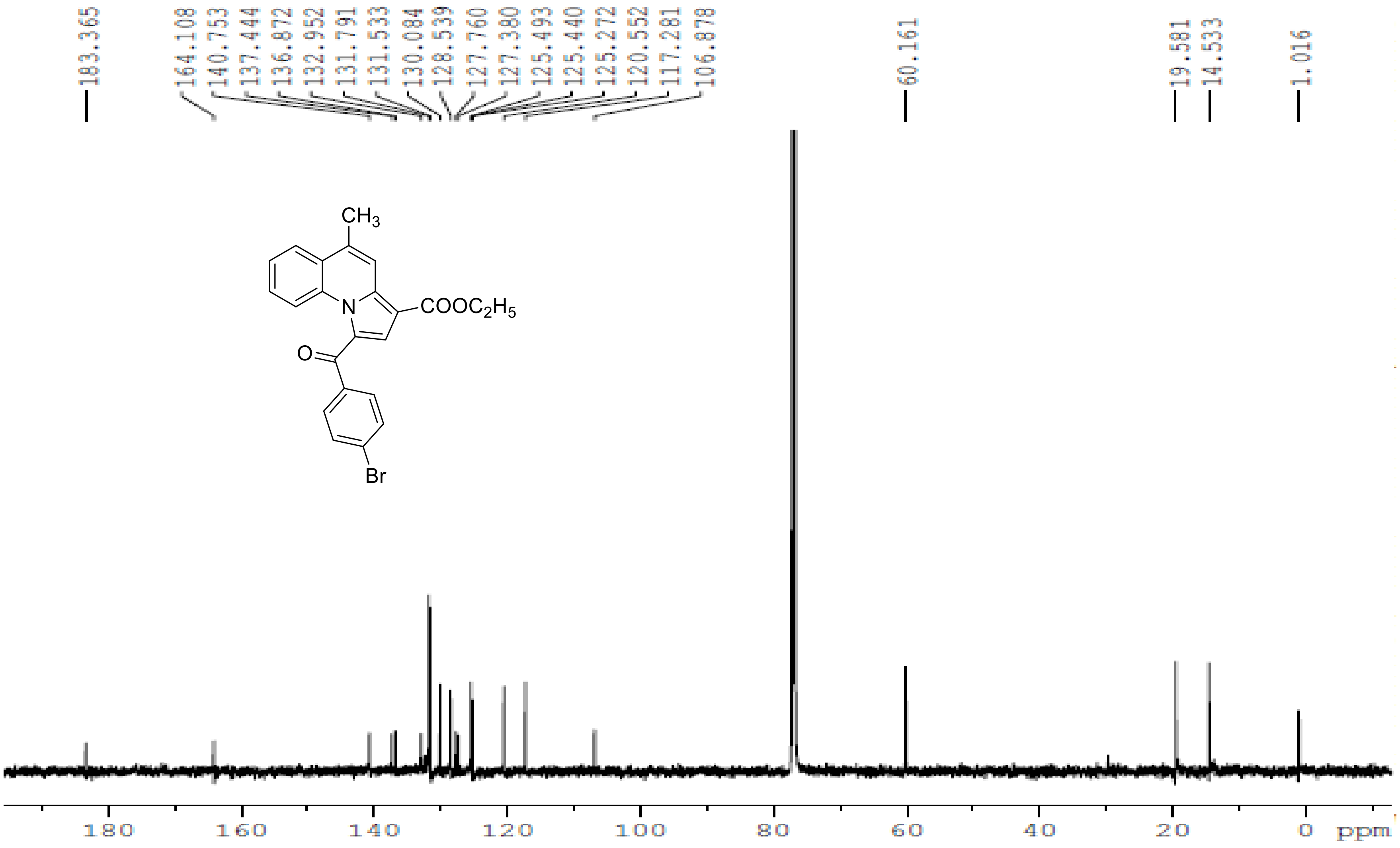

Figure S6: ^{13}C-NMR of ethyl-1-(4-bromobenzoyl)-5-methylpyrrolo[1,2-*a*]quinoline-3-carboxylate (**4b**)

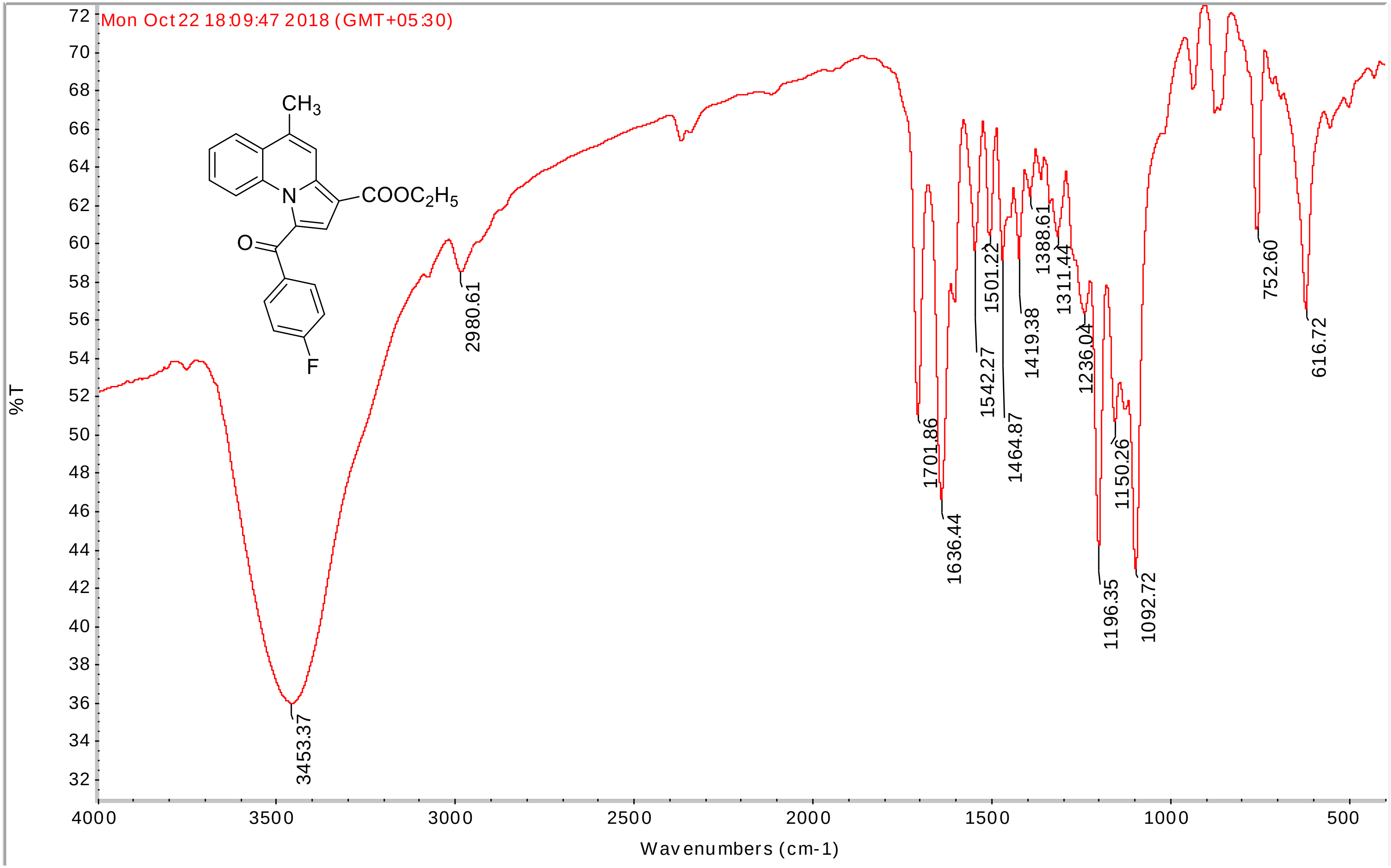

Figure S7: FT-IR of ethyl-1-(4-fluorobenzoyl)-5-methylpyrrolo[1,2-*a*]quinoline-3-carboxylate (**4c**)

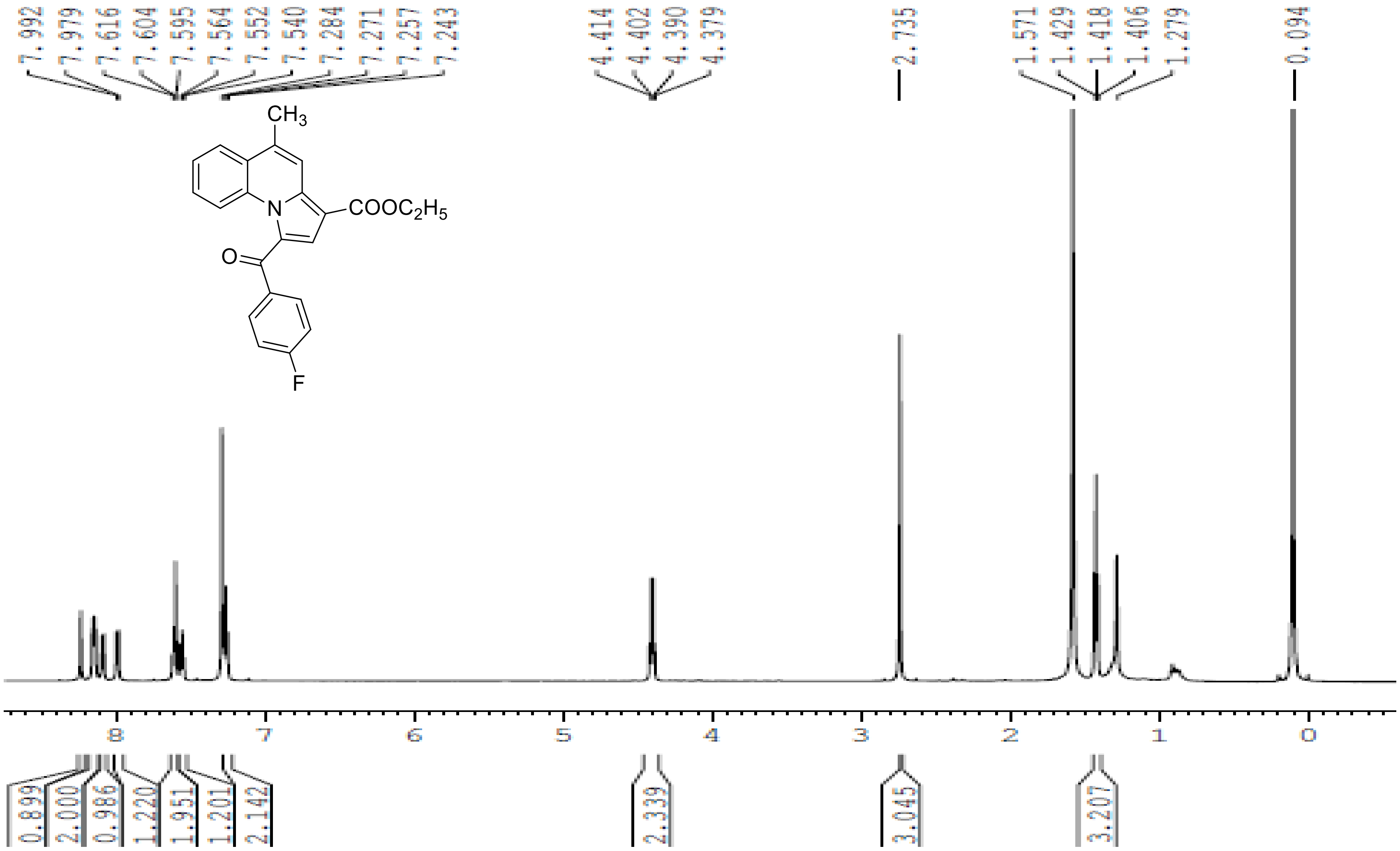

Figure S8: ^{1}H-NMR of ethyl-1-(4-fluorobenzoyl)-5-methylpyrrolo[1,2-*a*]quinoline-3-carboxylate (**4c**)

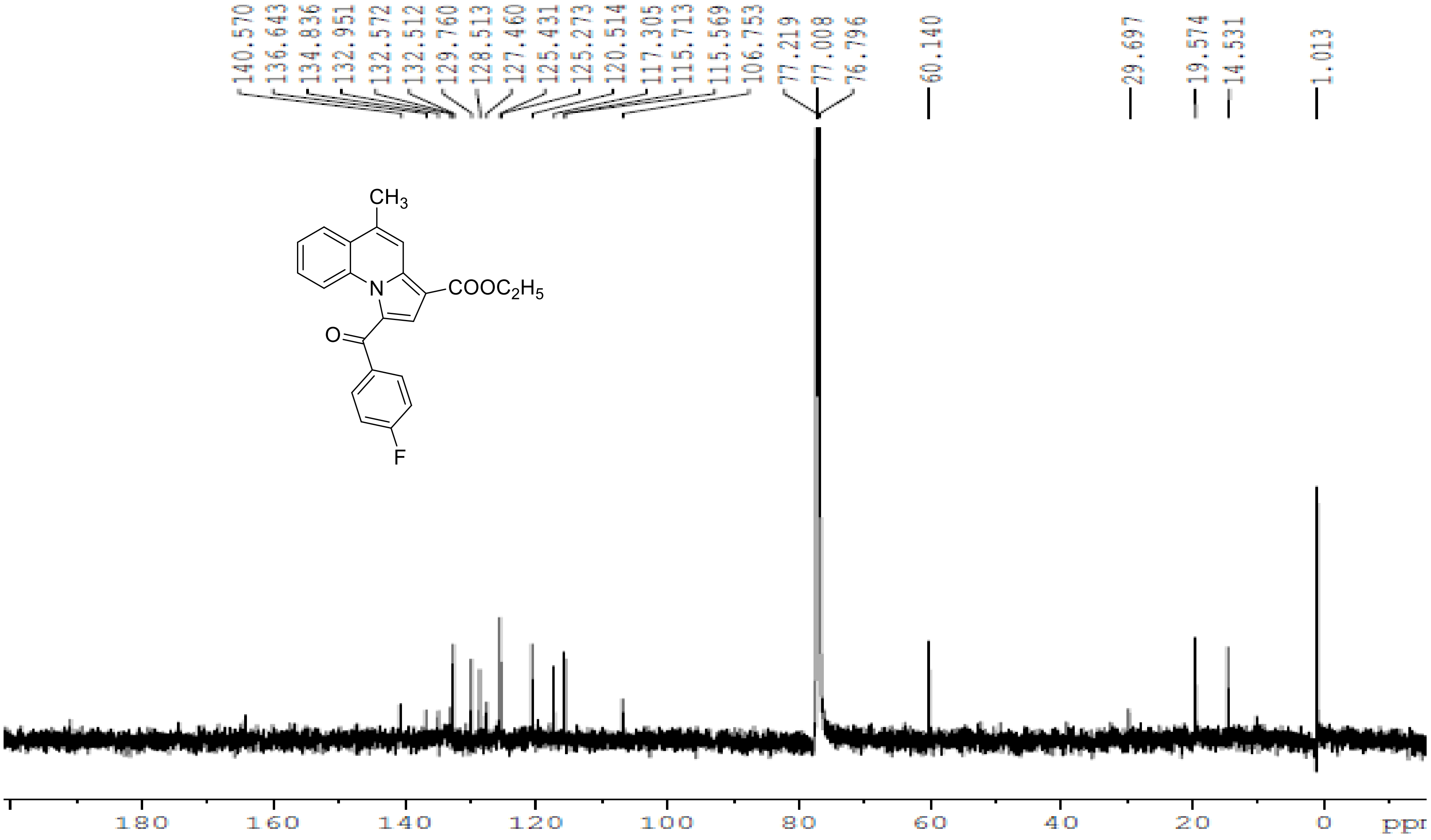

Figure S9: ^{13}C-NMR of ethyl-1-(4-fluorobenzoyl)-5-methylpyrrolo[1,2-*a*]quinoline-3-carboxylate (**4c**)

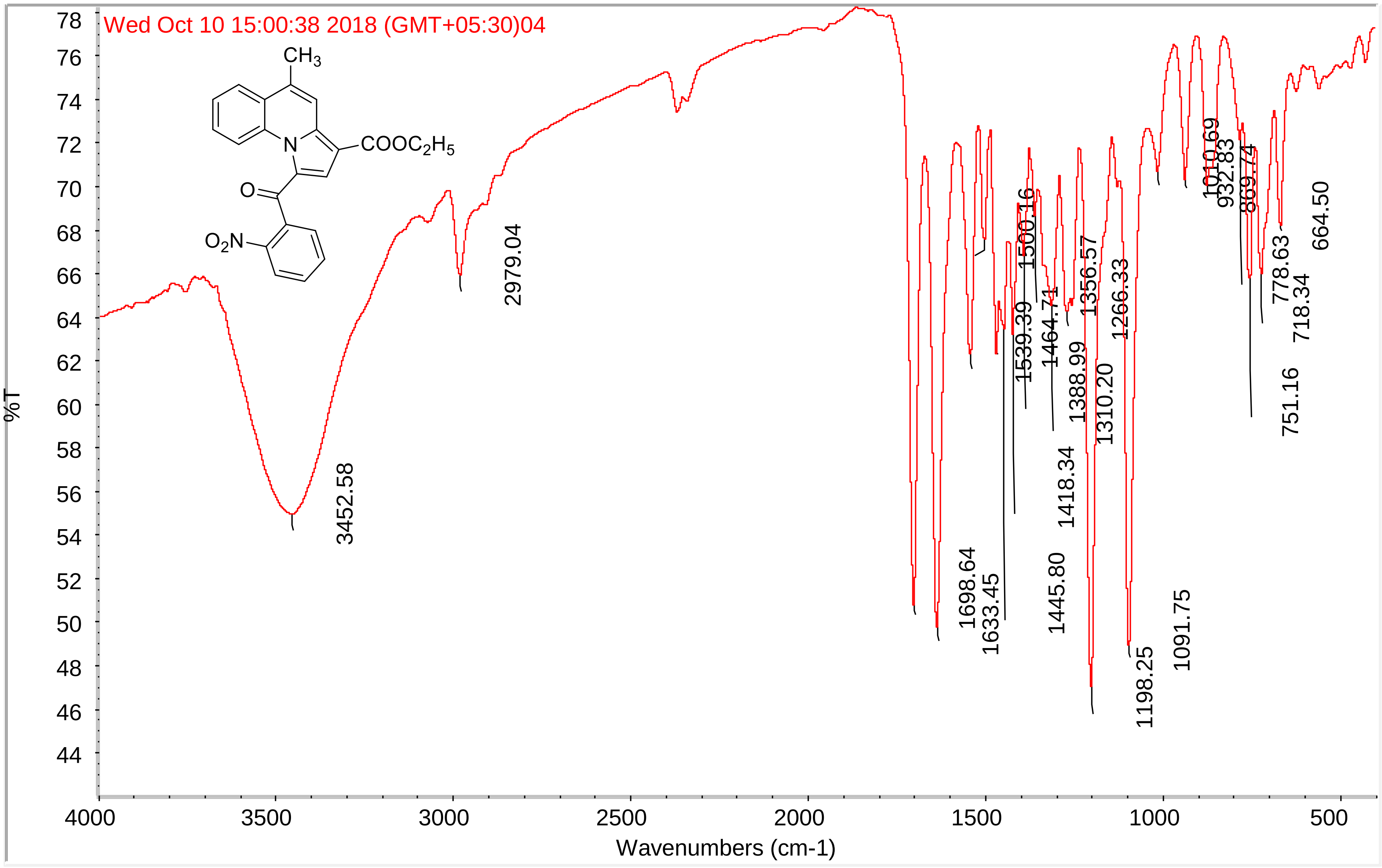

Figure S10: FT-IR of ethyl-5-methyl-1-(2-nitrobenzoyl)pyrrolo[1,2-*a*]quinoline-3-carboxylate (**4d**)

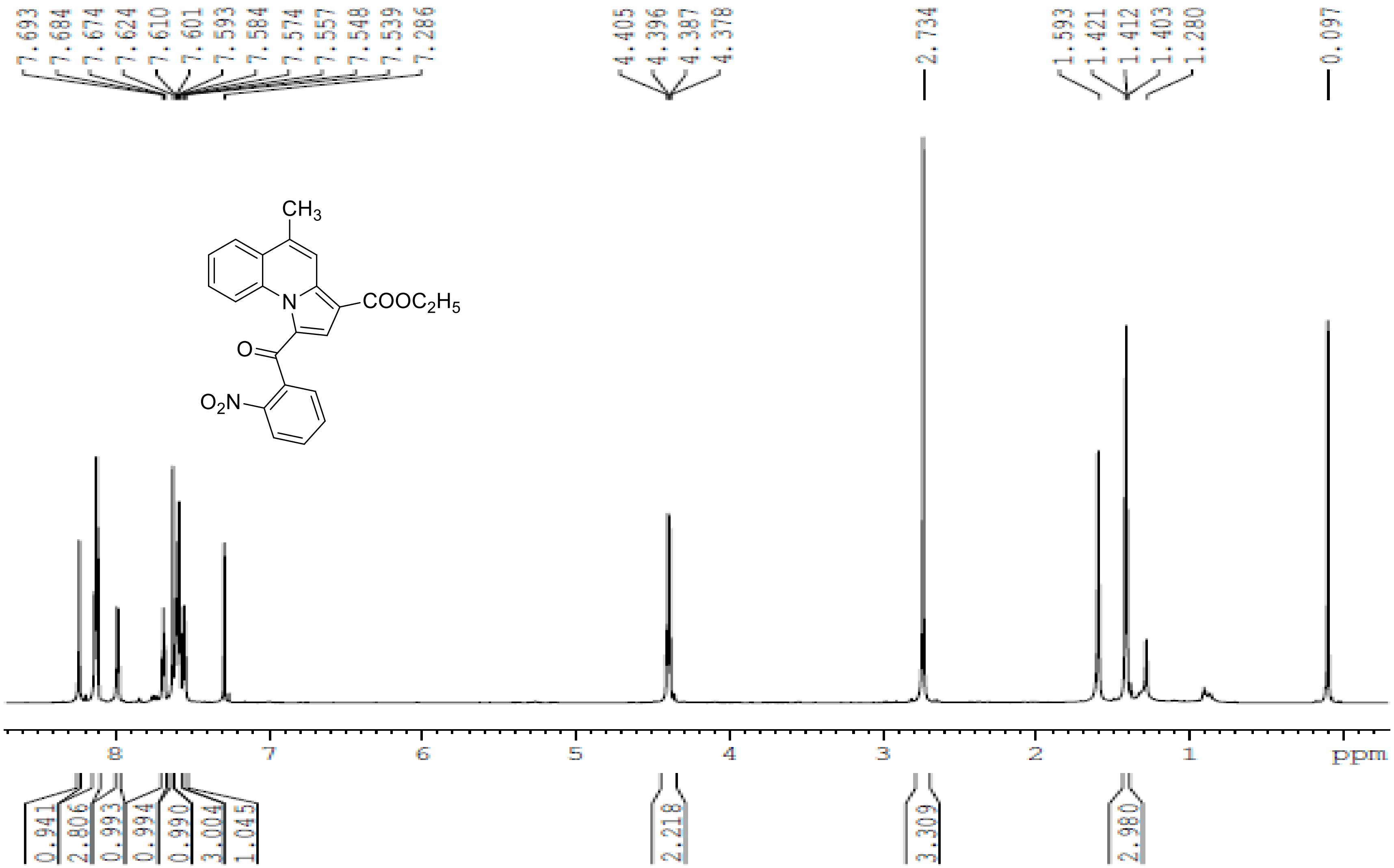

Figure S11: ^{1}H-NMR of ethyl-5-methyl-1-(2-nitrobenzoyl)pyrrolo[1,2-*a*]quinoline-3-carboxylate (**4d**)

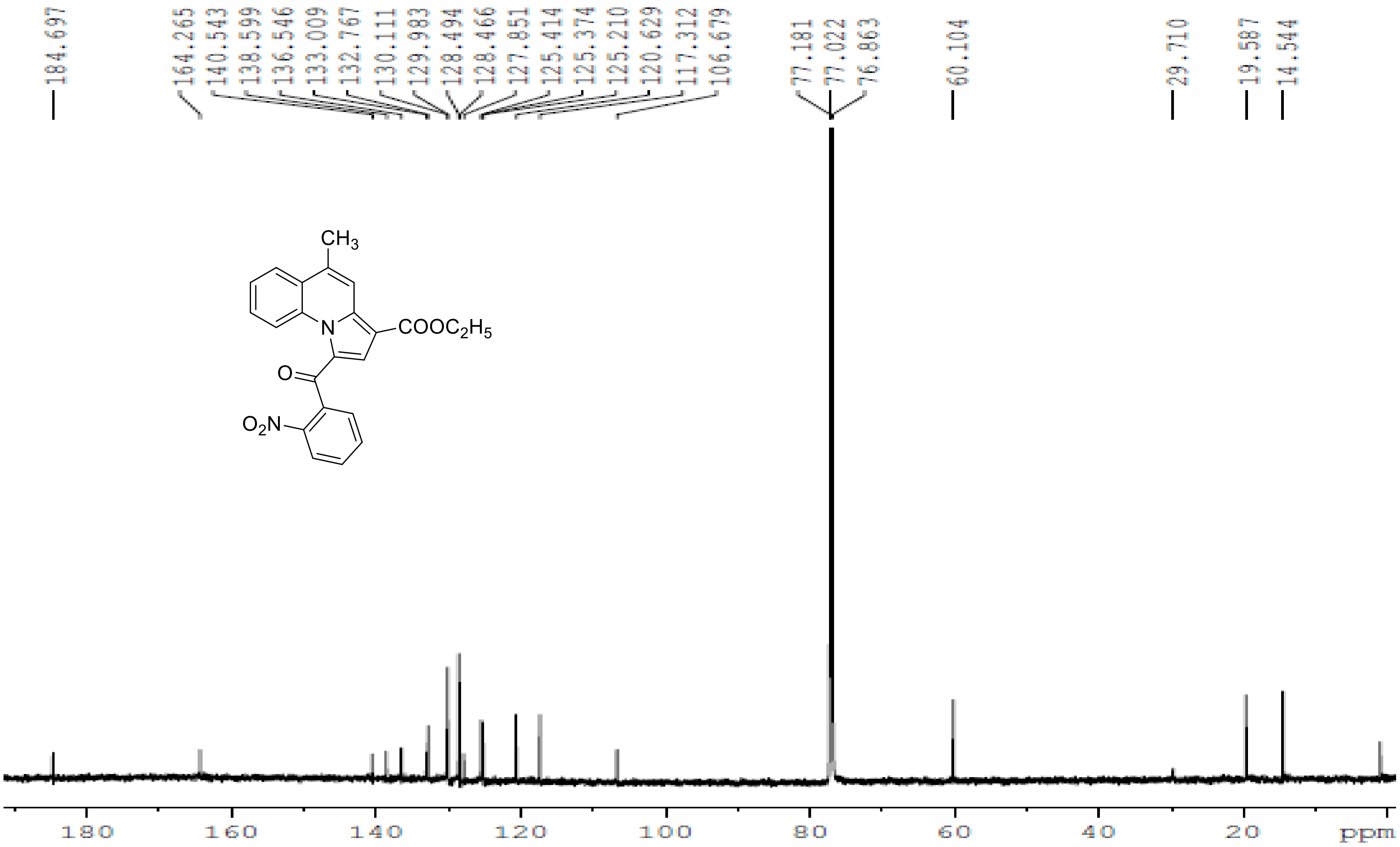

Figure S12: ^{13}C-NMR of ethyl-5-methyl-1-(2-nitrobenzoyl)pyrrolo[1,2-*a*]quinoline-3-carboxylate (**4d**)

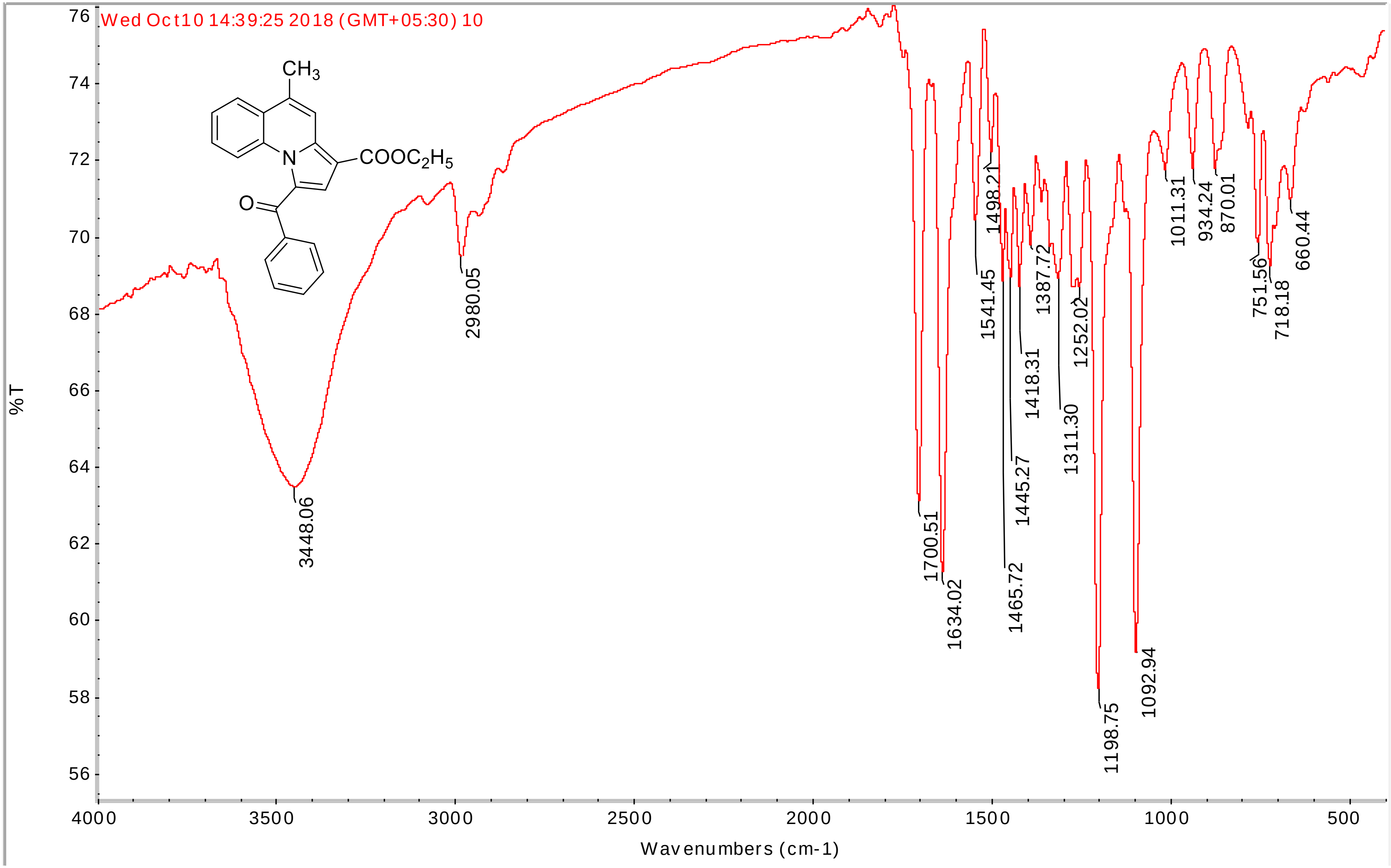

Figure S13: FT-IR of ethyl-1-benzoyl-5-methylpyrrolo[1,2-*a*]quinoline-3-carboxylate (**4e**)

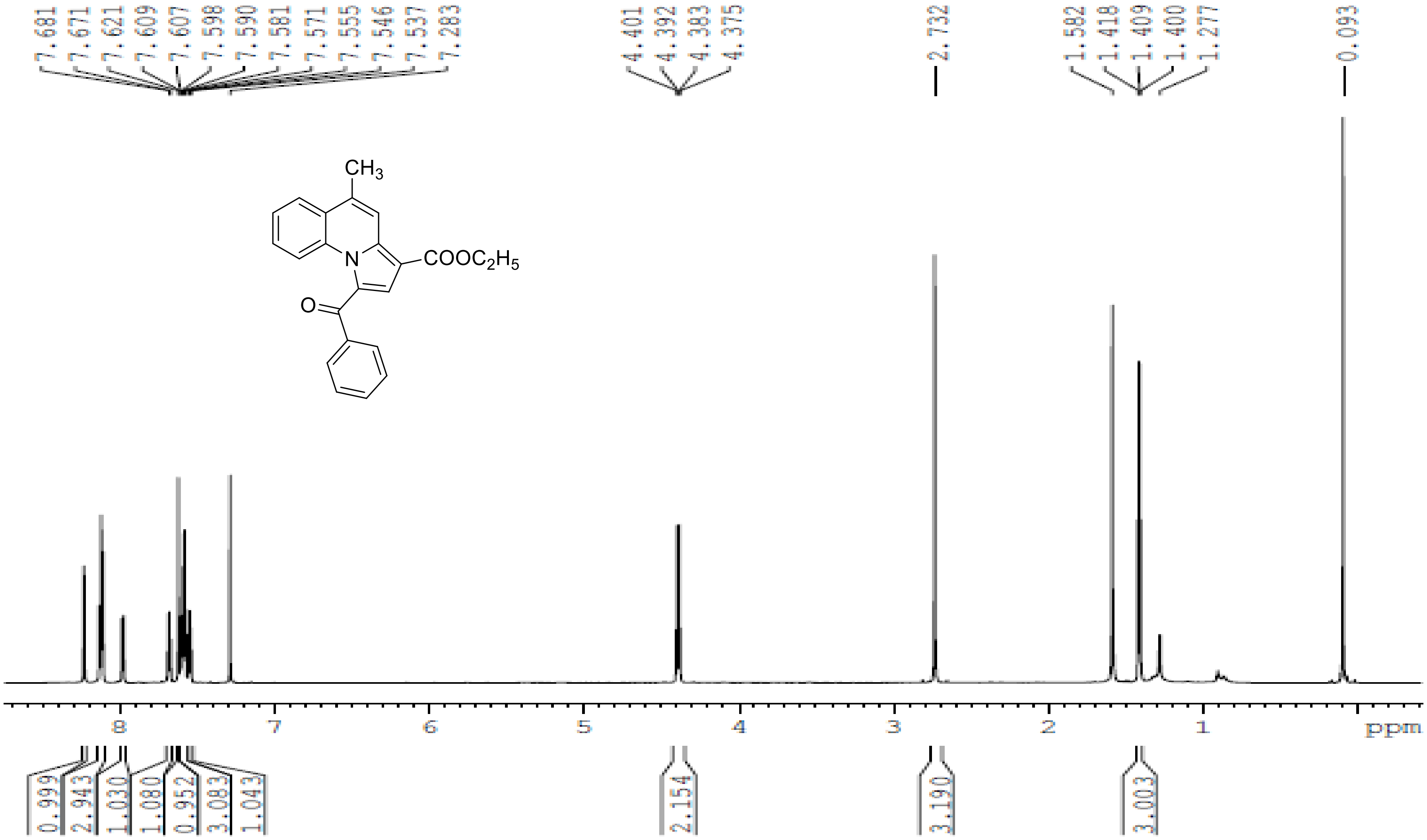

Figure S14: ^{1}H-NMR of ethyl-1-benzoyl-5-methylpyrrolo[1,2-*a*]quinoline-3-carboxylate (**4e**)

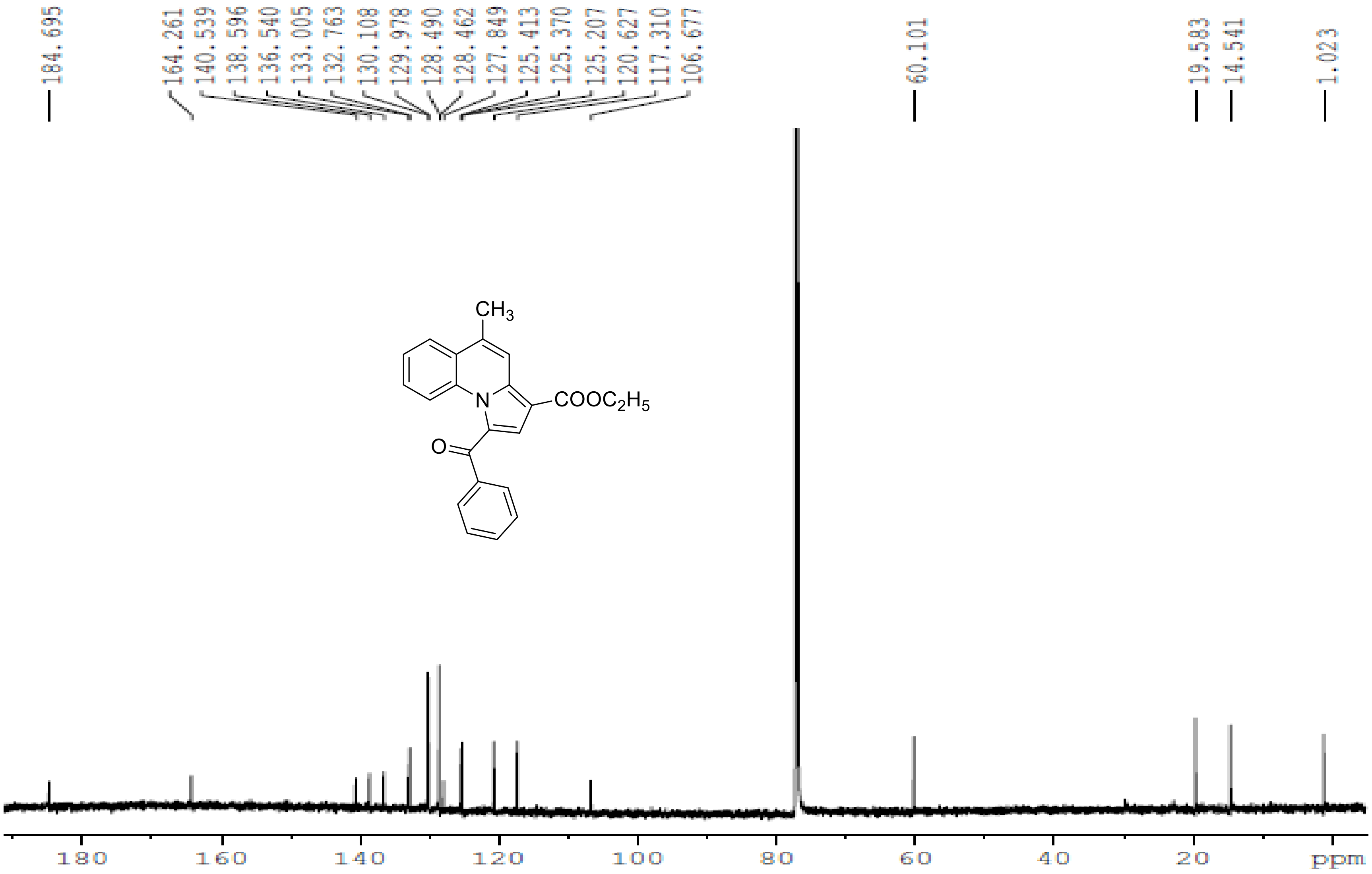

Figure S15: ^{13}C-NMR of ethyl-1-benzoyl-5-methylpyrrolo[1,2-*a*]quinoline-3-carboxylate (**4e**)

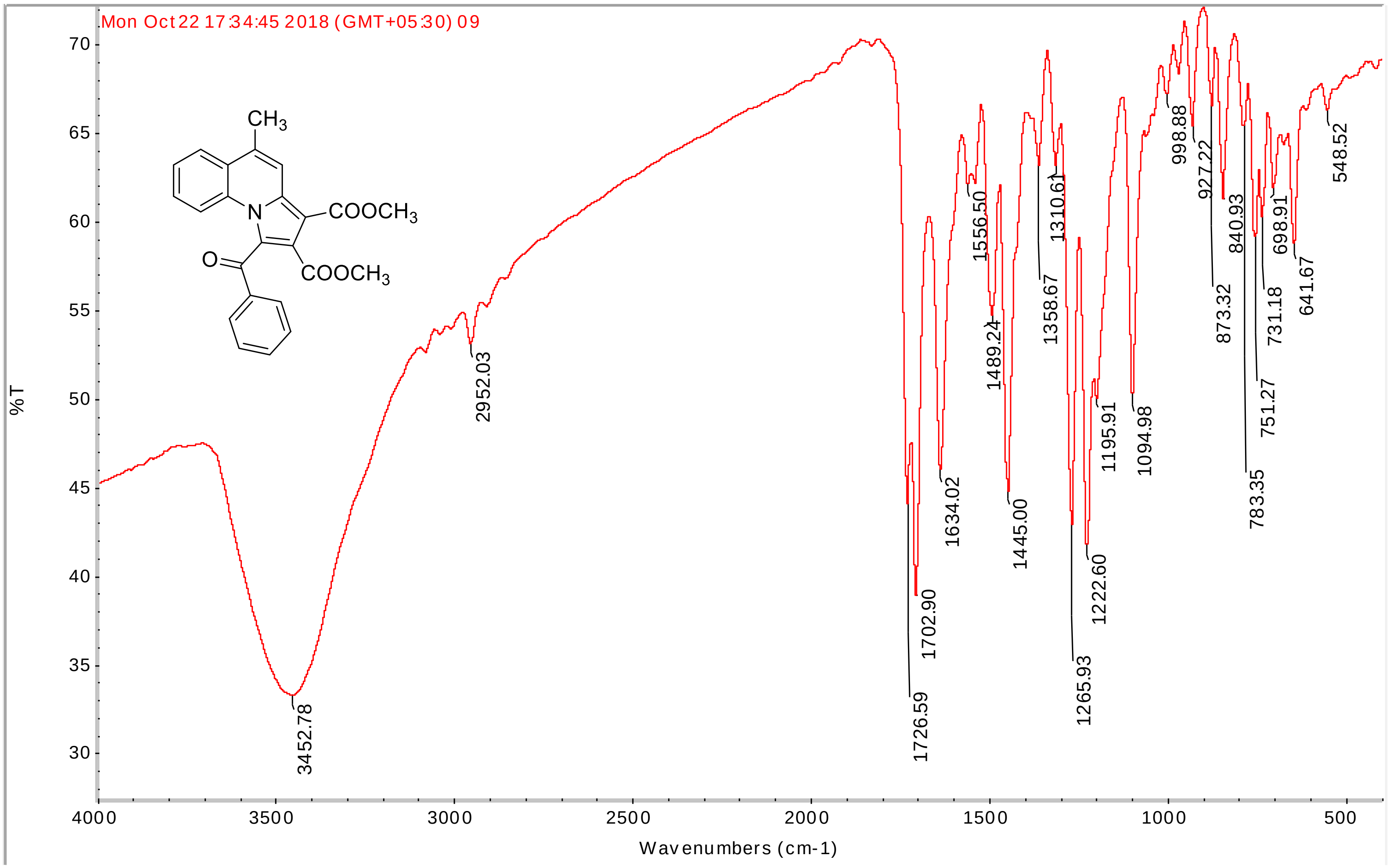

Figure S16: FT-IR of dimethyl-1-benzoyl-5-methylpyrrolo[1,2-*a*]quinoline-2,3-dicarboxylate (**4f**)

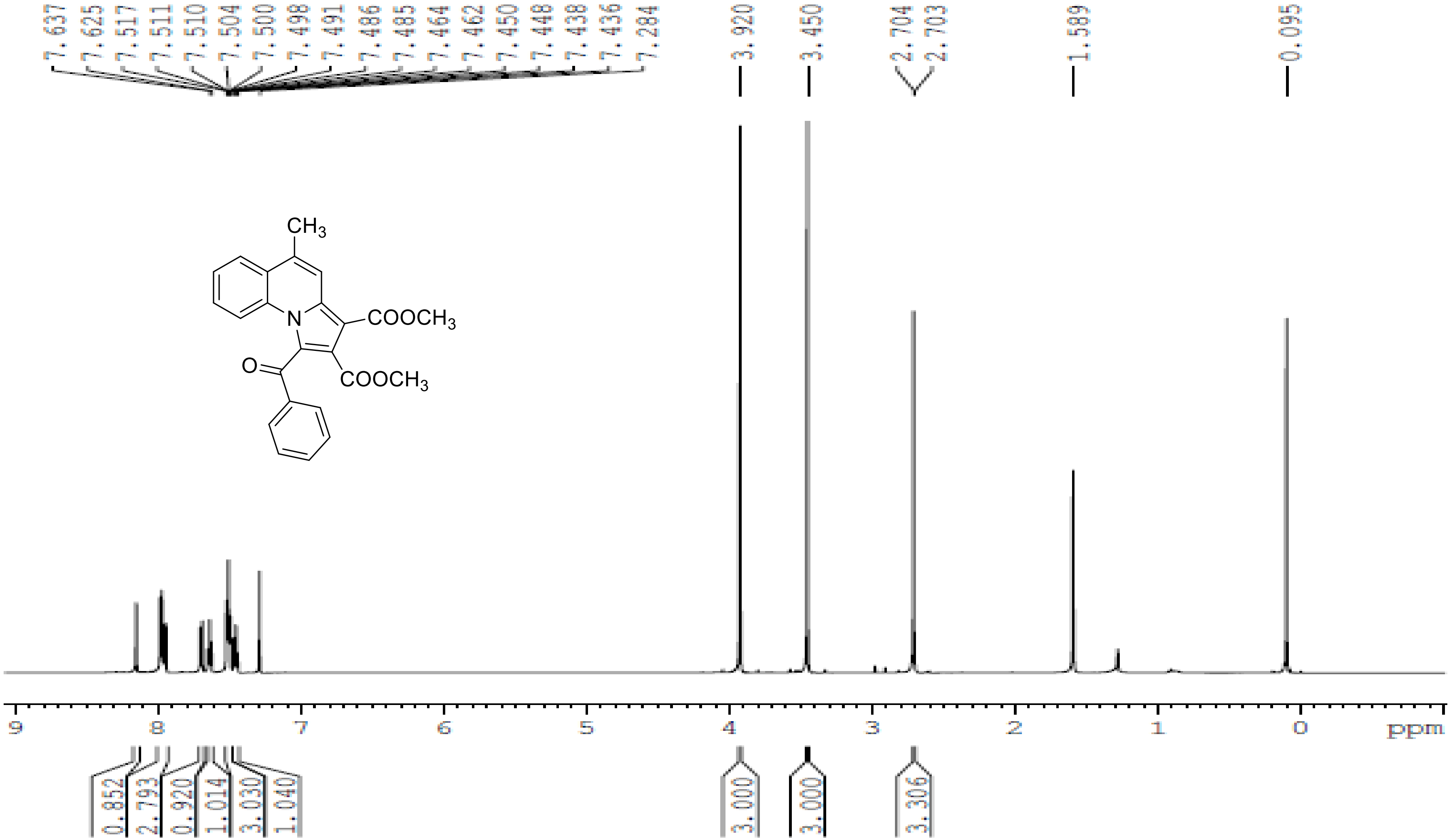

Figure S17: ^{1}H-NMR of dimethyl-1-benzoyl-5-methylpyrrolo[1,2-*a*]quinoline-2,3-dicarboxylate (**4f**)

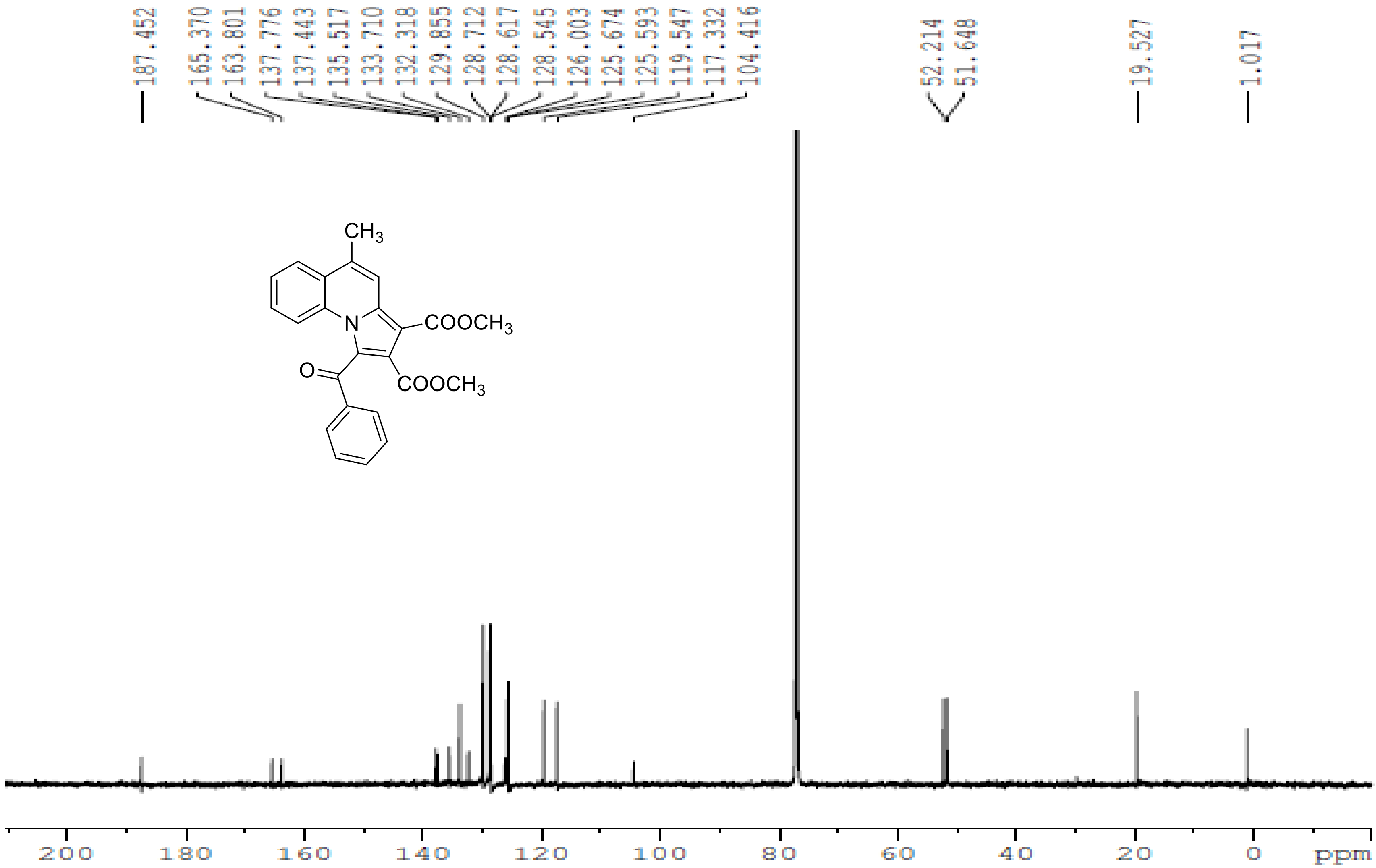

Figure S18: ^{13}C-NMR of dimethyl-1-benzoyl-5-methylpyrrolo[1,2-*a*]quinoline-2,3-dicarboxylate (**4f**)

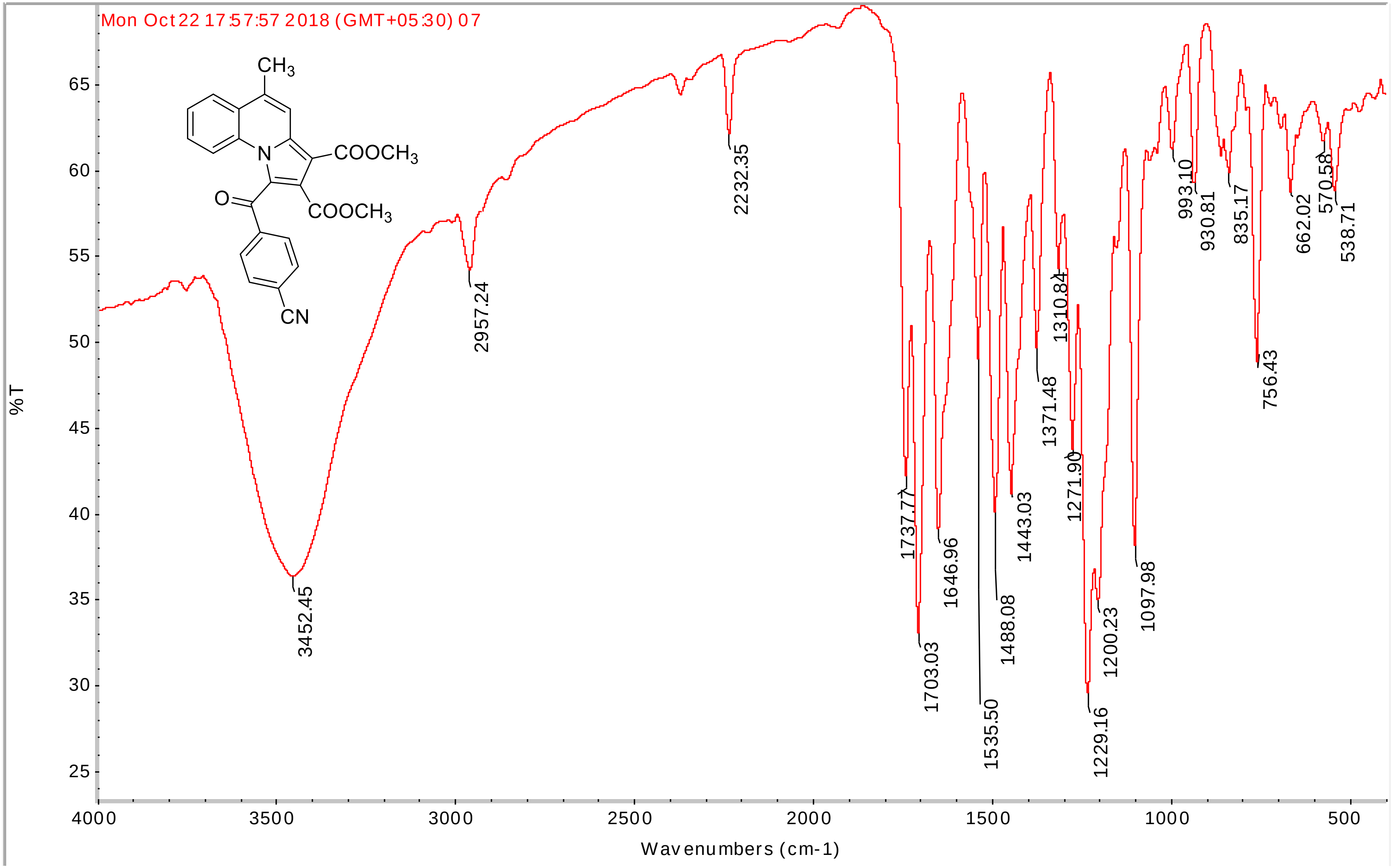

Figure S19: FT-IR of dimethyl-1-(4-cyanobenzoyl)-5-methylpyrrolo[1,2-*a*]quinoline-2,3-dicarboxylate (**4g**)

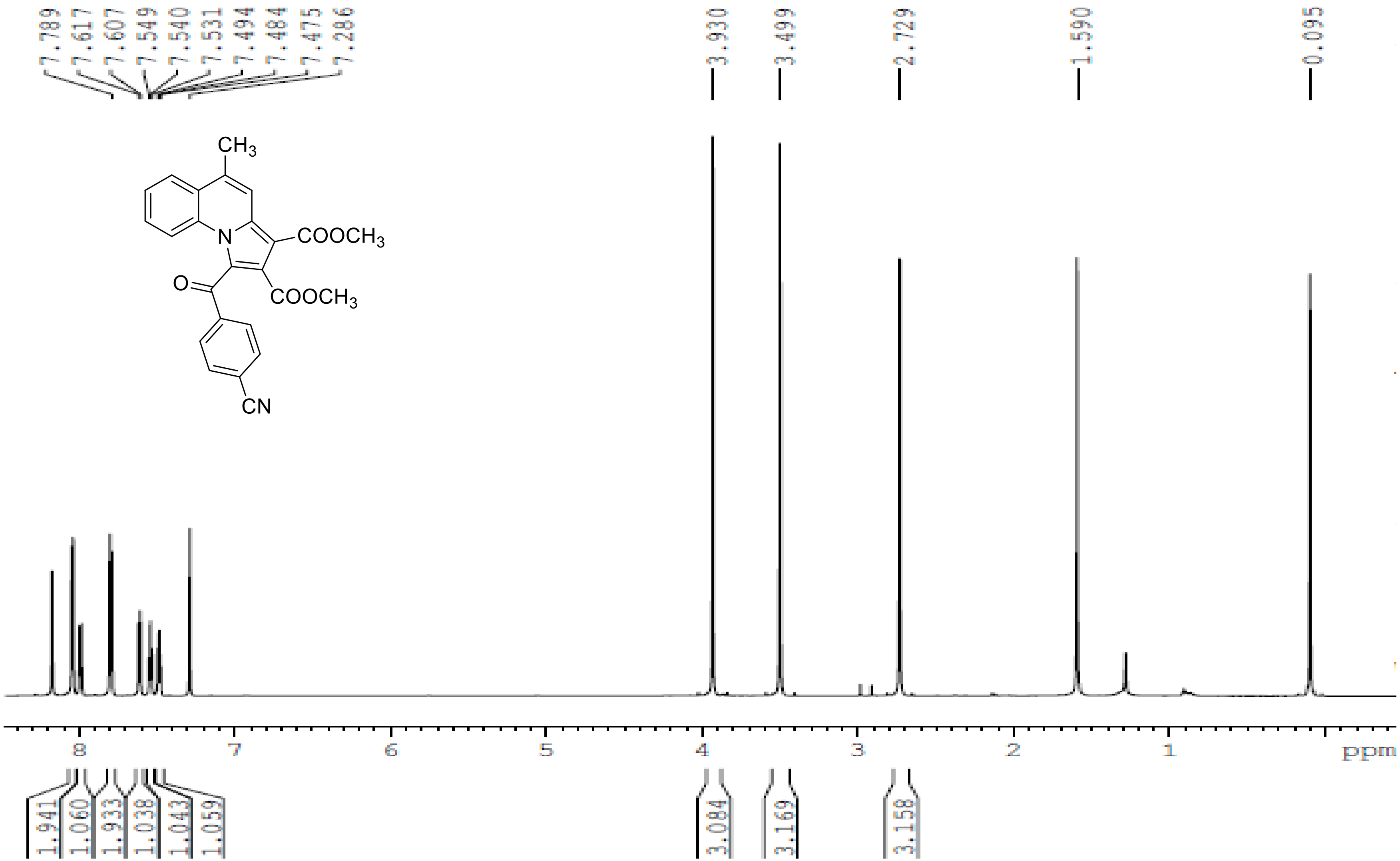

Figure S20: ^{1}H-NMR of dimethyl-1-(4-cyanobenzoyl)-5-methylpyrrolo[1,2-*a*]quinoline-2,3-dicarboxylate (**4g**)

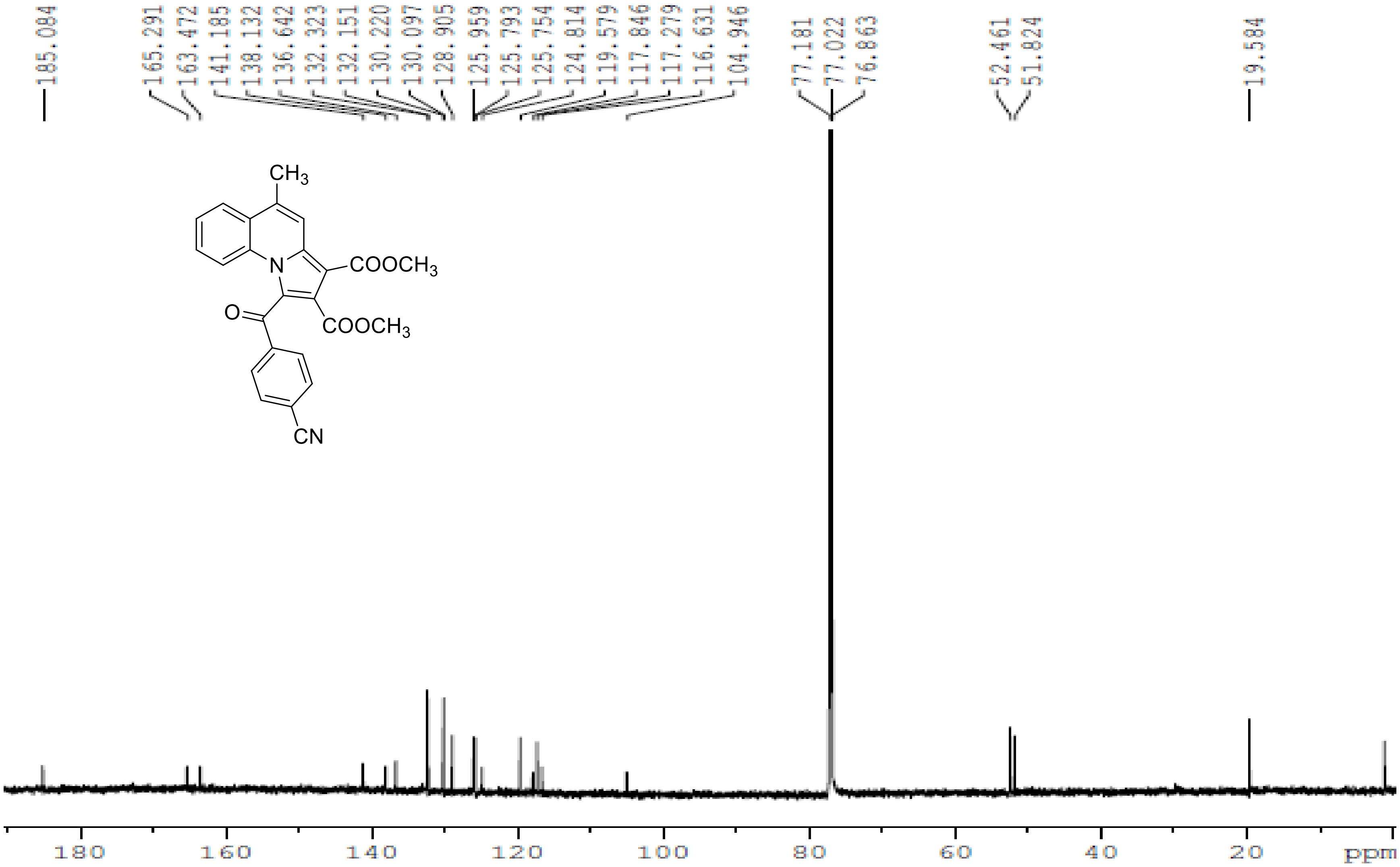

Figure S21: ^{13}C-NMR of dimethyl-1-(4-cyanobenzoyl)-5-methylpyrrolo[1,2-*a*]quinoline-2,3-dicarboxylate (**4g**)

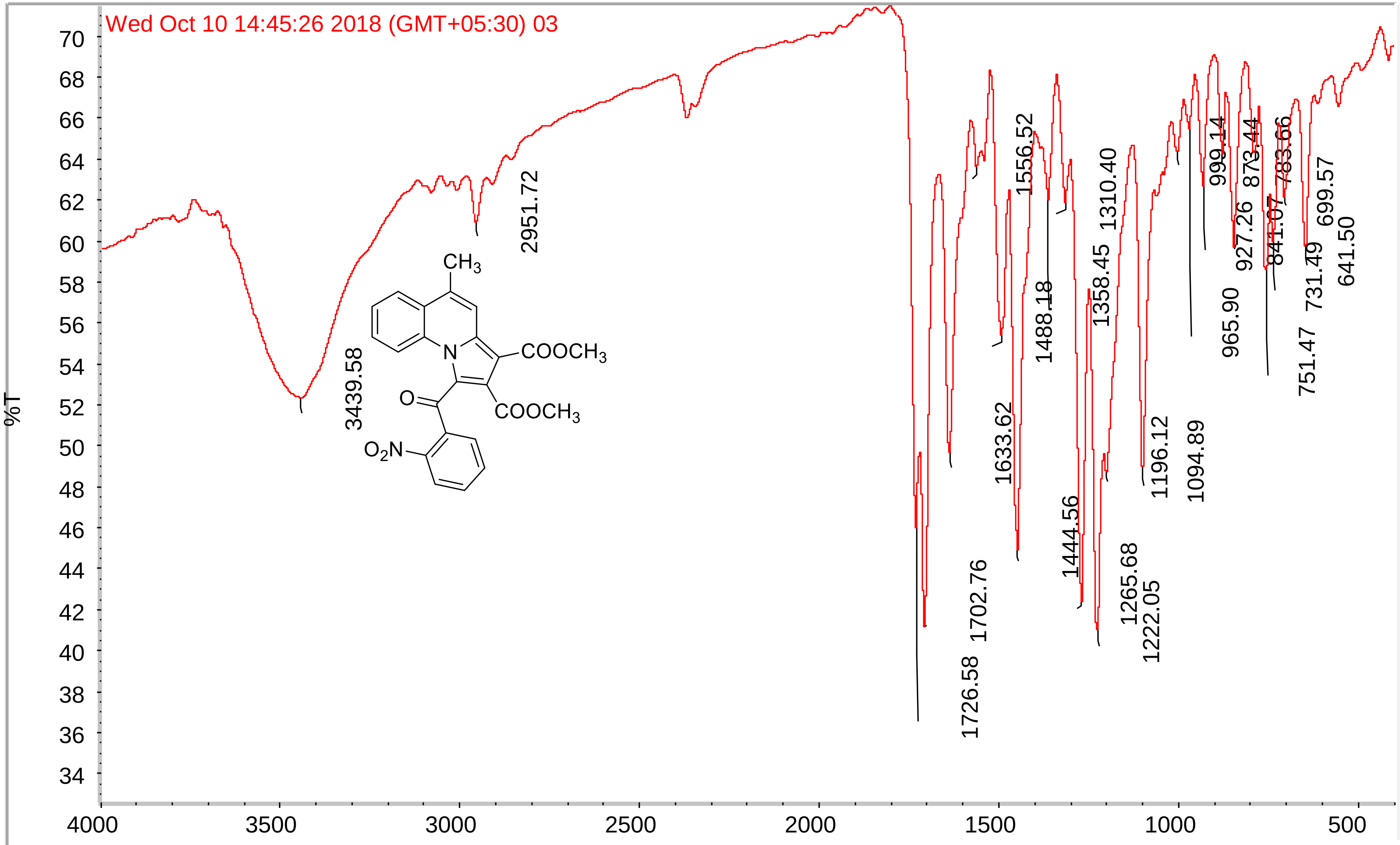

Figure S22: FT-IR of dimethyl-5-methyl-1-(2-nitrobenzoyl)pyrrolo[1,2-*a*]quinoline-2,3-dicarboxylate (**4h**)

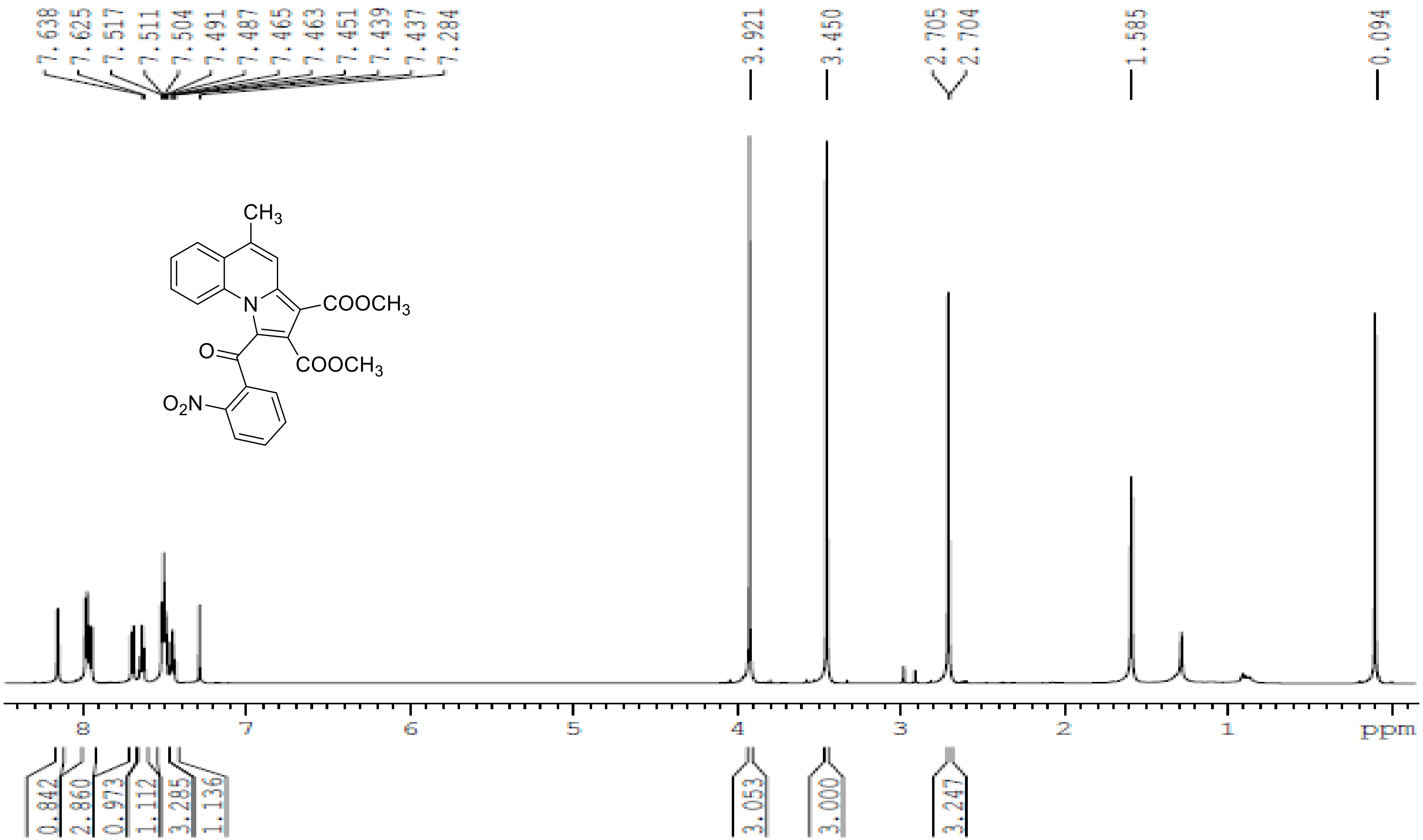

Figure S23: ^{1}H-NMR of dimethyl-5-methyl-1-(2-nitrobenzoyl)pyrrolo[1,2-*a*]quinoline-2,3-dicarboxylate (**4h**)

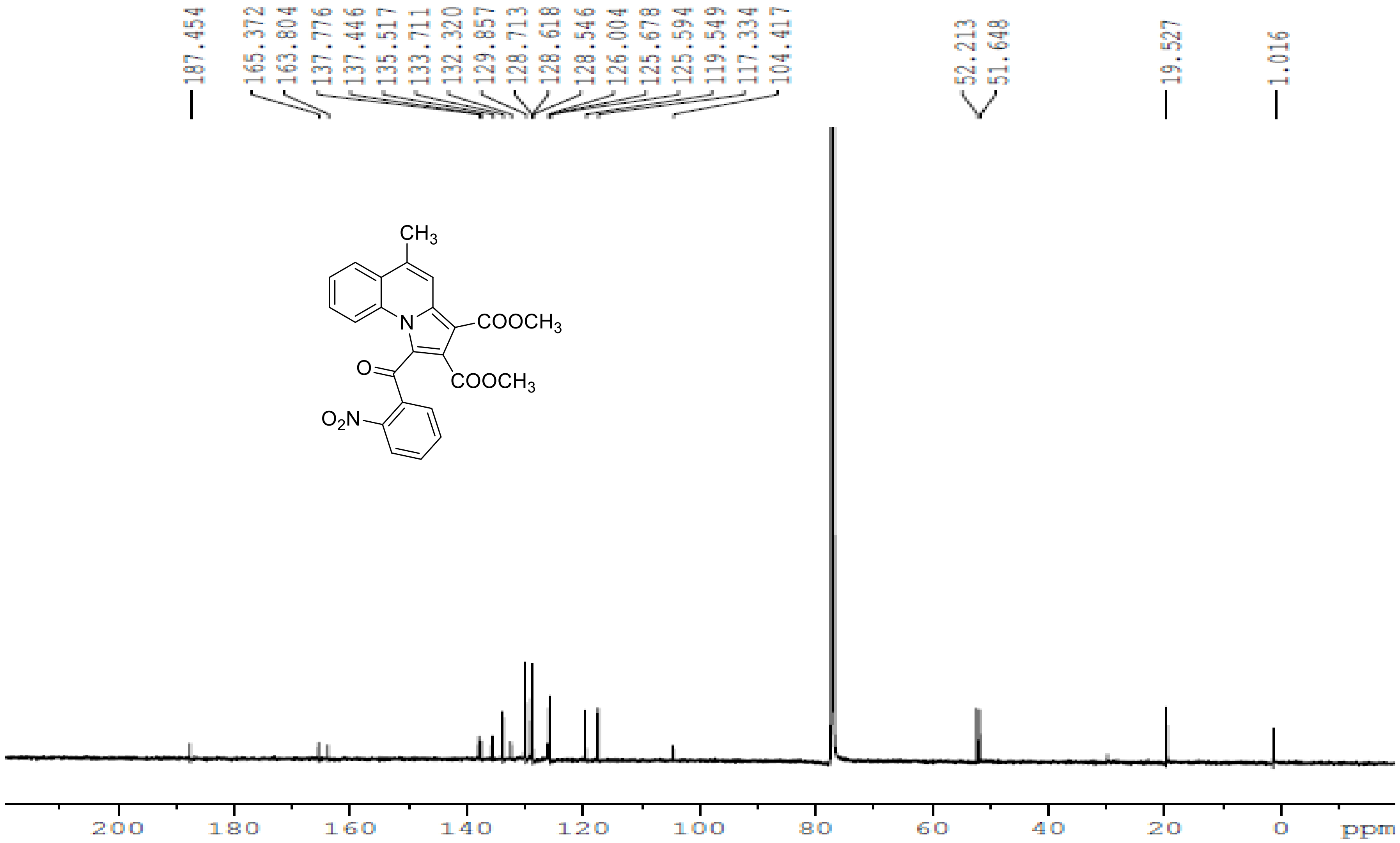

Figure S24: ^{13}C-NMR of dimethyl-5-methyl-1-(2-nitrobenzoyl)pyrrolo[1,2-*a*]quinoline-2,3-dicarboxylate (**4h**)

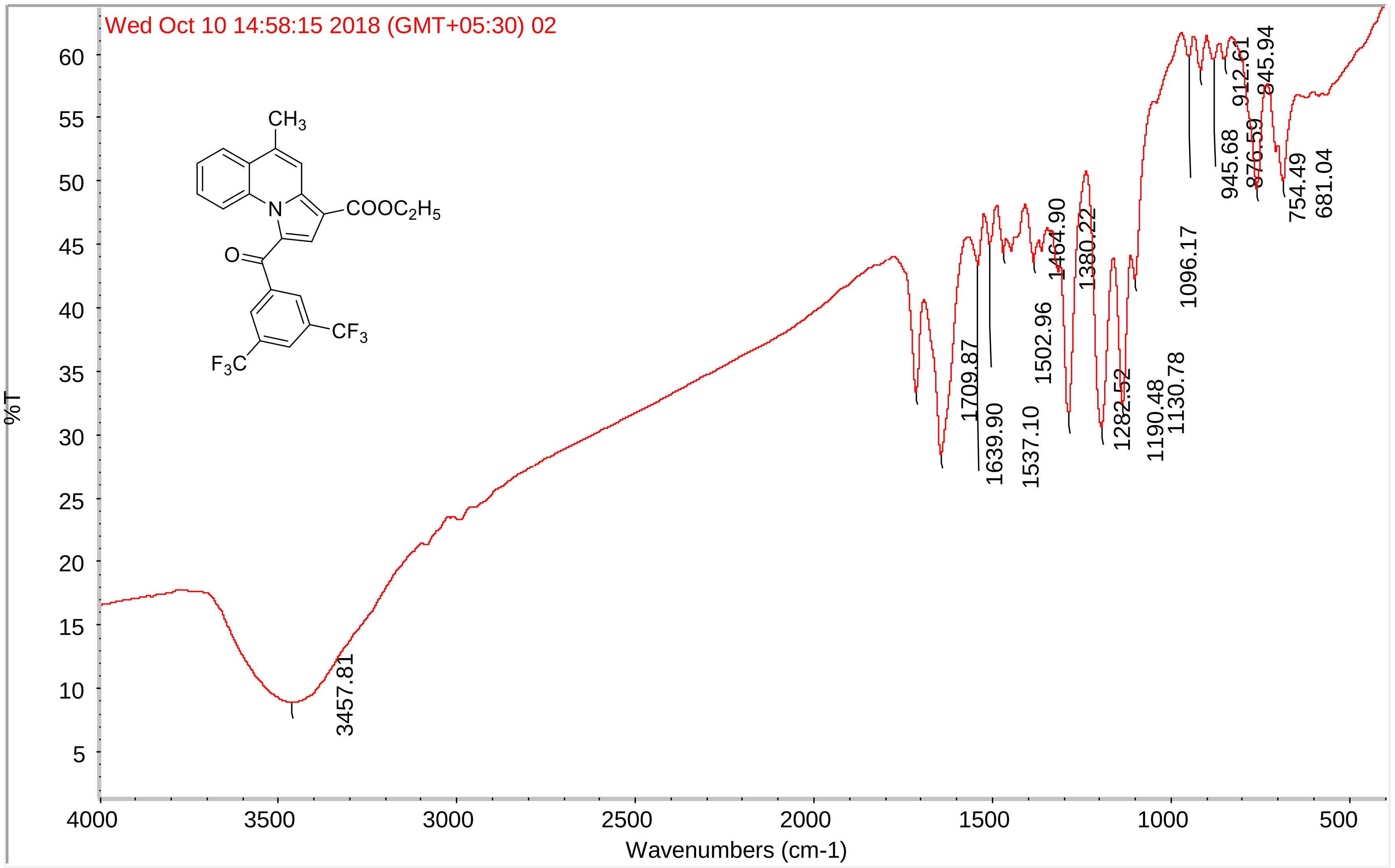

Figure S25: FT-IR of ethyl-1-(3,5-bis(trifluoromethyl)benzoyl)-5-methylpyrrolo[1,2-*a*]quinoline-3-carboxylate (**4i**)

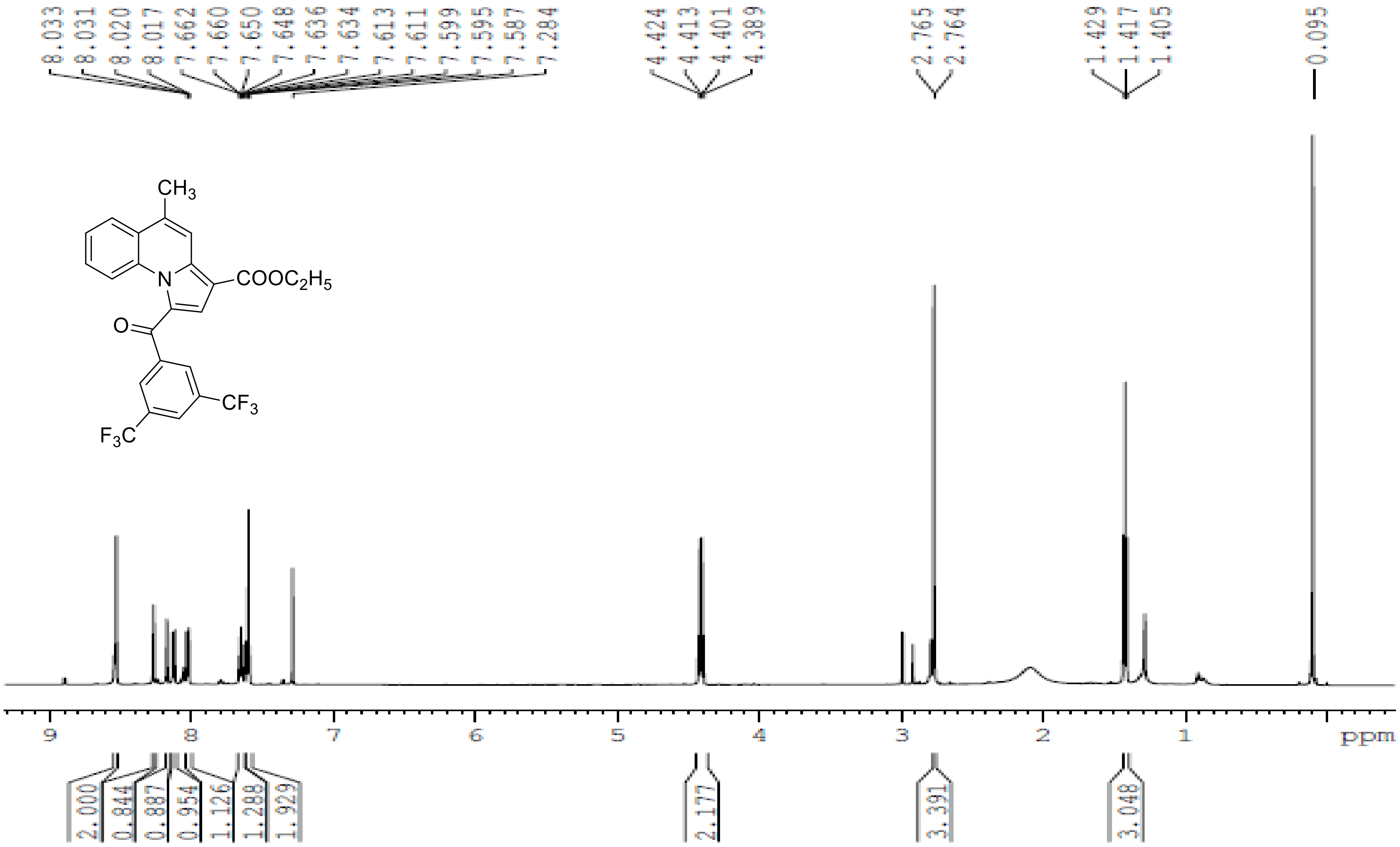

Figure S26: ^{1}H-NMR of ethyl-1-(3,5-bis(trifluoromethyl)benzoyl)-5-methylpyrrolo[1,2-*a*]quinoline-3-carboxylate (**4i**)

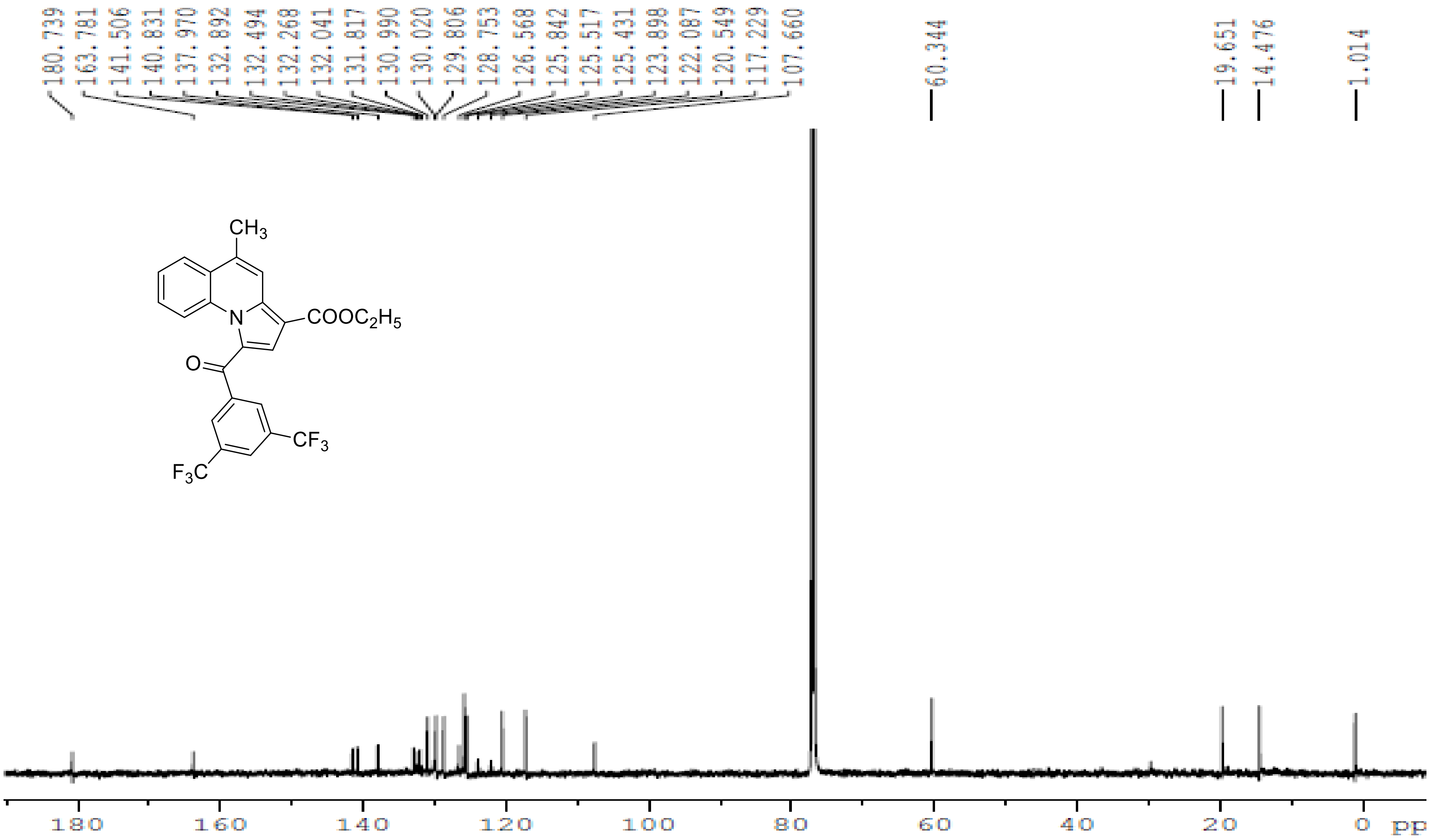

Figure S27: ^{13}C-NMR of ethyl-1-(3,5-bis(trifluoromethyl)benzoyl)-5-methylpyrrolo[1,2-*a*]quinoline-3-carboxylate (**4i**)

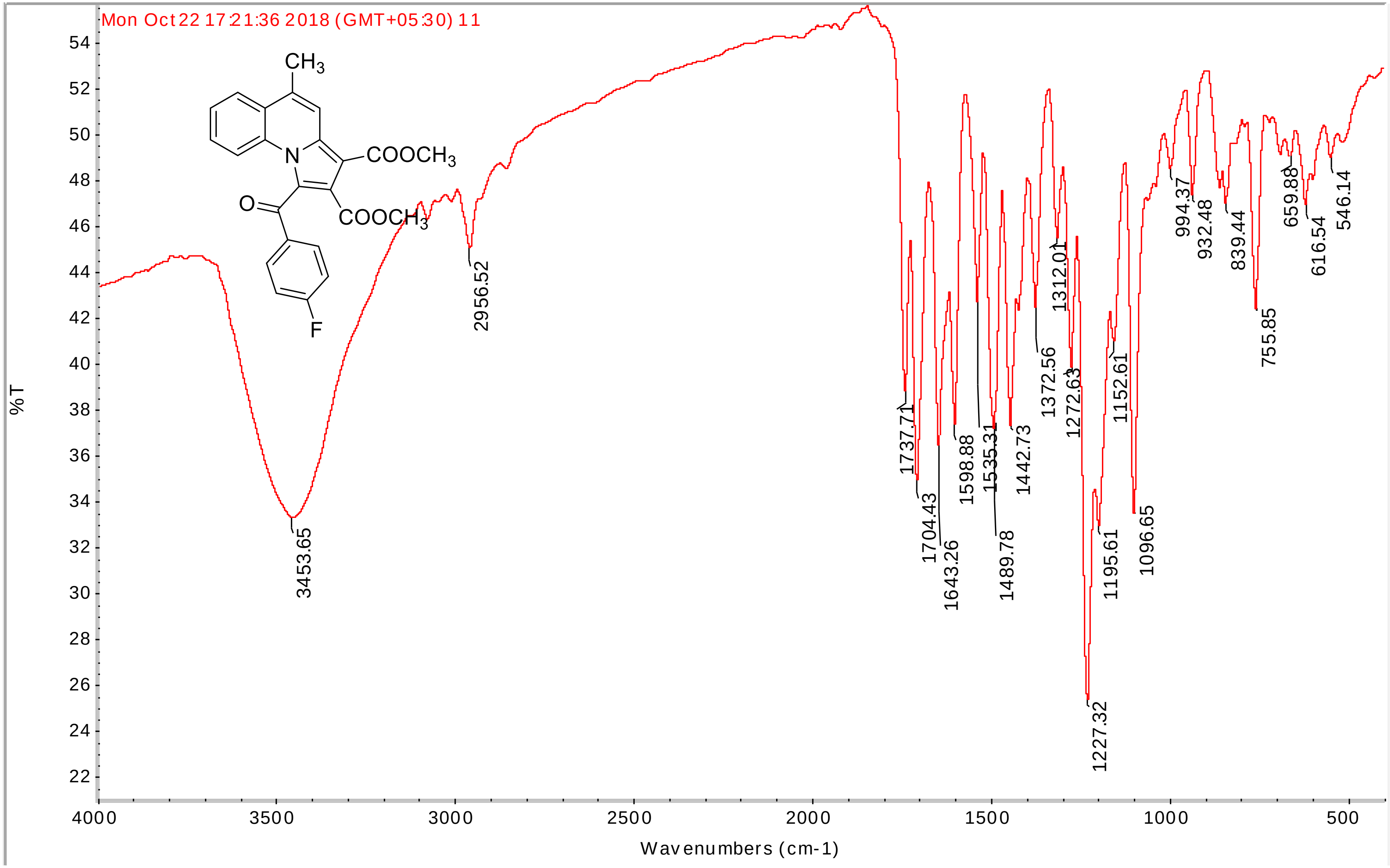

Figure S28: FT-IR of dimethyl-1-(4-fluorobenzoyl)-5-methylpyrrolo[1,2-*a*]quinoline-2,3-dicarboxylate (**4j**)

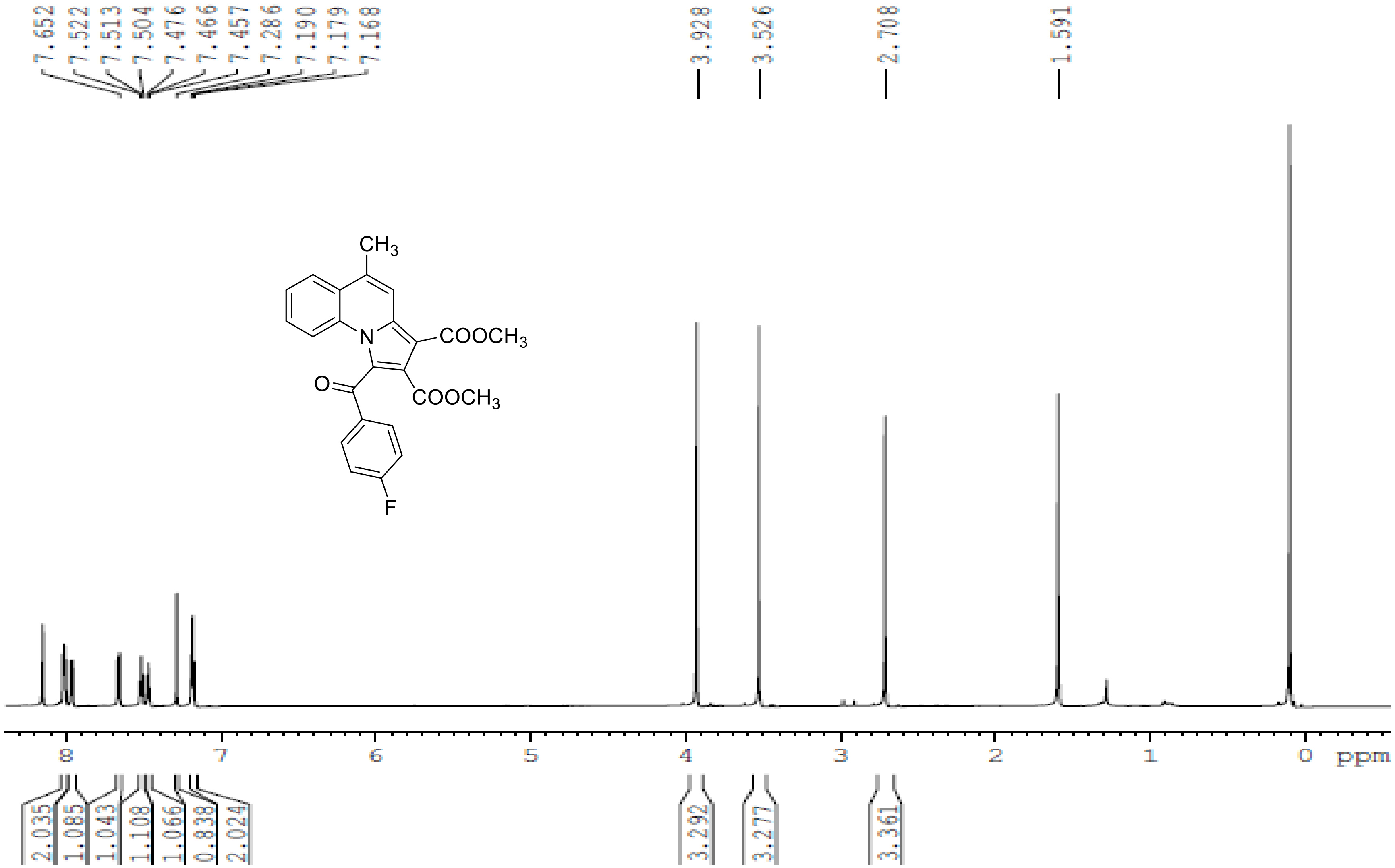

Figure S29: ^{1}H-NMR of dimethyl-1-(4-fluorobenzoyl)-5-methylpyrrolo[1,2-*a*]quinoline-2,3-dicarboxylate (**4j**)

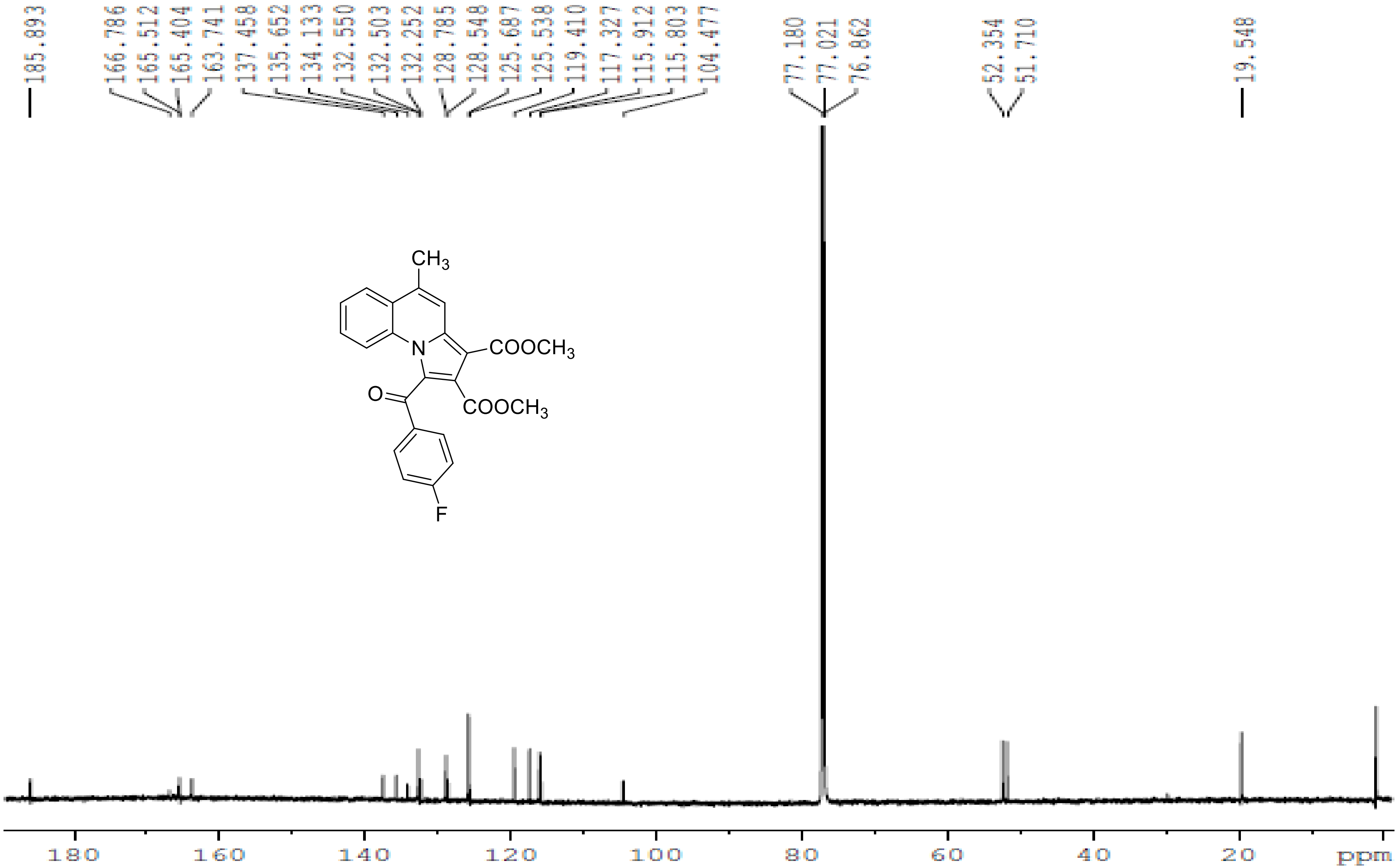

Figure S30: ^{13}C-NMR of dimethyl-1-(4-fluorobenzoyl)-5-methylpyrrolo[1,2-*a*]quinoline-2,3-dicarboxylate (**4j**)

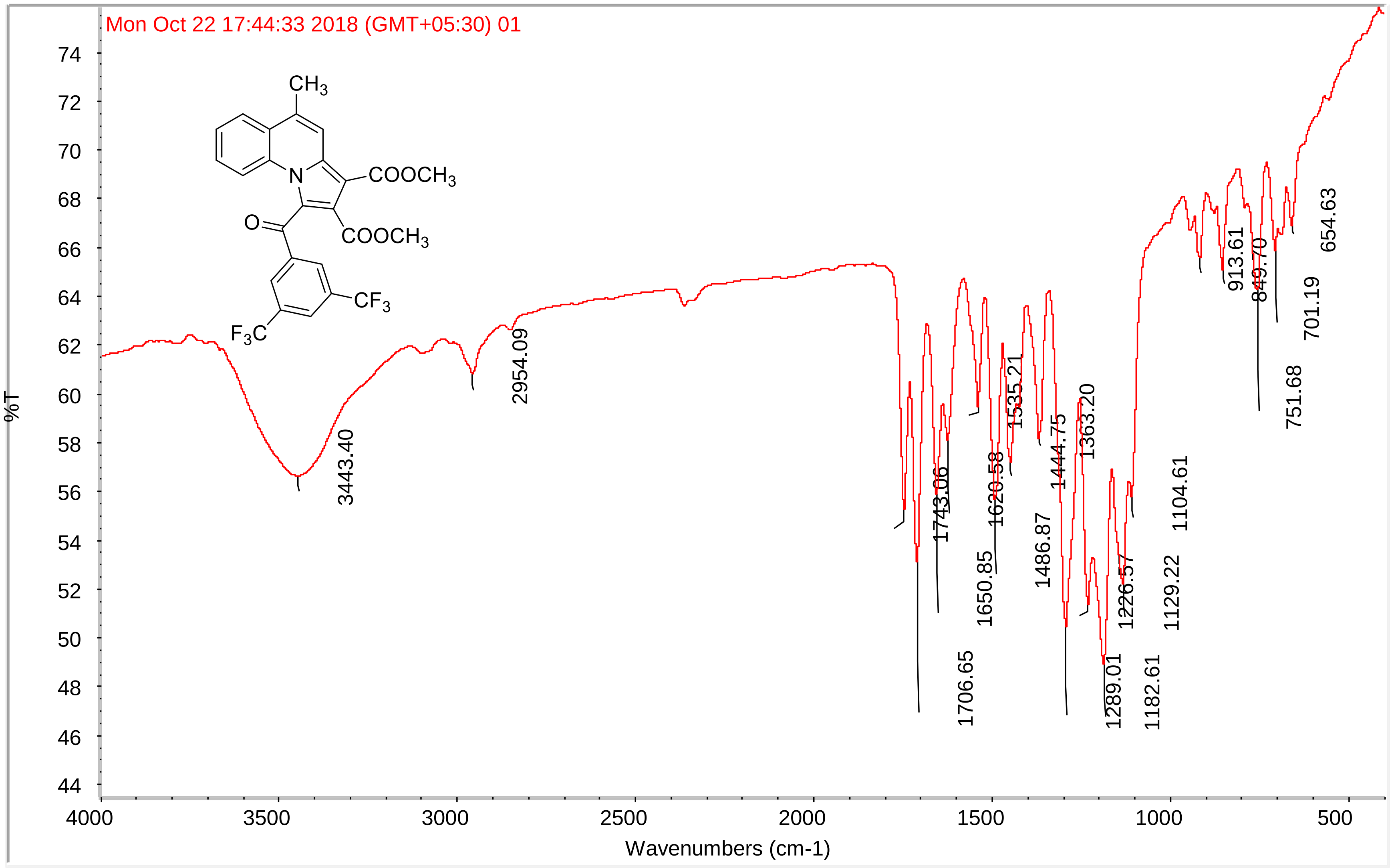

Figure S31: FT-IR of dimethyl-1-(3,5-bis(trifluoromethyl)benzoyl)-5-methylpyrrolo[1,2-*a*]quinoline-2,3-dicarboxylate (**4k**)

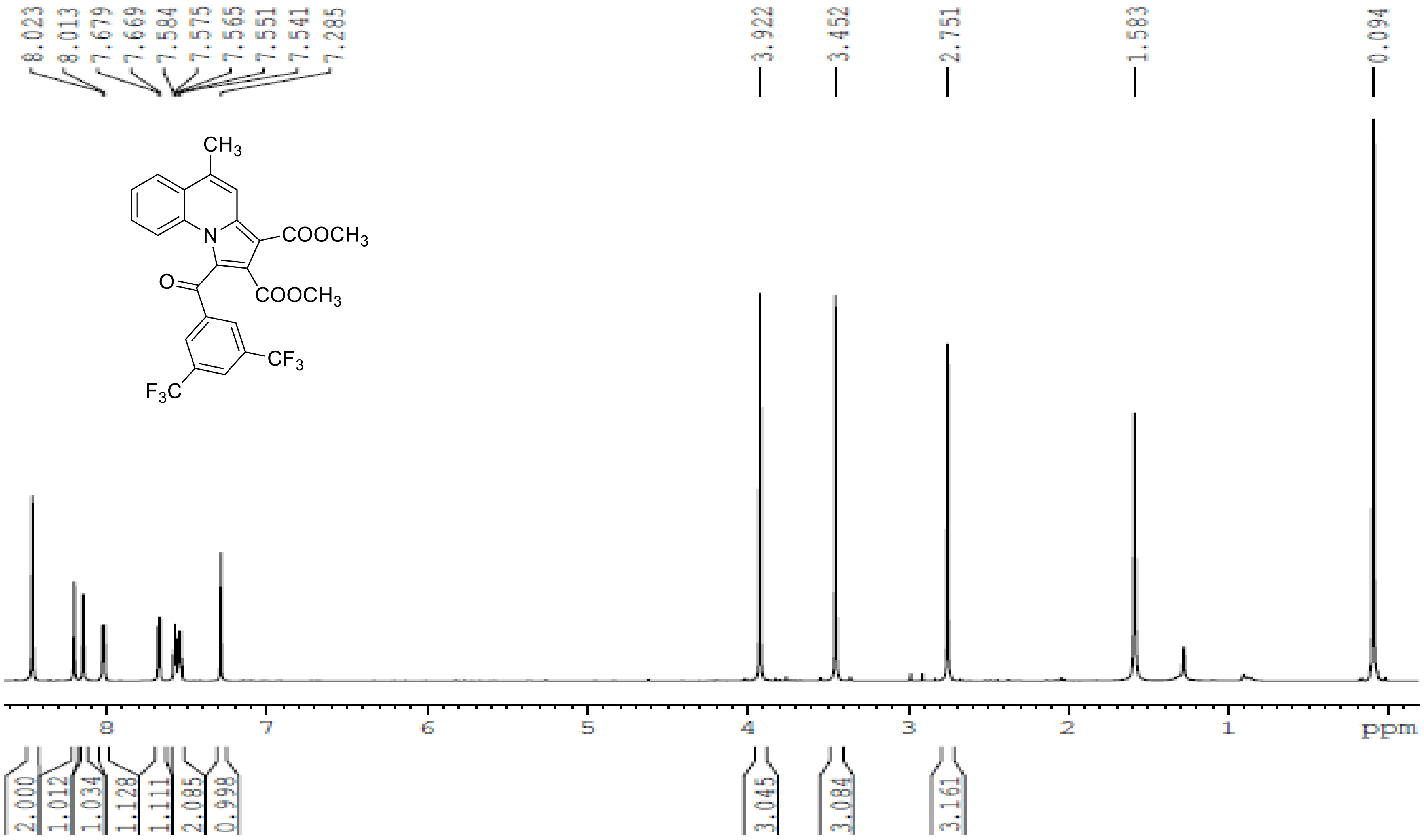

Figure S32: ^{1}H-NMR of dimethyl-1-(3,5-bis(trifluoromethyl)benzoyl)-5-methylpyrrolo[1,2-*a*]quinoline-2,3-dicarboxylate (**4k**)

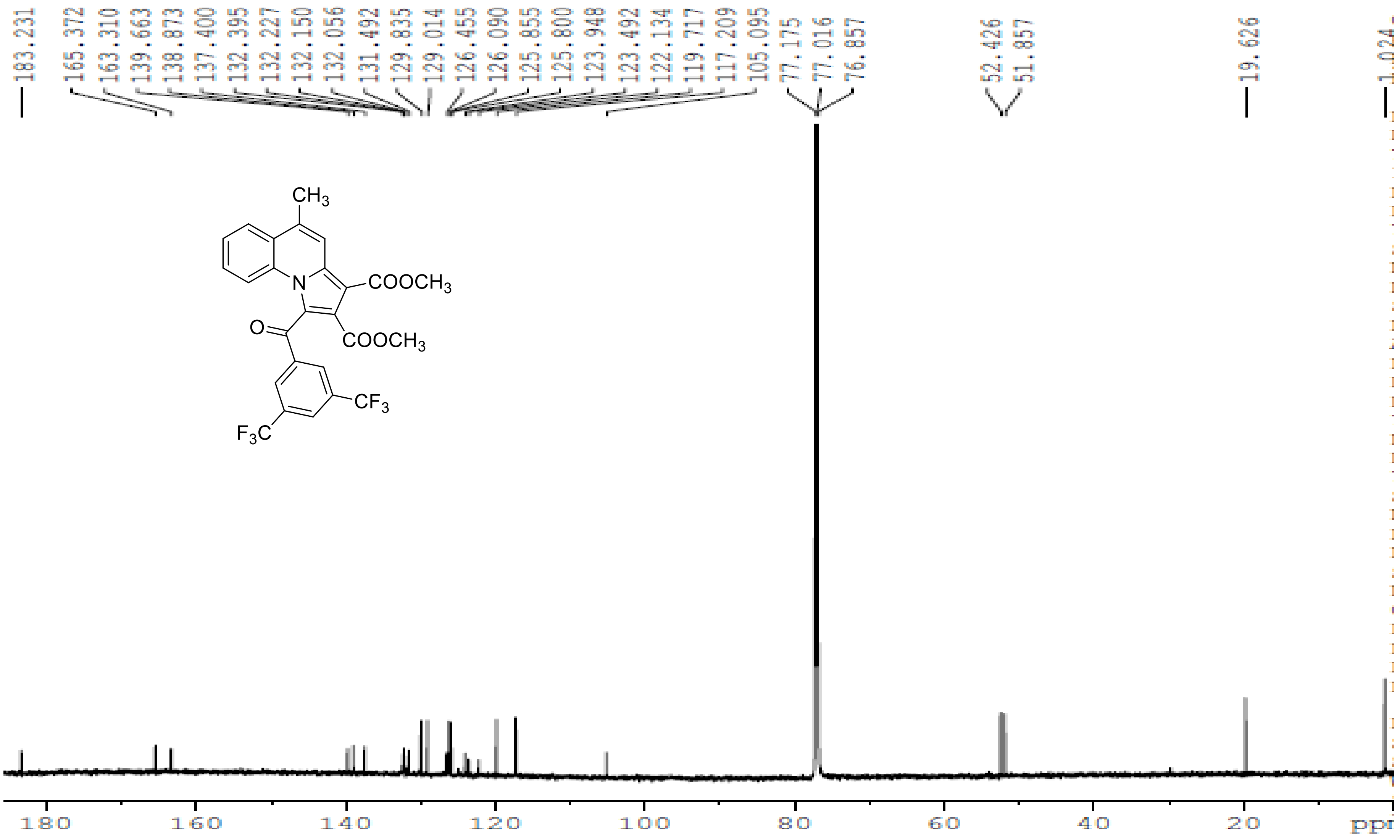

Figure S33: ^{13}C-NMR of dimethyl-1-(3,5-bis(trifluoromethyl)benzoyl)-5-methylpyrrolo[1,2-*a*]quinoline-2,3-dicarboxylate (**4k**)

References

1. Kemnitzer, W.; Kuemmerle, J.; Jiang, S.; Sirisoma, N.; Kasibhatla, S.; Crogan-Grundy, C.; Tseng, B.; Drewe, J.; Cai, S.X. Discovery of 1-benzoyl-3-cyanopyrrolo[1,2-a]quinolines as a new series of apoptosis inducers using a cell- and caspase-based high-throughput screening assay. 2: Structure-activity relationships of the 4-, 5-, 6-, 7- and 8-positions. *Bioorg Med Chem Lett* **2009**, *19*, 3481-3484, doi:10.1016/j.bmcl.2009.05.012.
2. Bakshi, D.; Singh, A. Transition-Metal-Free Synthesis of Nitrogen Containing Heterocycles With Fully Substituted N-fused Pyrrole Rings. *Asian Journal of Organic Chemistry* **2016**, *5*, 70-73, doi:10.1002/ajoc.201500324.
3. Bahner, C.T.; Barclay, L.R.; Biggerstaff, G.; Bilancio, D.E.; Blanc, G.W.; Close, M.; Isenberg, M.M.; Pace, E. Compounds for Cancer Studies 1. *Journal of the American Chemical Society* **1953**, *75*, 4838-4839, doi:10.1021/ja01114a510.
4. Uppar, V.; Chandrashekharappa, S.; Venugopala, K.N.; Deb, P.K.; Kar, S.; Alwassil, O.I.; Gleiser, R.M.; Garcia, D.; Odhav, B.; Mohan, M.K., et al. Synthesis and characterization of pyrrolo[1,2-*a*]quinoline derivatives for their larvicidal activity against *Anopheles arabiensis*. *Structural Chemistry* **2020**, doi:10.1007/s11224-020-01516-w.
5. Hu, H.; Feng, J.; Zhu, Y.; Gu, N.; Kan, Y. Copper acetate monohydrate: a cheap but efficient oxidant for synthesizing multi-substituted indolizines from pyridinium ylides and electron deficient alkenes. *RSC Advances* **2012**, *2*, 8637-8644, doi:10.1039/C2RA21213G.
6. Wang, C.; Hu, H.; Xu, J.; Kan, W. One-pot synthesis of indolizine via 1,3-dipolar cycloaddition using a sub-equivalent amount of K2Cr2O7 as an efficient oxidant under base free conditions. *RSC Advances* **2015**, *5*, 41255-41258, doi:10.1039/C5RA06019B.
